# Genetic dissection of cold stress tolerance and yield potential under cold stress in nested synthetic wheat *(Triticum aestivum L.)* introgression libraries using multi-locus genome-wide association and haplotype analysis

**DOI:** 10.1007/s10142-026-01925-w

**Published:** 2026-06-23

**Authors:** Mukesh Rathore, Nikita Aggarwal, Abhishek Pandey, Satinder Kaur, Mohd. Ashraf Bhat, Sundeep Kumar, Mohd Anwar Khan, Parvaze Ahmad Sofi, Reyazul Rouf Mir

**Affiliations:** 1 Division of Genetics & Plant Breeding, Faculty of Agriculture (FoA), SKUAST-Kashmir, Wadura, Campus, Sopore-193201, Kashmir, J&K India; 2 School of Agricultural Biotechnology, Punjab Agricultural University, Ludhiana, 141004 India; 3 ICAR-National Bureau of Plant Genetic Resources (NBPGR), Pusa Campus, New Delhi, India; 4 Centre for Crop and Food Innovation, WA State Agricultural Biotechnology Centre, Murdoch University, Murdoch, WA 6150 Australia

**Keywords:** Wheat, Cold stress, Rice–wheat crop rotation, GWAS, Haplotype analysis, Candidate genes

## Abstract

Cold stress is a major abiotic constraint to wheat production in temperate regions, particularly in the Kashmir Valley of north-western Himalayas, India, where prolonged low temperatures delay growth, reduce yield, and disrupt the rice–wheat cropping system. To address this challenge, we evaluated a panel of 340 nested synthetic hexaploid wheat introgression lines, developed by introgressing allelic diversity from *Aegilops tauschii* and *Triticum durum* into bread wheat. The panel was phenotyped for cold stress tolerance, electrolyte leakage index, and yield-related traits across three temperate environments and genotyped using the 35 K Axiom® Wheat Breeder’s Array. Genome-wide association study using FarmCPU and BLINK models identified 152 stable marker–trait associations (MTAs), with − log₁₀(P) values ranging from 3.00–14.64. Several stable MTAs surpassed the Bonferroni-adjusted significance threshold and were designated as high-confidence loci. A number of stable MTAs co-localized with previously reported genomic regions, whereas others represent potentially novel loci. Multiple pleiotropic loci were detected, indicating potential for simultaneous improvement of multiple traits. Haplotype analysis identified 32 major LD blocks significantly associated with target traits, with favorable haplotypes conferring enhanced stress tolerance and productivity. Several putative candidate genes were identified within chromosome-specific LD intervals of high-confidence MTAs. Literature mining, in silico expression profiling and network analyses further supported their functional relevance in cold stress adaptation and yield regulation. The identified genomic regions, haplotypes, and candidate genes provide valuable resources for marker-assisted and haplotype-based breeding to develop cold-tolerant, high-yielding wheat cultivars suited to temperate regions, thereby supporting the sustainability of rice–wheat cropping systems.

**Supplementary Information:**

The online version contains supplementary material available at 10.1007/s10142-026-01925-w.

## Introduction

Bread wheat (*Triticum aestivum* L.) is the most extensively cultivated cereal grain worldwide and occupies a central place in global agriculture (Khalid et al. 2022). It serves as a staple food for ~ 36% of the world’s population, with estimated global production of 799.9 million tonnes in 2024–25 (USDA 2024). Wheat contributes approximately 55% of dietary carbohydrates and 21% of global food calories (Riaz et al. 2021). With the global population projected to surpass 9.7 billion by 2050, wheat demand is expected to increase by ~ 70% (Anwar et al. 2024). These estimates cannot be attained unless efforts are intensified to develop cultivars capable of withstanding a wide range of biotic and abiotic stresses, which together account for considerable yield losses (Khan et al. 2024; Gudi et al. 2025a, b).

Among the abiotic stresses, extreme weather events driven by climate change are becoming increasingly common, causing severe episodes of extreme cold temperatures injury to unacclimated plants (Chang et al. 2021). Based on temperature ranges, cold stress is classified as chilling stress (0–12 °C) or freezing stress (below 0 °C, more detrimental) (Wang et al. 2016; Wu et al. 2024). Freezing stress induces the formation of intracellular or extracellular ice crystals, which damage structural integrity of the cellular framework of plants, cause electrolyte leakage, disturb ion homeostasis, and may ultimately trigger programmed cell death (Jahed et al. 2023). Overwintering wheat, which contributes ~ 75% of global wheat yield and is predominantly grown in higher latitudes, is particularly vulnerable to frost injury, with losses reaching up to 80% under severe conditions (Fowler 2012; Xiao et al. 2022; Soleimani et al. 2022). Most of the wheat-growing areas worldwide often undergo low-temperature stress and annually results in millions of tonnes of grain yield losses, including China (Yue et al. 2016; Xiao et al. 2018), the United States (Centinari et al. 2016), Europe (Trnka et al. 2014; Ma et al. 2019), and Australia (Zheng et al. 2015; Crimp et al. 2016).

India is the world’s second-largest wheat producer after China, recording a production of ~ 112.9 million tonnes in 2023–24 (Jan et al. 2025). Wheat is primarily cultivated in the northern plains such as Uttar Pradesh, Madhya Pradesh, Punjab, Haryana, Rajasthan, Bihar, Gujarat, and Maharashtra during the winter season (October to March), when average temperatures range from 10–15 °C. However, wheat is also cultivated in diverse ecologies, including tropical, subtropical, temperate, and cold regions, extending up to 67°N in Jammu and Kashmir (J&K). In J&K, wheat is grown on ~ 281.87 thousand hectares, with an average yield of 19.33 q/ha (Qammer et al. 2022). Cultivation is largely confined to the subtropical Jammu region, with only ~ 1,300 ha in the Kashmir Valley (Mahdi et al. 2025). The Kashmir Valley, a fragile cold-arid ecosystem in the North Western Himalayas (Banerji and Basu 2010; Chevuturi et al. 2018; Jan et al. 2023a, b; 2026), is highly suitable for realizing higher yields of wheat due to precipitation from December to May coinciding with critical growth stages. However, yields remain low despite favorable conditions, as the crop is exposed to extreme cold (0 °C or below) during early developmental stages, leading to poor seedling emergence, reduced vigor, leaf yellowing, delayed vegetative growth, disturbed water relations, seed abortion, and reduced seed size, ultimately leads to severe reduction in yield (Jan et al. 2023a, b; 2026). Reports indicate that temperatures of − 5 °C to − 7 °C during the vegetative stage can reduce yields by 10–100% (Fuller et al. 2007; Su et al. 2022a, b). Moreover, delayed vegetative growth extends the crop duration to 245–250 days, leaving insufficient time for rice cultivation, the preferred rotation crop in the region and does not ensure wheat-rice crop rotation, as in other parts of India.

As cold stress cannot be chemically or manually controlled, there is an urgent need to identify and characterize genotypes with both frost tolerance and high yield potential under cold stress. Clarifying the genetic mechanisms underlying cold tolerance will facilitate the development of wheat varieties with freezing resistance, early maturity, and stable yields under temperate conditions, one of the key objectives of wheat improvement in Kashmir. Cold tolerance and yield are complex polygenic traits; thus, high-throughput genotyping combined with genome-wide association studies (GWAS) has emerged as the preferred approach over traditional linkage mapping, enabling the identification of robust marker–trait associations (MTAs) (Rahimi et al. 2023; Rathan et al. 2022; Hao et al. 2024; Bashir et al. 2025). Numerous QTLs/genomic regions for cold tolerance have been identified globally on chromosomes 1 A, 1D, 2 A, 2B, 3 A, 5 A, 5B, 6 A, 6B, 6D, and 7B (Kruse et al. 2017; Zhao et al. 2020a; Pang et al. 2021; Chen et al. 2021; Soleimani et al. 2022; Vaitkevičiūtė et al. 2023; Pan et al. 2024) and are being exploited in breeding programs. Despite this, sufficient winter hardiness and yield stability remain challenging in modern cultivars (Fowler 2012; Sthapit Kandel et al. 2018; Jan et al. 2023a). Broader exploitation of genetic diversity from wheat progenitors may contribute to further development and is therefore essential. The limited progress in improving abiotic stress tolerance in modern cultivars is partly due to the historical emphasis of wheat breeding programs majorly on biotic stresses, coupled with genetic bottlenecks from domestication that significantly narrowed down the genetic diversity relative to wild ancestors (Pont et al. 2019).

To address this gap, we employed a unique germplasm of nested synthetic hexaploid wheat introgression lines (S-NILibs), developed by introgressing allelic diversity from *Aegilops tauschii* (D-genome donor) and *Triticum durum* (AB-genome donor) into bread wheat (Kaur et al. 2025). These progenitors harbor substantial genetic variation for cold tolerance and yield under abiotic stress, accumulated over long evolutionary histories (Barashkova 1980; Limin and Fowler 1985; Badawi et al. 2007; Jia et al. 2013; Masoomi-Aladizgeh et al. 2015; Kaur et al. 2018; Bhardwaj & Chaudhry 2025). The S-NILibs panel displayed significant variation for the studied traits, making it well-suited for GWAS and candidate gene discovery. In addition to GWAS, haplotype analysis of SNP markers offers advantages over single-SNP approaches by enabling the identification of superior haplotypes within genomic regions governing target traits. These superior haplotypes can subsequently be deployed in haplotype-assisted breeding programs. To our knowledge, limited studies have explored MTAs, candidate genes and favourable haplotypes for cold stress resistance and yield under cold stress using S-NILibs in wheat from the North-Nestern Himalayas, India.

To address the limited understanding of the genetic basis of cold tolerance, the present study evaluated 340 S-NILibs with three specific objectives: (i) to assess phenotypic responses for cold stress tolerance, grain yield, and yield component traits under temperate field conditions; (ii) to identify significant MTAs and superior haplotypes associated with these traits and evaluate their stability across environments; and (iii) to identify and prioritize candidate genes underlying reliable MTAs. The panel was evaluated across three temperate environments and genotyped using the 35 K Axiom® Wheat Breeder’s Array. Yield, a complex polygenic trait, was dissected into agro-morphological and physiological components, including days to 50% flowering (DTF), days to maturity (DTM), flag leaf area (FLA), peduncle length (PDL), plant height (PH), spike length (SL), spikelet number per spike (SNS), grains per spike (GPS), thousand grain weight (TGW), and yield per plant (YPP) (Sukumaran et al. 2018).

The findings of this study are expected to facilitate wheat improvement and help sustain the rice–wheat cropping system in Kashmir and other regions exposed to prolonged cold stress, thereby contributing to the development of resilient and high-yielding cultivars.

## Material and methods

### Association mapping panel

The present study utilized a panel of 340 S-NILibs, along with four parental lines: PDW233/*Ae. tauschii* acc. PAU 14135 (Syn14135), PBW114/*Ae. tauschii* acc. PAU 14170 (Syn14170), BWL3531 (PBW343 + Yr17 + Yr70 + Lr76 = Unnat PBW343), and BWL4444 (HD2967 + Yr10), procured from the School of Agricultural Biotechnology, Punjab Agricultural University, Ludhiana. The panel also included a local check cultivar, Shalimar Wheat-2 (SW-2).

The S-NILibs were developed from four crosses involving two synthetic hexaploid wheat lines and two advanced hexaploid wheat lines as follows: Cross 1 (Syn14135 × BWL4444–74 lines), Cross 2 (Syn14135 × BWL3531–102 lines), Cross 3 (Syn14170 × BWL4444–80 lines), and Cross 4 (Syn14170 × BWL3531–84 lines). Detailed information on the panel and the breeding scheme used to develop the S-NILibs is provided in Kaur et al. (2025). Although cold resistance was not the primary breeding objective during the development of this population, the panel was selected for the present study because previous studies have demonstrated significant natural variation for multiple abiotic and biotic stress–related traits (Sandhu et al. 2021, 2023; Kumar et al. 2022; Sharma et al. 2022; Kaur et al. 2025).

### Experimental layout and field conditions

The panel was evaluated across three temperate environments over two consecutive crop seasons: 2022–2023 (E1) and 2023–2024 (E2) at the Faculty of Agriculture, Wadura (34.34° latitude, 74.40° longitude), and during 2023–2024 (E3) at the Dryland Agricultural Research Station, Budgam (34.52° latitude, 74.47° longitude), SKUAST-Kashmir. Seeds were sown between 9 and 11 October across all environments.

The experiment followed an Augmented Block Design (ABD) comprising 17 blocks. Each block contained 20 S-NILibs and five check entries (the four parental lines and one local check, SW-2). Standard agronomic practices were followed throughout the crop season. Pests and diseases were managed chemically, while weeds were controlled both manually and chemically to maintain a healthy crop stand.

From November to February, the Kashmir Valley experiences a marked decline in temperature, frequently ranging from 0 to − 7 °C. During this period, wheat growth slows considerably and remains at the seedling to early tillering stage, corresponding to Zadoks stages 13–21. These prolonged low-temperature conditions result in significant freezing injury, providing an ideal field environment for evaluating cold stress tolerance. Detailed information on the environments, sowing dates, minimum temperature exposure, and phenotyping timelines is summarized in Table 1. The mean maximum and minimum temperatures recorded at each environment during the cropping seasons are presented in Fig. S1.

**Table 1:** Details of the experimental material, experimental design, location, sowing dates, minimum temperature exposure, and phenotyping timelines for the studied traits across three environments (E1, E2, and E3)

Population Details	Environment	Experimental Design & Location	Sowing Date	Minimum Temperature Exposure	Phenotyping Timlines for Cold Stress	Phenotyping Timlines for Yield and Yield Related Traits (From Flowering to Harvesting)
Pop-1 (PDW233-*Ae. tauschii* acc. pau14135 amphiploid//BWL4444); Pop-2 (PDW233-*Ae. tauschii* acc. pau14135 amphiploid//BWL3531); Pop-3 (PBW114-*Ae. tauschii* acc. pau14170 amphiploid//BWL4444); Pop-4 (PBW114-*Ae. tauschii* acc. pau14170 amphiploid//BWL3531)	E1	Augmented design in 2022–23 [Faculty of Agriculture, Wadura (34.34°Lattitude, 74.40°Longitude)]paired row (1.5 m long with 20 cm row to row spacing with standard agronomic practices	10/11/2022	(- 2 °C)	15 December—27 Febuary (15 days interval)	19–03–2023—10–07–2023
	E2	Augmented design in 2023–24 [Faculty of Agriculture, Wadura (34.34°Lattitude, 74.40°Longitude)]paired row (1.5 m long with 20 cm row to row spacing with standard agronomic practices	10/9/2023	(−4 °C)		14–03–2024—15–07–2024
	E3	Augmented design in 2023–24 Dryland Agricultural Research Station (DARS), Budgam (34.52°Lattitude, 74.47°Longpaired row (1.5 m long with 20 cm row to row spacing with standard agronomic practicesitude)]	10/11/2023	(- 7 °C)		17–03–2024—12–07–2024

### Phenotypic evaluation for cold stress tolerance

Cold stress tolerance (CS) was evaluated at the seedling to early tillering stage (Zadoks 13–21) under natural field conditions using a 0–4 visual injury scale, as proposed by Zhao et al. (2020a). Scoring was based on visible freezing injury symptoms, including leaf chlorosis, necrosis, and whole-plant mortality.

**Fig. 1:**
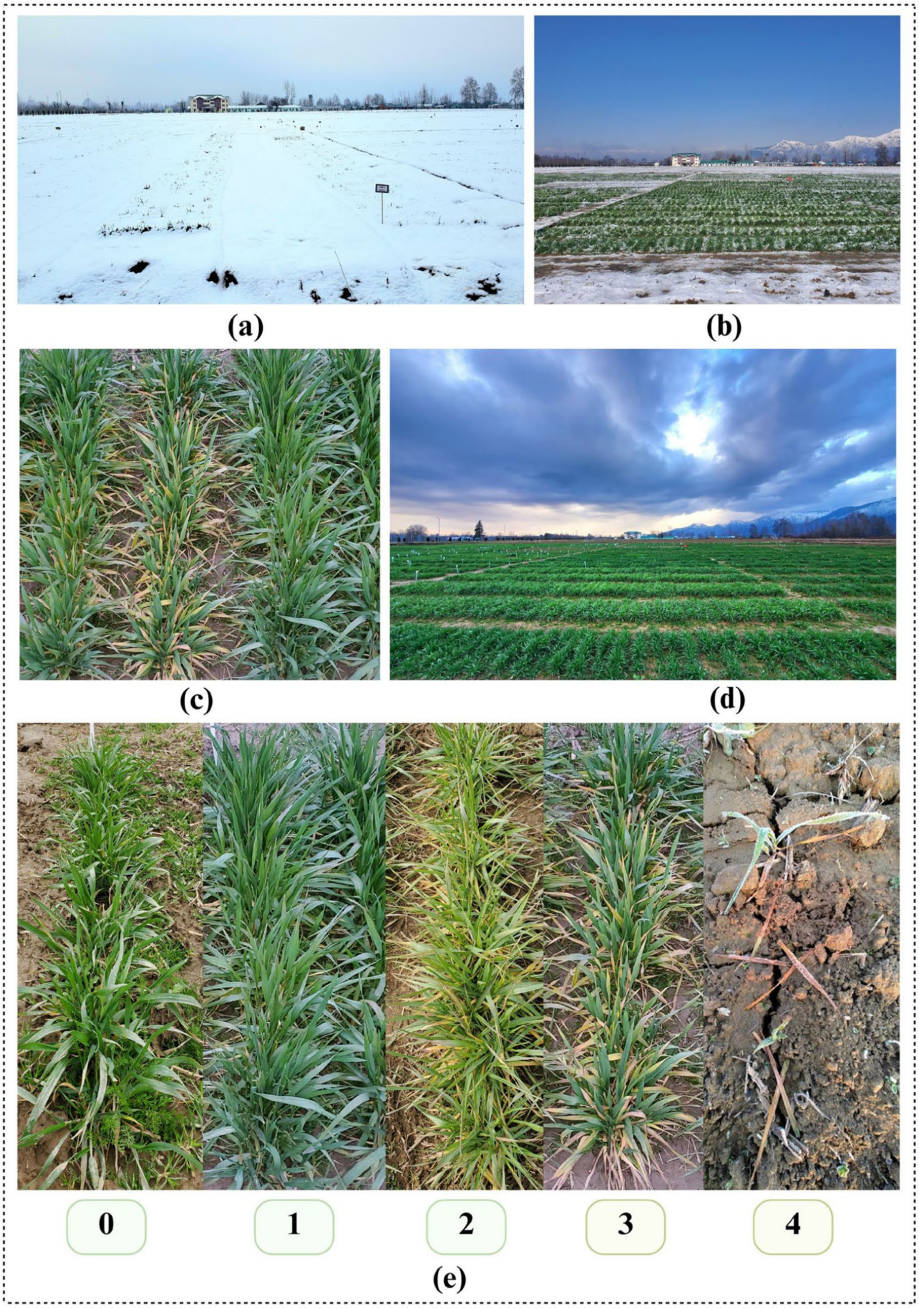
Field experimental conditions and cold tolerance responses of wheat genotypes across environments. (**a**) Experimental field covered with snow during the winter season, illustrating exposure to low-temperature stress. (**b**) Partial snow-melt stage showing the resumption of crop growth under fluctuating temperature conditions. (**c**) Close-up view of wheat plants showing contrasting genotypes resistant and susceptible to low-temperature stress. (**d**) Overview of the experimental field under normal growing conditions following cold stress exposure. (**e**) Phenotypic variation among wheat genotypes scored on a 0–4 scale, where 0 = highly tolerant, 1 = tolerant, 2 = moderately tolerant, 3 = susceptible, and 4 = highly susceptible, based on visual symptoms of stress-induced damage

A score of 0 indicated no visible freezing injury, resulting in a predominantly green canopy. A score of 1 corresponded to injury restricted to leaf tips, while older leaves remained largely unaffected. A score of 2 indicated partial injury, with the majority of leaf area, particularly young leaves, remaining unfrozen, while dead yellow leaves were scattered on the ground. A score of 3 represented severe injury, characterized by a large proportion of frozen leaves forming the majority of intact leaves on the ground. A score of 4 was assigned to completely frozen and necrotic leaves or, in severe cases, complete plant death (Fig. 1).

Phenotypic assessments were conducted at 15-day intervals starting from mid-December, following the appearance of clear freezing injury symptoms on the local susceptible check (SW-2), and continued until February. During this period, minimum temperatures consistently ranged from approximately 0 to − 7 °C, ensuring sustained cold stress exposure. Although injury symptoms were monitored across multiple time points, only the final assessment recorded in February was used for statistical analyses, as it reflected the cumulative and stable expression of cold injury prior to spring regrowth. All scoring was performed by a single trained evaluator to minimize inter-rater variation. To further reduce subjective bias, genotype identities were blinded during evaluation and plants were assessed using coded identifiers.

To validate the reliability of visual field scoring, electrolyte leakage index (EL) was measured as an indicator of cold-induced membrane damage following established protocols (Mir et al. 2021). Fully expanded seedling leaves were collected from the field in February for each genotype, with two biological replicates. Leaf tissues (1 g) were thoroughly washed with deionized water to remove surface-adhered electrolytes and then placed in sealed glass vials containing 10 mL deionized water. Samples were incubated at room temperature for 30 min on a rotary shaker at 150 rpm, after which initial electrical conductivity (L1) was measured using a conductivity meter (µS cm⁻^1^). Subsequently, samples were autoclaved at 120 °C for 10 min to ensure complete cellular disruption, cooled to room temperature, shaken again for 30 min, and the final conductivity (L2) was recorded. The EL was calculated as:

$$\mathrm{E}\mathrm{L}\left(\%\right) =\left(\mathrm{L}1/\mathrm{L}2\right)\times 100$$

The EL represents the proportion of electrolytes released due to cold-induced membrane injury relative to the total electrolytes released after complete tissue disruption, thereby serving as a quantitative measure of cold stress damage. Mean EL values from biological replicates were used for subsequent statistical analyses.

### Phenotypic evaluation for yield and component traits

For each of the 3 field trials, the phenotypic data were recorded on the following 10 agronomic traits; DTF was calculated as days from the date of sowing until 50% of the spikes within a row had emerged from the flag leaf sheath. FLA (cm^2^) was calculated as flag leaf length × flag leaf width × 0.83. DTM was recorded when the majority of plants in the row showed a complete loss of green colour from the flag leaf. PH (cm) was recorded at physiological maturity as the average of randomly selected five plants per genotype by measuring from the soil surface to the spike tip excluding awns. PDL (cm) was measured on the same plants used for PH. It is the length of stalk exposed from the flag leaf to the base of the spike. SL (cm) was determined as the average length of five main spikes, measured from the spike base to the tip, excluding awns. SNS was measured as the average number of spikelets present on the main five same spikes. GPS was calculated as the average of grain number present on the main five same spikes. TGW was recorded by weighing 1000 grains in grams (g) post-harvest. Mean YPP (g) was calculated as grain yield per row/number of plants per row.

### Phenotypic data analysis

Field experiments were conducted using an augmented block design (ABD), in which test genotypes were unreplicated, while check cultivars were replicated across blocks. Phenotypic data were analyzed separately for each environment (E1, E2, E3) and combined environment (CE). To obtain CE phenotypes, raw observations for each genotype were averaged across the three environments (E1, E2, and E3), and these averaged values were subsequently treated as an independent environment (CE). For each environment, ANOVA and check-adjusted genotype means were obtained by fitting the following linear model appropriate for an augmented block design (Federer 1956) using the ACBD-R package in R (R Core Team, 2020).

$${\mathrm{y}}_{\mathrm{i}\mathrm{j}}=\mu +\mathrm{g}\mathrm{i}+{\mathrm{c}}_{\mathrm{j}}+{\beta}_{\mathrm{j}}+{\varepsilon}_{\mathrm{i}\mathrm{j}}$$

where:

yij is the observation of the *i*th genotype in the *j*th block,

μ is the overall mean,

gi is the fixed effect of the *i*th test genotype,

cj is the fixed effect of the control (check) genotypes in the *j*th block,

βj is the random effect of the *j*th block,

εij is the random residual error.

Block effects and residual errors were treated as random, while genotype and check effects were considered fixed. Check-adjusted genotype means obtained from this model were used as phenotypic estimates for further statistical analyses.

For each environment, descriptive statistics including mean, minimum, maximum, standard error, and standard deviation were computed. Variance components were estimated to derive phenotypic variance (PV), genotypic variance (GV), and environmental variance (EV). These variance components were subsequently used to calculate the phenotypic coefficient of variation (PCV), genotypic coefficient of variation (GCV), coefficient of variation (CV), broad-sense heritability (h^2^), genetic advance (GA), and genetic advance as a percentage of the mean (GAM).

Trait distributions were visualized using box- plots to illustrate the median, interquartile range, spread and outliers. These plots were generated using the ggplot2 R package (Wickham 2016). In addition, Pearson correlation analysis and principal component analysis (PCA) were performed using R software.

### SNP genotyping, SNP calling and data filtration

Genomic DNA from the GWAS panel (340 S-NILs) was isolated from leaf tissues of two-week-old seedlings using the CTAB method (Saghai-Maroof et al. 1984). High-density genotyping was performed using the 35 K Axiom® Wheat Breeder’s Array (Affymetrix UK Ltd., United Kingdom) following standard Axiom sample preparation protocols (Allen et al. 2017; Burridge et al. 2017; 2018) and the manufacturer’s instructions (Axiom™ 2.0 Assay for 384HT Array Format Automated Workflow, P/N 703268, Rev. 5).

Briefly, the procedure involved DNA amplification, fragmentation, precipitation, resuspension, and hybridization, followed by hybridization-ready plate assembly and sample quality control. Samples were denatured and transferred to the hybridization tray, after which the hybridization tray and array plate were loaded into the GeneTitan MC instrument for final array processing.

SNP allele calling was performed using Axiom Analysis Suite v5.1 (Thermo Fisher Scientific) following the Axiom Best Practices Genotyping Workflow. A total of 35,143 SNPs were obtained from the array. Physical positions of SNP markers were retrieved from the CerealsDB database (www.cerealsdb.uk.net).

To remove low-quality markers, genotypic data were filtered using the TASSEL v5.2.57 pipeline based on the following criteria: minor allele frequency (MAF) > 0.05, maximum heterozygosity ≤ 0.10, and missing genotype rate < 0.10 (Kaur et al. 2025). After filtering, 9,348 high-quality SNPs were retained for downstream analyses.

### Population structure and kinship analysis

The filtered SNP dataset was used for assessing genetic diversity and to identify MTAs. Population structure was inferred using three complementary approaches: (i) Bayesian model-based clustering implemented in STRUCTURE v2.3.4 (Pritchard et al. 2000), (ii) a neighbor-joining phylogenetic tree constructed in TASSEL v5.2.57 (Bradbury et al. 2007) and visualized using MEGA v7.0 (Kumar et al. 2016), and (iii) principal component analysis (PCA) performed in GAPIT (Lipka et al. 2012). Pairwise genetic relatedness among genotypes was estimated using a kinship (K) matrix generated in TASSEL. Detailed descriptions of SNP quality control, population structure inference and kinship estimation have been reported previously in Kaur et al. (2025).

### Detection of marker trait associations

Genome-wide association studies (GWAS) were conducted using check-adjusted genotype means for each individual environment and for CE using the GAPIT v3.0 package implemented in R (Wang and Zhang 2021) with default parameters. Two multi-locus models, Fixed and Random Model Circulating Probability Unification (FarmCPU; Liu et al. 2016) and Bayesian Information and Linkage Disequilibrium Iteratively Nested Keyway (BLINK; Huang et al. 2019), were employed. These models are reported to provide higher statistical power and reduced false-positive rates compared with other models implemented in GAPIT v3.0 (Huang et al. 2019; Saini et al. 2022).

The Bonferroni-corrected significance threshold was calculated as P = 0.05/9,348 = 5.35 × 10⁻⁶, corresponding to a − log₁₀(P) value of 5.27. However, this threshold is relatively strict, as it assumes all SNPs represent independent tests, often resulting in a limited number of MTAs. Therefore, a dual-threshold strategy was adopted. Initially, a threshold of − log₁₀(P) ≥ 3.0 was applied to detect significant MTAs within individual environments, consistent with previously reported GWAS studies (Kaur et al. 2023; Zhao et al. 2023a; Gaur et al. 2024).

Significant MTAs consistently detected in at least two environments and by at least one model were considered stable and prioritized. Among these stable associations, MTAs exceeding the Bonferroni-corrected threshold were classified as high-confidence MTAs. Manhattan and Q–Q plots were generated using the CMplot R package to visualize significant associations.

### Haplotype-based association mapping

For haplotype analysis, genome-wide haplotypes were identified via Haploview v4.2 (Barrett et al. 2005). Linkage disequilibrium (LD) blocks were defined using the default confidence interval (CI) method, as described by Gabriel et al. (2002). In this method, a block was defined if 95% of the informative pairwise comparisons (i.e., non-inconclusive) showed strong LD, with the upper bound of the 95% confidence interval for D′ being > 0.98 and the lower bound being > 0.7. Markers with a MAF < 0.05 were excluded from the block definition process. LD blocks containing stable MTAs were specifically targeted for haplotype-based analysis (Wang et al. 2022a, b). A haplotype was considered major if it was present in at least 10 accessions. To evaluate haplotype effects, the following linear model was fitted for each LD block, with haplotypes as fixed effects and the first two principal components (PC1 and PC2) included as covariates to account for population structure.

$$\mathrm{y}\mathrm{i}=\upmu +{\mathrm{H}}_{\mathrm{j}}+ {\upbeta}_{1}PC{1}_{\mathrm{i}}+ {\upbeta}_{2}PC{2}_{\mathrm{i}}+ {\upvarepsilon}_{\mathrm{i}}$$

where:

yi = phenotypic value of the *i*-th genotype.

μ = overall mean.

H_j_ = fixed effect of the *j*-th haplotype.

β_1_, β_2_ = regression coefficients for PCs.

PC1_i,_ PC2_i_ = first two principal components (population structure covariates).

εi∼N (0, ${\upsigma}_{e}^{2}$) = residual error.

Estimated marginal means (EMMs) were calculated for each haplotype, and pairwise comparisons among haplotypes were performed. To account for multiple testing, p-values from pairwise contrasts were adjusted using the Benjamini–Hochberg false discovery rate (FDR) correction.

### Linkage disequilibrium (LD) analysis

LD was estimated for SNP pairs within a 100-kb window using TASSEL, and LD decay curves were generated in RStudio (Gudi et al. 2023). LD patterns were evaluated separately for individual wheat chromosomes in the current study, whereas LD estimates for sub-genome (A, B, and D) and whole-genome have been reported previously (Kaur et al. 2025).

### Candidate genes (CGs) identification, in sillico expression and network analysis

High-confidence MTAs were used to identify putative CGs. Genomic regions for CGs analysis were defined according to the chromosome-specific LD decay distances, and CGs were searched within the flanking regions corresponding to the half-LD decay interval around each high-confidence MTAs (Gudi et al. 2023). Putative CGs located within these defined genomic intervals were retrieved using the BioMart tool in the EnsemblPlants database, based on the *Triticum aestivum* reference genome annotation (https://plants.ensembl.org/biomart/martview). To prioritize CGs potentially involved in CS, two complementary approaches were employed. First, literature mining was conducted to identify genes whose encoded proteins have previously been reported to play potential roles in cold stress responses. Second, in silico gene expression analysis was performed using publicly available RNA-seq data (Li et al. 2015) accessed through the Wheat Expression Browser powered by WheatOmics (Ma et al. 2021; http://wheatomics.sdau.edu.cn/). Gene models exhibiting expression levels ≥ 5 transcripts per million (TPM) under cold stress compared with control conditions were considered biologically relevant and retained. Heatmaps illustrating gene expression patterns was generated using the ggplot2 package in R.

To prioritize CGs associated with yield and related traits, a two-step strategy was followed. First, literature mining was conducted to identify genes previously reported to be associated with yield-related traits. Second, gene network analysis of the remaining CGs was performed using the KnetMiner database (http://knetminer.com/Triticum_aestivum/). This analysis facilitated the identification of wheat genes functionally connected to yield-related traits through integrated biological knowledge networks.

## Results

### Phenotypic trait variation

**Fig. 2:**
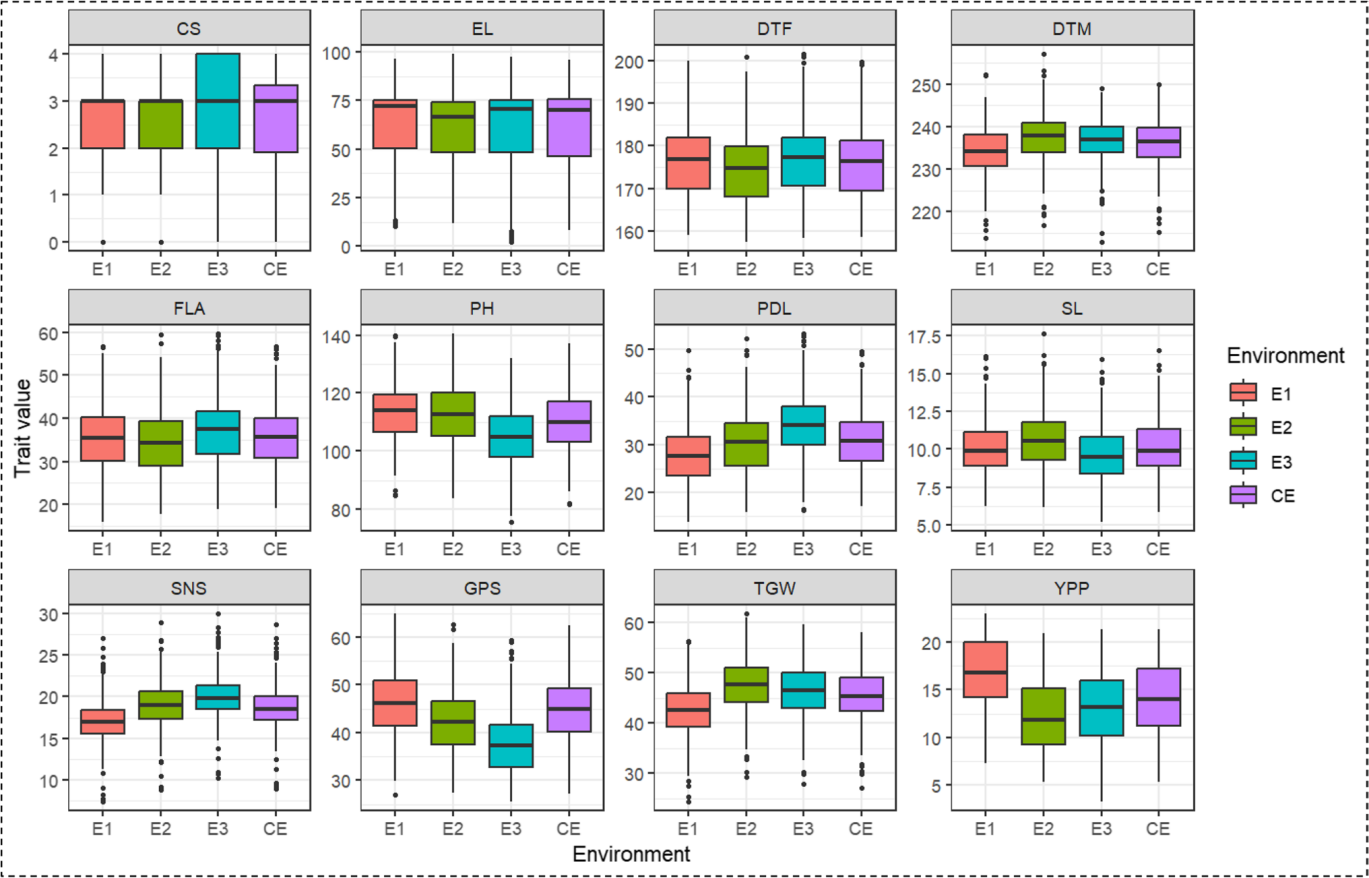
Box plot showing distributions for the studied traits across three environments (E1, E2, E3) and the combined environment (CE)

ANOVA based on ABD revealed highly significant (P < 0.01) variation among genotypes for all studied traits across E1, E2, E3 and CE under cold stress conditions (Table 2). Trait distributions based on check-adjusted means are presented in Fig. 2 for each environment using box plots. These distributions indicated significant phenotypic variability, confirming the quantitative nature of the measured traits. Most traits exhibited continuous distributions, although some traits deviated from normal distribution, exhibiting skewness (CS, EL and SL) or/and kurtosis (DTF and DTM). Descriptive statistics and genetic variability parameters for each trait are presented in Table 3.

**Table 2:** Analysis of variance for the studied traits across individual environments (E1, E2, E3) and combined environment (CE)

Source	Block (eliminating Treatments)	Block (ignoring Treatments)	Treatment (eliminating Blocks)	Treatment (ignoring Blocks)	Treatment: Test	Treatment: Check	Treatment: Test vs. Check	Treatment: Test and Test vs. Check	Residuals
Df	16	16	344	344	339	4	1	340	64
CS_E1_Mean.Sq	0.163 ns	1.471**	1.610**	1.671**	1.149**	46.206**	0.461 ns	1.086**	0.25
CS_E2_Mean.Sq	0.412 ns	3.745**	1.878**	2.029**	1.604**	35.988**	10.095**	1.477**	0.251
CS_E3_Mean.Sq	0.469 ns	2.619**	1.770**	1.870**	1.582**	25.571**	4.659**	1.490**	0.364
CS_CE_Mean.Sq	0.111 ns	2.311**	1.603**	1.705**	1.363**	30.087**	4.020**	1.268**	0.095
EL_E1_Mean.Sq	2.620 ns	593.244**	764.657**	792.128**	470.974**	28,096.973**	443.821**	443.100**	3.822
EL_E2_Mean.Sq	1.198 ns	1652.076**	729.684**	806.469**	471.257**	29,227.591**	758.925**	394.414**	1.259
EL_E3_Mean.Sq	1.267 ns	716.504**	772.598**	805.819**	524.015**	24,315.315**	2299.176**	495.625**	1.132
EL_CE_Mean.Sq	0.549 ns	842.546**	740.815**	779.978**	469.755**	27,007.379**	1036.097**	431.797**	0.735
DTF_E1_Mean.Sq	0.291 ns	91.295**	91.521**	95.754**	61.838**	2930.786**	253.076**	58.118**	0.615
DTF_E2_Mean.Sq	1.194 ns	94.343**	119.301**	123.634**	62.128**	2526.453**	11,362.626**	90.982**	1.003
DTF_E3_Mean.Sq	1.144 ns	86.552**	84.320**	88.292**	61.181**	2039.747**	1473.121**	61.315**	0.966
DTF_CE_Mean.Sq	0.444 ns	90.188**	92.467**	96.641**	61.234**	2402.581**	2876.007**	65.290**	0.268
DTM_E1_Mean.Sq	0.456 ns	50.740**	39.076**	41.415**	31.155**	868.718**	210.243**	29.315**	0.524
DTM_E2_Mean.Sq	0.400 ns	50.955**	65.532**	67.883**	32.665**	2876.806**	770.976**	32.458**	0.418
DTM_E3_Mean.Sq	0.574 ns	42.685**	85.280**	87.239**	35.871**	2086.218**	9504.841**	61.739**	0.555
DTM_CE_Mean.Sq	0.153 ns	46.992**	56.505**	58.684**	31.973**	1794.514**	2170.286**	36.058**	0.13
FLA_E1_Mean.Sq	0.918 ns	73.366**	80.056**	83.426**	61.416**	1965.362**	17.106**	57.876**	0.35
FLA_E2_Mean.Sq	0.541 ns	80.790**	70.064**	73.797**	63.670**	812.710**	551.117**	61.327**	0.518
FLA_E3_Mean.Sq	0.768 ns	106.966**	73.575**	78.514**	64.060**	1147.688**	701.905**	60.938**	0.584
FLA_CE_Mean.Sq	0.234 ns	55.010**	60.641**	63.189**	49.502**	1157.660**	325.265**	47.735**	0.168
PH_E1_Mean.Sq	0.489 ns	117.633**	110.424**	115.872**	94.226**	1948.472**	123.602**	88.799**	0.545
PH_E2_Mean.Sq	1.599 ns	153.224**	130.549**	137.601**	113.647**	2141.914**	240.851**	106.886**	1.249
PH_E3_Mean.Sq	0.296 ns	142.426**	148.083**	154.648**	105.921**	1814.857**	10,032.238**	128.474**	0.69
PH_CE_Mean.Sq	0.580 ns	135.718**	122.948**	129.233**	103.163**	1924.405**	1786.417**	101.754**	0.263
PDL_E1_Mean.Sq	15.558 ns	54.022**	57.053**	58.842**	41.499**	1141.288**	1608.563**	44.298**	16.334
PDL_E2_Mean.Sq	0.693 ns	55.329**	56.694**	59.235**	43.321**	1335.095**	350.721**	41.654**	0.53
PDL_E3_Mean.Sq	1.153 ns	62.789**	61.308**	64.161**	42.353**	824.637**	4415.222**	52.328**	0.659
PDL_CE_Mean.Sq	0.308 ns	55.389**	37.939**	40.454**	39.257**	150.425**	6.439**	36.616**	1.957
SL_E1_Mean.Sq	0.782 ns	4.021**	5.284**	5.435**	3.198**	75.606**	483.240**	4.457**	0.377
SL_E2_Mean.Sq	0.834 ns	3.742**	5.306**	5.441**	3.699**	133.359**	84.298**	3.799**	0.417
SL_E3_Mean.Sq	0.537 ns	3.367**	5.888**	6.020**	3.272**	142.780**	390.593**	4.278**	0.545
SL_CE_Mean.Sq	0.375 ns	3.554**	5.142**	5.290**	3.266**	106.144**	288.179**	3.954**	0.147
SNS_E1_Mean.Sq	0.633 ns	6.704**	10.915**	11.197**	6.807**	156.679**	917.448**	9.200**	0.395
SNS_E2_Mean.Sq	0.620 ns	7.685**	9.885**	10.214**	7.534**	212.576**	109.254**	7.501**	0.486
SNS_E3_Mean.Sq	0.452 ns	6.999**	9.996**	10.300**	6.999**	292.595**	0.205**	6.671**	0.561
SNS_CE_Mean.Sq	0.299 ns	7.005**	9.410**	9.722**	6.999**	195.809**	188.553**	7.217**	0.17
GPS_E1_Mean.Sq	0.515 ns	27.110**	67.948**	69.185**	55.330**	694.306**	2265.400**	60.579**	0.409
GPS_E2_Mean.Sq	0.691 ns	29.610**	67.098**	68.444**	56.434**	675.459**	1711.707**	59.941**	0.581
GPS_E3_Mean.Sq	0.702 ns	35.121**	77.619**	79.220**	61.282**	867.475**	3007.203**	68.327**	0.987
GPS_CE_Mean.Sq	0.302 ns	29.999**	63.208**	64.590**	56.827**	672.938**	262.597**	56.035**	0.197
TGW_E1_Mean.Sq	0.428 ns	54.229**	35.422**	37.925**	27.158**	349.063**	2443.382**	31.732**	0.522
TGW_E2_Mean.Sq	0.254 ns	58.912**	35.416**	38.098**	27.363**	956.171**	5.054**	24.584**	0.525
TGW_E3_Mean.Sq	0.781 ns	67.538**	33.084**	36.189**	30.758**	503.968**	6.343**	27.545**	0.836
TGW_CE_Mean.Sq	0.411 ns	59.733**	31.189**	33.948**	27.299**	537.283**	274.465**	25.235**	0.23
YPP_E1_Mean.Sq	0.692 ns	28.365**	15.207**	16.494**	13.748**	251.217**	8.532**	12.430**	0.402
YPP_E2_Mean.Sq	0.506 ns	23.865**	15.407**	16.493**	12.670**	236.074**	434.093**	12.811**	0.514
YPP_E3_Mean.Sq	0.584 ns	29.718**	16.750**	18.105**	14.851**	184.796**	454.455**	14.773**	0.469
YPP_CE_Mean.Sq	0.249 ns	26.571**	15.006**	16.231**	13.279**	214.017**	225.739**	12.665**	0.158

CS: Cold stress tolerance; EL: Electrolyte leakage; DTF: Days to flowering; DTM: Days to maturity; FLA: Flag leaf area; PH: Plant height; PDL: Peduncle length; SL: Spike length; SNS: Number of spikelets per spikes; TGW: Thousand grain weight; GPS: Grain per spikes; YPP: Yield per plant; Mean.Sq: Mean sum of square; * p < 0.05, ** p < 0.01, ns = non-significant

**Table 3:** Summary of overall descriptive statistics, variance components, and genetic parameters of synthetic-derived nested introgression libraries (S-NILibs) across three environments (E1, E2, E3) and the combined environment (CE)

Trait	Count	Min	Max	Mean	Std.Error	Std.Deviation	PV	GV	EV
CS_E1	345	0.00	4.00	2.44	0.06	1.10	1.15	0.90	0.25
CS_E2	345	0.00	4.00	2.61	0.07	1.25	1.60	1.35	0.25
CS_E3	345	0.00	4.00	2.73	0.07	1.27	1.58	1.22	0.36
CS_CE	345	0.00	4.00	2.58	0.06	1.17	1.36	1.27	0.10
EL_E1	345	9.71	96.44	62.16	1.19	22.09	470.97	467.15	3.82
EL_E2	345	13.34	99.61	63.36	1.18	21.96	471.26	470.00	1.26
EL_E3	345	5.26	98.47	62.91	1.24	23.05	524.02	522.88	1.13
EL_CE	345	10.03	96.38	62.81	1.18	21.94	469.75	469.02	0.74
DTF_E1	345	159.34	200.29	176.19	0.43	7.98	61.84	61.22	0.61
DTF_E2	345	157.97	200.94	174.60	0.44	8.09	62.13	61.13	1.00
DTF_E3	345	158.77	201.47	176.95	0.43	7.90	61.18	60.22	0.97
DTF_CE	345	158.81	199.59	175.91	0.43	7.93	61.23	60.97	0.27
DTM_E1	345	214.21	252.38	234.10	0.30	5.60	31.15	30.63	0.52
DTM_E2	345	217.37	256.94	237.08	0.31	5.85	32.67	32.25	0.42
DTM_E3	345	212.51	258.82	236.48	0.34	6.26	35.87	35.32	0.56
DTM_CE	345	215.40	251.82	235.89	0.31	5.77	31.97	31.84	0.13
FLA_E1	345	15.75	56.71	35.35	0.42	7.89	61.42	61.07	0.35
FLA_E2	345	18.44	59.66	34.62	0.43	7.94	63.67	63.15	0.52
FLA_E3	345	18.17	59.68	37.15	0.43	8.05	64.06	63.48	0.58
FLA_CE	345	19.31	56.69	35.71	0.38	7.04	49.50	49.33	0.17
PH_E1	345	84.32	140.42	113.31	0.52	9.73	94.23	93.68	0.54
PH_E2	345	84.09	140.33	112.83	0.57	10.64	113.65	112.40	1.25
PH_E3	345	75.46	131.69	105.26	0.56	10.38	105.92	105.23	0.69
PH_CE	345	81.47	137.03	110.47	0.55	10.16	103.16	102.90	0.26
PDL_E1	345	13.16	49.01	27.95	0.36	6.63	41.50	25.16	16.33
PDL_E2	345	16.20	52.19	30.67	0.35	6.57	43.32	42.79	0.53
PDL_E3	345	15.32	53.96	34.10	0.36	6.62	42.35	41.69	0.66
PDL_CE	345	17.15	49.50	30.90	0.33	6.20	39.26	37.30	1.96
SL_E1	345	5.89	16.62	10.20	0.10	1.85	3.20	2.82	0.38
SL_E2	345	6.28	18.26	10.73	0.10	1.95	3.70	3.28	0.42
SL_E3	345	5.67	16.01	9.76	0.10	1.87	3.27	2.73	0.54
SL_CE	345	6.20	16.82	10.23	0.10	1.84	3.27	3.12	0.15
SNS_E1	345	7.62	27.20	17.12	0.14	2.67	6.81	6.41	0.39
SNS_E2	345	8.76	28.96	19.08	0.15	2.76	7.53	7.05	0.49
SNS_E3	345	10.01	29.28	20.01	0.14	2.67	7.00	6.44	0.56
SNS_CE	345	8.90	28.48	18.74	0.14	2.66	7.00	6.83	0.17
GPS_E1	345	26.93	65.21	46.20	0.40	7.48	55.33	54.92	0.41
GPS_E2	345	27.69	62.36	42.35	0.40	7.51	56.43	55.85	0.58
GPS_E3	345	25.62	59.70	37.34	0.42	7.86	61.28	60.30	0.99
GPS_CE	345	27.11	59.23	41.96	0.41	7.53	56.83	56.63	0.20
TGW_E1	345	24.60	56.28	42.61	0.28	5.25	27.16	26.64	0.52
TGW_E2	345	30.18	62.65	47.71	0.28	5.20	27.36	26.84	0.52
TGW_E3	345	28.05	59.18	46.40	0.30	5.49	30.76	29.92	0.84
TGW_CE	345	27.61	57.79	45.58	0.28	5.19	27.30	27.07	0.23
YPP_E1	345	7.19	23.32	16.97	0.20	3.75	13.75	13.35	0.40
YPP_E2	345	5.95	20.68	12.28	0.19	3.55	12.67	12.16	0.51
YPP_E3	345	3.02	21.65	13.17	0.21	3.84	14.85	14.38	0.47
YPP_CE	345	5.57	21.40	14.14	0.20	3.64	13.28	13.12	0.16

Trait	GCV	PCV	ECV	CV	Skewness	Kurtosis	h^2^	GA	GAM
CS_E1	38.86	43.93	20.47	20.66	−0.89	2.81	78.28	1.73	70.93
CS_E2	44.64	48.60	19.22	19.87	−0.84	2.56	84.37	2.20	84.60
CS_E3	40.48	46.14	22.14	22.62	−0.63	2.34	76.98	2.00	73.27
CS_CE	43.59	45.20	11.95	12.17	−0.92	2.58	93.01	2.24	86.72
EL_E1	34.77	34.91	3.15	3.17	−0.99	2.83	99.19	44.41	71.44
EL_E2	34.22	34.26	1.77	1.79	−0.69	2.57	99.73	44.66	70.49
EL_E3	36.35	36.39	1.69	1.72	−1.15	3.18	99.78	47.12	74.90
EL_CE	34.48	34.51	1.37	1.38	−1.03	2.90	99.84	44.64	71.07
DTF_E1	4.44	4.46	0.44	0.44	0.25	2.86	99.01	16.06	9.12
DTF_E2	4.48	4.51	0.57	0.57	0.38	3.15	98.39	16.00	9.16
DTF_E3	4.39	4.42	0.56	0.55	0.29	3.06	98.42	15.88	8.98
DTF_CE	4.44	4.45	0.29	0.29	0.29	2.96	99.56	16.07	9.14
DTM_E1	2.36	2.38	0.31	0.31	−0.38	3.69	98.32	11.32	4.84
DTM_E2	2.40	2.41	0.27	0.27	−0.30	3.67	98.72	11.64	4.91
DTM_E3	2.51	2.53	0.32	0.31	−0.34	4.42	98.45	12.16	5.14
DTM_CE	2.39	2.40	0.15	0.15	−0.40	3.53	99.59	11.62	4.93
FLA_E1	22.10	22.17	1.67	1.67	0.14	2.82	99.43	16.08	45.47
FLA_E2	22.95	23.05	2.08	2.05	0.32	2.85	99.19	16.33	47.16
FLA_E3	21.45	21.55	2.06	2.03	0.13	2.90	99.09	16.36	44.05
FLA_CE	19.67	19.70	1.15	1.13	0.24	2.97	99.66	14.47	40.51
PH_E1	8.54	8.57	0.65	0.65	0.02	2.97	99.42	19.91	17.57
PH_E2	9.40	9.45	0.99	0.99	0.08	2.69	98.90	21.75	19.28
PH_E3	9.75	9.78	0.79	0.77	0.08	2.84	99.35	21.09	20.04
PH_CE	9.18	9.19	0.46	0.46	0.07	2.80	99.75	20.90	18.92
PDL_E1	17.95	23.05	14.46	14.01	0.44	3.04	60.64	8.06	28.83
PDL_E2	21.33	21.46	2.37	2.34	0.37	2.98	98.78	13.41	43.74
PDL_E3	18.94	19.08	2.38	2.49	0.14	3.23	98.44	13.22	38.76
PDL_CE	19.76	20.27	4.53	4.53	0.35	3.01	95.02	12.28	39.74
SL_E1	16.46	17.53	6.02	5.74	0.66	3.43	88.22	3.25	31.90
SL_E2	16.89	17.93	6.02	5.91	0.46	3.28	88.71	3.52	32.82
SL_E3	16.92	18.53	7.56	7.23	0.65	3.36	83.35	3.11	31.87
SL_CE	17.26	17.67	3.75	3.62	0.63	3.39	95.49	3.56	34.80
SNS_E1	14.79	15.24	3.67	3.53	0.22	4.79	94.20	5.07	29.62
SNS_E2	13.91	14.38	3.65	3.61	0.07	4.23	93.54	5.30	27.76
SNS_E3	12.68	13.22	3.74	3.74	0.10	4.52	91.98	5.02	25.08
SNS_CE	13.95	14.12	2.20	2.16	0.13	4.58	97.57	5.33	28.42
GPS_E1	16.04	16.10	1.38	1.42	−0.22	4.40	99.26	15.23	32.97
GPS_E2	17.65	17.74	1.80	1.76	−0.31	4.64	98.97	15.34	36.22
GPS_E3	20.80	20.97	2.66	2.58	−0.03	4.43	98.39	15.89	42.56
GPS_CE	17.93	17.96	1.06	1.05	−0.25	4.42	99.65	15.50	36.93
TGW_E1	12.11	12.23	1.70	1.65	−0.03	3.55	98.08	10.54	24.74
TGW_E2	10.86	10.96	1.52	1.52	0.04	3.49	98.08	10.58	22.18
TGW_E3	11.79	11.95	1.97	1.97	−0.25	3.35	97.28	11.13	23.99
TGW_CE	11.42	11.46	1.05	1.04	−0.12	3.34	99.16	10.69	23.45
YPP_E1	21.53	21.85	3.74	3.72	−0.23	2.36	97.08	7.43	43.76
YPP_E2	28.40	28.99	5.84	5.63	0.16	1.99	95.94	7.05	57.38
YPP_E3	28.79	29.26	5.20	5.02	−0.10	2.25	96.84	7.70	58.46
YPP_CE	25.62	25.77	2.81	2.75	−0.05	2.07	98.81	7.43	52.53

CS: Cold stress; EL: Electrolyte leakage; DTF: Days to flowering; DTM: Days to maturity; FLA: Flag leaf area; PH: Plant height; PDL: Peduncle length; SL: Spike length; SNS: Number of spikelets per spikes; TGW: Thousand grain weight; GPS: Grain per spikes; YPP: Yield per plant; PV: Phenotypic variance; GV: Genotypic variance; EV: Environmental variance; PCV: Phenotypic coefficient of variance; GCV: Genotypic coefficient of variance; EV: Environmental coefficient of variance; h2: Broad sense heritability; GA: Genetic advance; GAM: Genetic advance mean

The CE-based phenotypic ranges for the studied traits were as follows: CS (0.00–4.00), EL (10.03–96.38), DTF (158.81–199.59), DTM (215.40–251.82), FLA (19.31–56.69 cm^2^), PH (81.47–137.03 cm), PDL (17.15–49.50 cm), SL (6.20–16.82 cm), SNS (8.90–28.48), GPS (27.11–59.23), YPP (5.57–21.40 g), and TGW (27.61–57.79 g). Phenotypic data of S-NILibs and five checks across environments are provided in (Table S1-S4).

Based on CS and the performance of key traits affecting the final yield, several transgressive segregants were identified. Across environments, 34 S-NILibs outperformed the checks for CS, 8 for EL, 32 for DTF, 33 for DTM, 19 for GPS, 11 for SNS, 9 for TGW and 19 for YPP. Notably, accessions PN 338, PN 279, PN 734, PN 551, and PN 687simultaneously exhibited cold stress resistance and high yield potential across all environments, making them promising candidates for breeding programs under temperate conditions.

### Phenotypic correlations and principal component analysis (PCA)

**Fig. 3:**
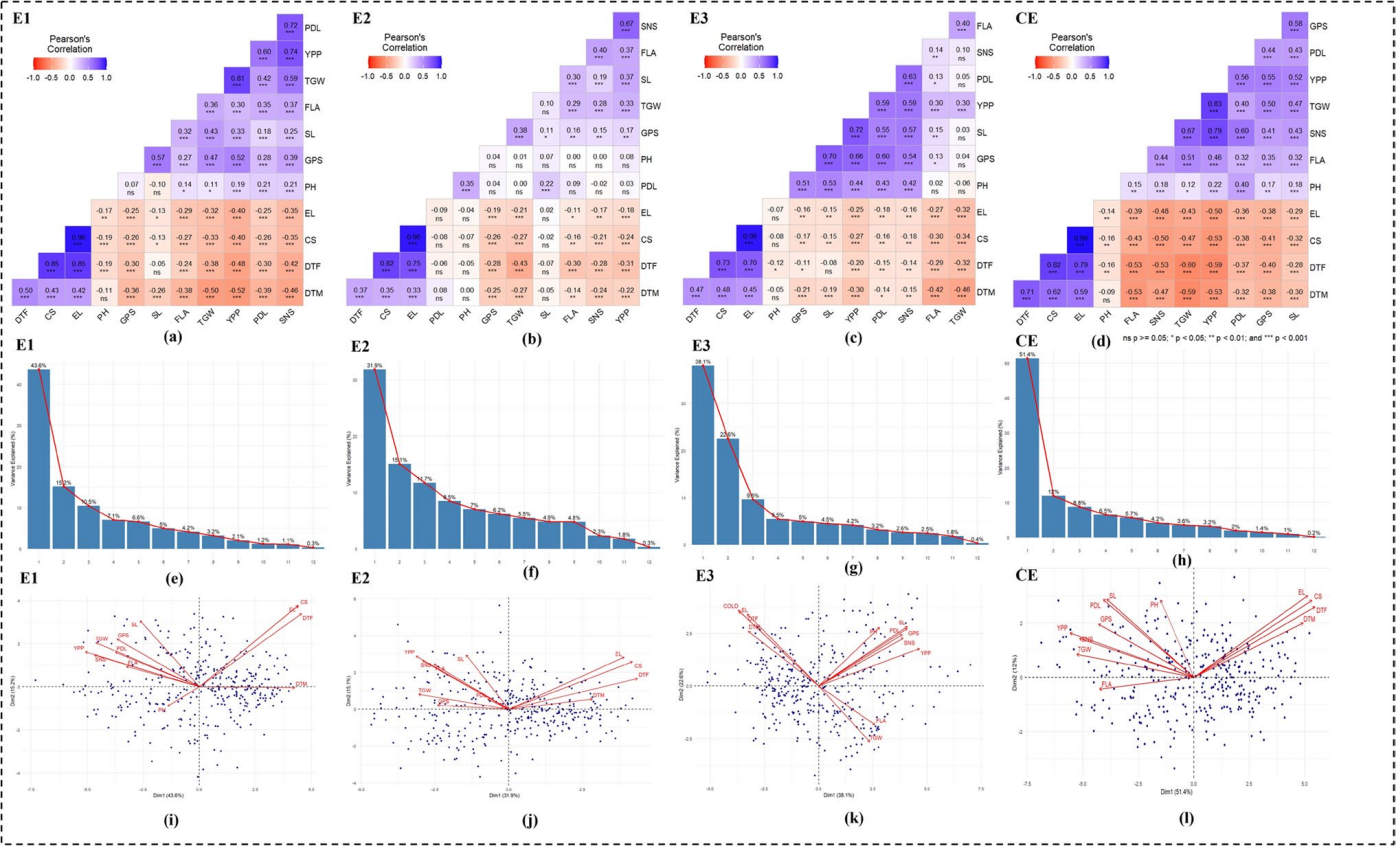
Pearson’s correlation analysis, scree plots, and principal component analysis (PCA) across individual environments (E1, E2, E3) and the combined environment (CE). (**a-d**) Pearson’s correlation heatmaps showing relationships among the studied traits. (**e–h**) Scree plots depicting the proportion of total phenotypic variance explained by each principal component. (**i-l**) PCA biplots illustrating the relationships among traits and genotypes

PCA biplots, scree plots, and correlation matrices for all environments are presented in Fig. 3. Based on CE performance, the first two principal components explained 63.43% of the total variability (Table S5). Significant positive correlations (P < 0.001) were observed between YPP and several yield-related traits, such as TGW (0.83), SNS (0.79), PDL (0.56), GPS (0.55), SL (0.52), FLA (0.46) and PH (0.22). Among the cold tolerance traits, high CS value showed significant negative correlation with various yield component traits, including YPP (− 0.53)*,* SNS (−0.50), TGW (− 0.47)*,* FLA (− 0.43), GPS (− 0.41), PDL (− 0.38), SL (− 0.32) and PH (− 0.16) at P < 0.001. Conversely, CS was positively correlated with EL (0.98), DTF (0.82), and DTM (0.62).

### Population structure and kinship analysis

Population structure of the 340 S-NILs was analysed using 9,348 filtered SNP markers. STRUCTURE analysis revealed a clear peak in ΔK at K = 2, indicating two major sub-populations corresponding to the two SHW donors. Consistently, PCA and neighbor-joining tree analysis classified the panel into two distinct clusters. The kinship heatmap further supported these results, showing strong relatedness within sub-groups and clear genetic differentiation between them. Details on marker distribution, population structure inference and kinship estimation were reported previously by Kaur et al. (2025).

### GWAS and haplotype analysis

**Fig. 4:**
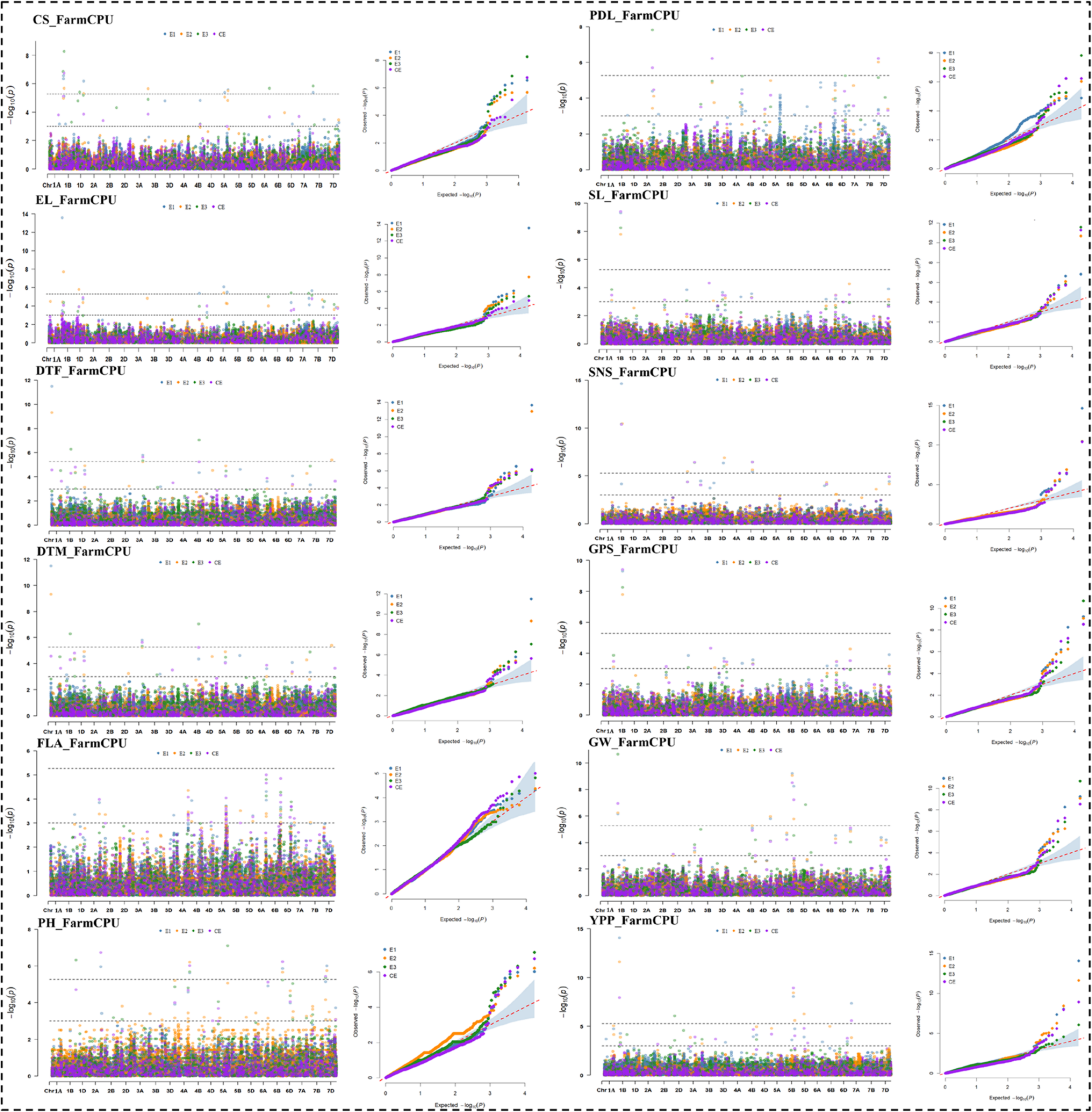
Manhattan and Quantile–quantile (Q–Q) plots showing GWAS results for cold stress tolerance (CS), electrolyte leakage (EL) and days to flowering (DTF) days to maturity (DTM) and flag leaf Area (FLA), plant height (PH) and peduncle length (PDL), spike length (SL) and number of spikelets per spikes (SNS), grain per spike (GPS) and thousand grain weight (TGW) and yield per plant (YPP) across environments (E1, E2, E3 and CE), using the FarmCPU model. The horizontal lines indicate the significance thresholds corresponding to − log₁₀P ≥ 3.00 and the Bonferroni-corrected genome-wide threshold of − log₁₀(P) ≥ 5.27

Significant MTAs were identified using two thresholds: (i) − log₁₀(P) ≥ 3.0, and (ii) a Bonferroni-corrected threshold (− log₁₀(P) ≥ 5.27). Based on these criteria, several significant along with stable MTAs were detected for the studied traits (Tables S6–S17). A combined set of Manhattan and Q–Q plots analyzed using the FarmCPU and BLINK models, are presented in Fig. 4 and Fig. S2. Subsequently, LD blocks were constructed across the genome using SNP data. In total, 32 major LD blocks spanning genomic regions around stable MTAs were identified and used for haplotype construction. The overall haplotype effects were assessed using ANOVA, and significant differences were detected for most LD blocks. However, no significant haplotype effect was observed for the fourth LD block on chromosome 6 A for DTF, the third LD block on chromosome 5 A for GPS, and the first LD block on chromosome 1 A for GPS (p > 0.05) (Tables S23–S54). Pairwise comparisons among haplotypes were performed using EMMs derived from the linear model, and multiple testing was controlled using the Benjamini–Hochberg FDR correction.

#### Cold tolerance score (CS)

GWAS using FarmCPU and BLINK identified 12 and 29 MTAs in E1, 9 and 19 in E2, 15 and 15 in E3, and 10 and 12 in the CE dataset, respectively (Table S6). Among these, 14 stable MTAs were detected across seven chromosomes: 1 A (2), 1B (3), 1D (4), 3B (1), 4B (1), 5 A (2), and 7B (1), with − log₁₀(P) values ranging from 3.00 to 8.26 (Table 4). Of these, seven MTAs exceeded the Bonferroni-corrected threshold.

**Table 4:** Summary of stable MTAs, pleiotropic MTAs and previously reported colocalised MTAs identified for the studied traits

S.no	Traits	SNP	Chr	Pos	Model	Env	-Log_10_ P.value range	MAF	Effect range	PVE range %	Pleiotropic	Previously reported MTAs/QTLs/MQTLs	Previously reported MTAs/QTLs/MQTLs position	References
**1**	**CS**	AX-94670671	1A	506.68	BLINK	E2, E3, CE	3.22—3.58	0.25	(−0.29)—(−0.27)	NA	––––––––-	wsnp_Ku_c23012_32893918	516.37	Zhang et al. 2023b
**2**		AX-95246613	1A	512.25	BLINK	E1, E2, E3, CE	3.43—3.97	0.22	(−0.36)—(−0.27)	NA	––––––––-	wsnp_Ku_c23012_32893918	516.37	Zhang et al. 2023b
**3**		AX-95172504*	1B	69.61	FarmCPU	E1, E2, CE	4.98—6.55	0.11	0.42—0.44	21.90	––––––––-	––––––––-	––––––––-	Novel
**4**		AX-95196667*	1B	117.85	FarmCPU	E1, E2, E3, CE	4.74—8.26	0.48	(−1.56)—(−0.92)	2.56—44.65	EL, DTM	*qCT1B.2*	102.1–140.9	Pang et al. 2021
					BLINK									
**5**		AX-94708814	1B	56.17	BLINK	E1, E2, E3	3.00—4.30	0.45	0.32—0.33	NA	EL	S1B_56585121; AX-111096324	56.59; 49.04	Rojas‐Gutierrez et al. 2022; Zhang et al. 2023b
**6**		AX-94453107	1D	407.92	BLINK	E2, E3, CE	3.08—3.74	0.26	(−0.30)—(−0.26)	NA	––––––––-	Kukri_c18413_496	407.87	Zhang et al. 2023b
**7**		AX-94471137*	1D	414.51	FarmCPU	E1, E2, CE	3.27—6.19	0.22	(−0.34)—(−0.30)	3.77—4.44	EL, DTF, DTM	wsnp_Ex_rep_c66423_64641115	415.42	Zhang et al. 2023b
					BLINK	E2, E3, CE								
**8**		AX-94503893	1D	408.25	BLINK	E2, E3	3.18—3.80	0.25	(−0.31)—(−0.28)	NA	DTF	Kukri_c18413_496	407.87	Zhang et al. 2023b
**9**		AX-94403925	1D	408.21	BLINK	E2, E3, CE	3.14—3.82	0.25	(−0.31)—(−0.27)	NA	––––––––-	Kukri_c18413_496	407.87	Zhang et al. 2023b
**10**		AX-94754126*	3B	9.04	FarmCPU	E2, E3, CE	3.10—5.64	0.18	0.27—0.35	5.41	––––––––-	––––––––-	––––––––-	Novel
					BLINK									
**11**		AX-95094133	4B	416.31	FarmCPU	E1, E3, CE	3.13—4.81	0.48	(−0.75)—(−0.71)	NA	EL, DTM, SL	AX-111719562	428.21	Zhang et al. 2023b
**12**		AX-95015035*	5A	399.37	FarmCPU	E1, E2	5.05—5.94	0.29	0.22—0.94	10.65—16.49	EL	Kukri_rep_c110025_90; AX-111772046	398.16; 398.04	Zhao et al. 2020a, ; Zhang et al. 2023b
					BLINK									
**13**		AX-95228006*	5A	590.69	FarmCPU	E1, E2	5.03—5.58	0.32	0.26—0.88	5.89—10.58	EL	wsnp_Ex_c17763_26513562	591.32	Zhang et al. 2023b
					BLINK							BS00031117_51	588.38	Zhang et al. 2023b
												BS00075959_51	588.74	Zhao et al. 2020a,
**14**		AX-94426964*	7B	27.35	FarmCPU	E1, E3	5.37—5.84	0.23	0.26—0.35	2.90—5.53	EL	––––––––-	––––––––-	
**15**	**EL**	AX-94708814	1B	56.17	BLINK	E1, E3, CE	3.24—4.57	0.45	5.70—6.89	NA	CS	––––––––-	––––––––-	
**16**		AX-95196667*	1B	117.85	FarmCPU	E2, E3, CE	4.07—7.72	0.48	(−25.65)—(−16.78)	32.91—46.04	CS	––––––––-	––––––––-	
					BLINK							––––––––-	––––––––-	
**17**		AX-94471137	1D	414.51	FarmCPU	E1, E2, CE	4.36—4.91	0.22	0.03—0.07	NA	CS, DTF, DTM	––––––––-	––––––––-	
**18**		AX-94645057	1D	127.93	BLINK	E1, E3	3.25—3.32	0.1	(−19.80)—(−18.41)	NA	––––––––-	––––––––-	––––––––-	
**19**		AX-94695356*	1D	184.48	FarmCPU	E2, E3, CE	3.90—5.77	0.08	0.01—0.23	9.61	––––––––-	––––––––-	––––––––-	
**20**		AX-95094133*	4B	416.31	FarmCPU	E1, E3	3.27—5.36	0.48	(−16.32)—(−14.28)	34.53	CS	––––––––-	––––––––-	
					BLINK	E1, E2, E3, CE								
**21**		AX-95015035*	5A	399.37	FarmCPU	E1, E2	5.43—6.09	0.29	0.28—1.00	7.27—11.84	CS	––––––––-	––––––––-	
**22**		AX-95228006*	5A	590.69	FarmCPU	E1, E2	4.25—5.50	0.32	0.76—1.36	3.96—6.93	CS	––––––––-	––––––––-	
**23**		AX-94989647	6A	581.98	FarmCPU	E2, E3	4.00—4.99	0.34	(−5.76)—(−4.91)	NA	––––––––-	––––––––-	––––––––-	
**24**		AX-94998023*	6D	307.11	FarmCPU	E1, E3, CE	3.48—5.41	0.08	(−13.26)—(−9.58)	5.78	––––––––-	––––––––-	––––––––-	
**25**		AX-94426964*	7B	27.35	FarmCPU	E1, E3, CE	3.12—5.68	0.23	4.60—5.50	4.40	CS	––––––––-	––––––––-	
					BLINK	E1, E3						––––––––-	––––––––-	
**26**		AX-94402434	7D	415.39	FarmCPU	E2, E3	3.65—4.15	0.34	4.95—5.43	NA		––––––––-	––––––––-	
**27**	**DTF**	AX-95205762	1A	552.44	FarmCPU	E1, E2	4.35—5.26	0.06	2.50—3.21	NA	––––––––-	*qHD1A.1; AX-95113161*	543.25; 560.46	Pang et al. 2020; Nawade et al. 2025
**28**		AX-95236907	1A	508.63	BLINK	E1, E2, CE	3.01—3.24	0.24	(−1.70)—(−1.63)	NA	––––––––-	––––––––-	––––––––-	Novel
**29**		AX-94911518*	1A	8.92	FarmCPU	E1, E2, E3, CE	3.51—13.69	0.09	3.57—5.93	25.71—29.14	DTM	––––––––-	––––––––-	Novel
					BLINK	E1, E2, CE			3.54—3.61			––––––––-	––––––––-	Novel
**30**		AX-95159552	1B	681.28	FarmCPU	E1, E2, E3, CE	4.01—4.40	0.33	(−1.97)—(−1.66)	NA	DTM	––––––––-	––––––––-	Novel
**31**		AX-94732589*	1D	444.86	FarmCPU	E1, E2, E3, CE	5.65—6.14	0.09	(−3.18)—(−2.92)	4.85—7.50	––––––––-	––––––––-	––––––––-	Novel
**32**		AX-94742606	1D	412.16	BLINK	E1, E2	3.01—3.19	0.25	(−1.72)—(−1.66)	NA	––––––––-	S1D_416730006; S1D_417084381	416.73; 417.08	Jamil et al. 2019
**33**		AX-95014770	1D	410.51	BLINK	E1, E2, CE	3.04—3.30	0.24	(−1.75)—(−1.67)	NA	––––––––-			
**34**		AX-95148840	1D	408.20	BLINK	E1, E2,	3.14—3.37	0.26	(−1.72)—(−1.64)	NA	––––––––-			
**35**		AX-94471137	1D	414.51	BLINK	E1, E2, CE	3.08—3.30	0.22	(−1.96)—(−1.88)	NA	CS, EL, DTM			
**36**		AX-94503893*	1D	408.25	FarmCPU	E2, E3, CE	3.37—6.93	0.25	(−1.97)—(−1.13)	4.44—5.45	CS			
					BLINK	E1, E2, E3, CE								
**37**		AX-94516050	1D	412.09	BLINK	E1, E2, CE	3.03—3.22	0.25	(−1.77)—(−1.66)	NA	––––––––-			
**38**		AX-94432771	1D	408.25	BLINK	E1, E2, CE	3.00—3.23	0.25	(−1.70)—(−1.63)	NA	––––––––-			
**39**		AX-94868122	1D	408.53	FarmCPU	E2, E3, CE	3.57—3.61	0.21	3.90—4.00	NA	––––––––-			
**40**		AX-94466657*	3A	564.34	FarmCPU	E1, E2, E3, CE	3.19—6.53	0.26	(−1.92)—(−1.49)	2.83	DTM	AX-111630734	558.01	Li et al. 2019a, b
					BLINK	E1, E2								
**41**		AX-95251140	5A	535.73	FarmCPU	E1, E2, E3, CE	3.56—5.89	0.18	(−2.45)—(−1.74)	NA	DTM	AX-94935237	540.29	Nawade et al. 2025
**42**		AX-94809955	6A	0.63	FarmCPU	E1, E3	3.11—4.53	0.09	1.91—2.81	NA	––––––––-	––––––––-	––––––––-	Novel
**43**		AX-94989647	6A	581.98	FarmCPU	E1, E2, E3, CE	3.21—4.61	0.34	(−1.75)—(−1.42)	NA	DTM	*qHD6A.2*	572.02	Pang et al. 2020
**44**		AX-94554795	7A	717.75	FarmCPU	E2, E3	4.99—5.13	0.32	(−1.61)—(−1.58)	NA	––––––––-	*qHD7A.8*	717.18	Pang et al. 2020
**45**	**DTM**	AX-94911518*	1A	8.92	FarmCPU	E1, E2, CE	4.57—11.50	0.09	2.60—3.99	27.49—31.43	DTF	AX-95162217; AX-95652739	6.27; 7.64	Sheoran et al. 2022; Gaur et al. 2024
**46**		AX-95159552	1B	681.28	FarmCPU	E2, E3	4.35—4.81	0.33	(−1.53)—(−1.42)	NA	DTF	––––––––-	––––––––-	Novel
**47**		AX-95196667	1B	117.85	BLINK	E2, E3, CE	4.13—4.45	0.48	(−4.73)—(5.04)	NA	––––––––-	––––––––-	––––––––-	
**48**		AX-94471137	1D	414.51	FarmCPU	E2, CE	4.22—4.92	0.22	(−1.17)—(- 1.06)	NA	CS, EL, DTF	––––––––-	––––––––-	
**49**		AX-94466657*	3A	564.34	FarmCPU	E1, E2, E3, CE	5.25—5.80	0.26	(−1.36)—(−1.22)	3.22—3.53	DTF	––––––––-	––––––––-	
**50**		AX-95094133*	4B	416.31	FarmCPU	E1, E3, CE	4.36—7.05	0.48	(−4.67)—(−3.31)	38.00	SL, SNS, GPS, GW	––––––––-	––––––––-	
**51**		AX-95251140	5A	535.73	FarmCPU	E1, E2, CE	4.05—4.91	0.18	(−1.60)—(−1.35)	NA	DTF	AX-95257620	533.29	Nawade et al. 2025
**52**		AX-94989647	6A	581.98	FarmCPU	E1, E2, CE	3.01—3.89	0.34	(−1.21)—(−0.99)	NA	DTF	––––––––-	––––––––-	Novel
**53**		AX-94589700	6D	469.37	FarmCPU	E1, CE	3.37—4.10	0.08	1.52—1.73	NA	––––––––-	AX-111919223	466.89—472.91	Ding et al. 2023
**54**		AX-94638774	7B	33.81	FarmCPU	E1, E3	3.35—4.90	0.26	1.01—1.34	NA	––––––––-	––––––––-	––––––––-	Novel
**55**	**FLA**	AX-94517884	1B	686.14	BLINK	E1, E2	3.03—3.36	0.49	(−11.62)—(−9.66)	NA	PDL	––––––––-	––––––––-	Novel
**56**		AX-94486441	2A	702.11	FarmCPU	E1,E2, CE	3.39—3.98	0.34	(−1.78)—(−1.59)	NA	PDL	AX_109951135	716.33	Chen et al. 2021
**57**		AX-95630121*	4A	708.68	FarmCPU	E1, E2	3.01—7.07	0.24	(−2.52)—(−1.94)	7.03—9.40	PH, PDL	AX_94484676	709.66	
					BLINK	E1, E2, CE								
**58**		AX-95651615	4B	33.35	FarmCPU	E1, E2	3.07—3.94	0.49	(−10.97)—(−7.61)	NA	––––––––-	––––––––-	––––––––-	Novel
					BLINK	E1, E2					––––––––-	––––––––-	––––––––-	
**59**		AX-94934730*	5A	608.62	FarmCPU	E1, E2, CE	3.68—5.43	0.31	(−1.94)—(−1.59)	6.16—8.22	PH	*MQTL-5A.6 (Xgwm6b-Xcfd39)*	601.39–661.14	Kong et al. 2023
					BLINK	E1, E2, CE								
**60**		AX-94517891	5A	609.27	FarmCPU	E1, E2	3.47—3.64	0.30	(−1.94)—(−1.68)	NA	––––––––-		601.39–661.14	Kong et al. 2023
**61**		AX-94552678	5A	613.54	FarmCPU	E1, E2, CE	3.08—3.17	0.30	(−1.79)—(−1.54)	NA	––––––––-	*MQTL-5A.7 (BS00026916_51-wsnp_Ex_c1481_2831499)*	613.58–653.85	Kong et al. 2023
**62**		AX-94725002	5A	608.34	FarmCPU	E1, E2, CE	3.06—3.39	0.33	(−1.80)—(−1.57)	NA	––––––––-	––––––––-	––––––––-	Novel
**63**		AX-94391981	5A	609.21	FarmCPU	E1, E2, CE	3.37—3.55	0.31	(−1.86)—(−1.62)	NA	––––––––-	––––––––-	––––––––-	
**64**		AX-94419595	5A	609.25	FarmCPU	E1, E2, CE	3.20—3.34	0.31	(−1.03)—(−1.59)	NA	––––––––-	––––––––-	––––––––-	
**65**		AX-94987657	5D	495.03	FarmCPU	E1, E2	3.08—3.31	0.30	(−1.76)—(−1.56)	NA	––––––––-	D_GBF1XID01CVZMX_132	483.52	Gao et al. 2021
**66**		AX-94768049	6A	598.64	FarmCPU	E1, E2, E3, CE	3.40—4.66	0.47	(−2.35)—(−2.02)	NA	––––––––-	––––––––-	––––––––-	Novel
					BLINK	E1, E2, E3					––––––––-	––––––––-	––––––––-	
**67**		AX-94779816*	6A	598.64	FarmCPU	E1, E2	3.14—6.22	0.48	(−2.48)—(−1.88)	15.43	––––––––-	––––––––-	––––––––-	
					BLINK	E1, E2, E3, CE					––––––––-	––––––––-	––––––––-	
**68**		AX-94422554	6B	686.69	FarmCPU	E1, E3	3.23—3.87	0.46	(−2.13)—(−1.95)	NA	––––––––-	*MQTL-6B.5 (Xgwm570-BS00034554_51)*	653.20–688.31	Kong et al. 2023
					BLINK						––––––––-			
**69**		AX-94847283	6B	687.73	FarmCPU	E1, E2, E3, CE	3.50—4.85	0.47	(−2.42)—(−1.99)	NA	PDL	––––––––-	––––––––-	Novel
					BLINK	E1, E2						––––––––-	––––––––-	
**70**		AX-95630592	6B	686.80	FarmCPU	E1, E2	3.05—4.17	0.49	(−2.21)—(−1.80)	NA	PH, PDL	––––––––-	––––––––-	
					BLINK	E1, E2, E3						––––––––-	––––––––-	
**71**		AX-95217910	6D	450.21	FarmCPU	E1, E3, CE	3.01—3.69	0.48	(−2.11)—(−2.04)	NA	YPP	––––––––-	––––––––-	
					BLINK	E1, E3						––––––––-	––––––––-	
**72**	**PH**	AX-94633968	2D	107.40	FarmCPU	E2, E3	3.10—3.81	0.49	11.99—14.26	NA	––––––––-	––––––––-	––––––––-	Novel
**73**		AX-94658866	3B	789.75	BLINK	E1,E3, CE	3.11—3.28	0.09	2.71—3.37	NA	PDL	––––––––-	––––––––-	
**74**		AX-95630121*	4A	708.68	FarmCPU	E1, E2, E3, CE	3.83—6.21	0.24	(−2.92)—(−2.37)	2.85—7.04	FLA, PDL	S4A_710817792	710.82	Jamil et al. 2019
					BLINK							AX-109845420	716.97	Hu et al. 2021
**75**		AX-94419770	4A	603.71	FarmCPU	E1, E2, E3, CE	3.44—4.81	0.30	2.19—2.36	NA	––––––––-	––––––––-	––––––––-	Novel
**76**		AX-94934730*	5A	608.62	FarmCPU	E2, E3	5.07—7.11	0.31	(−2.20)—(−2.72)	4.26—9.63	FLA	S5A_625839432; RAC875_c50143_604	625.84; 617.98	Jamil et al. 2019; Gao et al. 2021
					BLINK									
**77**		AX-95126265	6A	581.84	FarmCPU	E1, E2, CE	3.16—5.12	0.50	(−2.11)—(−1.90)	NA	––––––––-	BobWhite c15758_79	595.37	Tian et al. 2023
**78**		AX-95630592*	6B	686.80	FarmCPU	E1, E2, E3, CE	5.25—7.06	0.49	(−3.33)—(−2.52)	4.74—12.09	FLA, PDL	AX-108888670	690.02	Hu et al. 2021
					BLINK									
**79**		AX-94465993	6D	288.56	FarmCPU	E1, E2, E3, CE	3.02—4.66	0.49	8.01—11.25	NA	––––––––-	––––––––-	––––––––-	Novel
					BLINK	E1, E3					––––––––-	––––––––-	––––––––-	
**80**		AX-95118008	6D	434.51	FarmCPU	E2, E3	3.18—5.05	0.49	2.05—2.13	NA	––––––––-	––––––––-	––––––––-	
**81**		AX-94477352	7A	628.94	FarmCPU	E2, E3	3.16—3.26	0.50	13.09—14.59	NA	––––––––-	S7A_618498297	618.50	Jamil et al. 2019
**82**		AX-94441941	7B	116.36	FarmCPU	E2, E3	3.35—3.64	0.50	17.58—20.01	NA	––––––––-	––––––––-	––––––––-	Novel
**83**		AX-95172383	7B	114.47	FarmCPU	E2, E3	3.01—3.81	0.50	(−17.52)—(−14.04)	NA	––––––––-	––––––––-	––––––––-	
**84**		AX-94647729*	7D	71.49	FarmCPU	E1, E2	5.07—7.11	0.50	(−13.17)—(−13.14)	56.67—62.37	––––––––-	––––––––-	––––––––-	
**85**		AX-94878859	7D	17.64	FarmCPU	E1, E2	3.07—3.08	0.47	4.86—5.48	NA	––––––––-	––––––––-	––––––––-	
**86**		AX-95146558	7D	599.14	FarmCPU	E1, E2, CE	3.09—3.72	0.47	4.01—4.43	NA	––––––––-	––––––––-	––––––––-	
**87**		AX-95203773*	7D	7.28	FarmCPU	E1, E2, E3, CE	3.06—5.44	0.17	2.20—2.64	4.02—5.71	––––––––-	RAC875_rep_c112729_702	8.34	Tian et al. 2023
					BLINK	E1, E2, CE					––––––––-			
**88**	**PDL**	AX-94517884	1B	686.14	BLINK	E1, E2, CE	3.02—3.09	0.49	(−7.32)—(−7.02)	NA	FLA	––––––––-	––––––––-	Novel
**89**		AX-94486441*	2A	702.11	FarmCPU	E1, E2, E3, CE	3.45—7.82	0.34	(−1.64)—(- 1.10)	9.18—12.25	FLA	Excalibur_c7282_285	710.10	Sallam et al. 2026
					BLINK	E2, E3, CE	6.82—9.71							
**90**		AX-94658866	3B	789.75	FarmCPU	E1, E2	3.56—3.76	0.09	1.66—1.83	NA	PH	RAC875_c2340_616	796.31	Sallam et al. 2026
**91**		AX-95150153*	3B	667.95	FarmCPU	E2, E3, CE	4.87—6.23	0.44	3.41—3.61	13.03	––––––––-	––––––––-	––––––––-	Novel
**92**		AX-95630121*	4A	708.68	BLINK	E1, E2, E3, CE	3.85—6.07	0.24	(−1.75)—(−1.28)	8.10—9.71	FLA, PH	BS00039641_51	702.07	Sallam et al. 2026
**93**		AX-94432807	5A	48.17	BLINK	E1, E2	3.05—3.18	0.42	1.34—1.42	NA	––––––––-	––––––––-	––––––––-	Novel
**94**		AX-95630592	6B	686.80	FarmCPU	E1, E3, CE	3.17—4.72	0.49	(−7.84)—(−1.10)	NA	FLA, PH	––––––––-	––––––––-	
**95**		AX-94847283	6B	687.73	FarmCPU	E1, E2	4.45—4.86	0.47	(−2.08)—(−1.46)	NA	FLA	––––––––-	––––––––-	
**96**		AX-95207937*	7D	6.13	FarmCPU	E1, E2, E3, CE	3.38—6.22	0.15	1.86—2.03	9.22—13.34	––––––––-	S7D_5101668	5.10	Sallam et al. 2026
**97**	**SL**	AX-94720138*	1A	579.98	FarmCPU	E1, E2, E3, CE	3.22—5.78	0.45	(−0.49)—(−0.36)	6.18—6.29	GPS	BobWhite_c6664_644	574.94	Liu et al. 2018a, b, c, d
					BLINK	E1, E2, E3, CE								
**98**		AX-94800178	1A	579.98	BLINK	E1, E2, E3, CE	3.02—3.41	0.46	(−0.43)—(−0.40)	NA	––––––––-	––––––––-	––––––––-	Novel
**99**		AX-94454534*	1B	523.60	FarmCPU	E2, E3, CE	9.76—11.57	0.17	(−0.88)—(−0.77)	16.92—21.09	GW	AX-95255966	539.37	Hu et al. 2021
					BLINK	E2, E3								
**100**		AX-94858787	3A	131.46	FarmCPU	E1, E2, E3	3.04—3.52	0.06	(−0.64)—(−0.55)	NA	SNS, GPS	––––––––-	––––––––-	Novel
**101**		AX-94534307*	3D	511.30	FarmCPU	E2, E3, CE	4.77—5.28	0.12	(−0.59)—(−0.54)	5.56	GPS	––––––––-	––––––––-	
**102**		AX-95094133*	4B	416.31	FarmCPU	E1, E2, E3, CE	3.35—6.83	0.48	0.99—1.49	21.50—30.73	DTM, SNS, GPS, GW	Tdurum_contig52805_183	404.51	Gao et al. 2021
					BLINK	E1, E2								
**103**		AX-94720837	5B	372.65	BLINK	E1, E2	3.14—3.21	0.48	1.49—1.64	NA	––––––––-	––––––––-	––––––––-	Novel
**104**		AX-94696367	7D	550.85	BLINK	E1, E3, CE	3.09—3.14	0.25	0.37—0.38	NA	SNS, GPS	D_contig37514_120	568.95 Mb	Tian et al. 2023
**105**	**SNS**	AX-94394608*	1B	378.889558	FarmCPU	E1, E3, CE	8.96—14.64	0.16	(−1.13)—(−1.38)	13.12—17.39	GPS	––––––––-	––––––––-	Novel
					BLINK						––––––––-	––––––––-	––––––––-	
**106**		AX-94858787*	3A	131.458684	FarmCPU	E1, E32, E3, CE	4.37—5.46	0.06	(−1.22)—(−0.96)	7.40—7.60	SL, GPS	––––––––-	––––––––-	
**107**		AX-94496249	3A	506.434834	BLINK	E1, E3, CE	3.30—3.52	0.17	0.69—0.71	NA	––––––––-	Kukri_c7218_1145	504.8	Liu et al. 2018a, b, c, d
**108**		AX-94508109	3A	539.683781	BLINK	E1, E2	3.27—3.76	0.24	0.22—0.67	NA	––––––––-	––––––––-	––––––––-	Novel
**109**		AX-94520713	3A	478.256902	BLINK	E1, E3	3.61—3.79	0.25	0.61—0.62	NA	––––––––-	––––––––-	––––––––-	
**110**		AX-94404286	3A	538.639389	BLINK	E1, E2, E3, CE	3.05—3.26	0.25	0.57—0.59	NA	––––––––-	––––––––-	––––––––-	
**111**		AX-94702170	3B	9.690639	FarmCPU	E1, E3, CE	4.06—4.48	0.19	(−0.54)—(−0.51)	NA	YPP	Tdurum_contig28100_112	10.75	Sun et al. 2017
												BobWhite_c15529_288	10.41	Sun et al. 2017
**112**		AX-94714284	3B	507.290841	BLINK	E1, E3, CE	3.11—3.51	0.15	0.73—0.77	NA	––––––––-	––––––––-	––––––––-	Novel
**113**		AX-94387510	3B	536.268014	BLINK	E1, E2, E3, CE	3.17—3.34	0.22	0.59—0.62	NA	––––––––-	––––––––-	––––––––-	
**114**		AX-94599436	3D	412.095215	BLINK	E1,E3	3.54—3.66	0.24	0.59—0.60	NA	––––––––-	AX-110048186	400.50	Xu et al. 2024
**115**		AX-94872610	3D	416.582799	BLINK	E1, E2	3.31—3.70	0.25	0.61—0.64	NA	––––––––-	AX-94648607	419.90	Li et al. 2019a, b
**116**		AX-95094133*	4B	416.306311	FarmCPU	E1,E2, E3, CE	5.44—6.45	0.48	1.76—2.00	28.20—31.63	DTM, SL, GPS, GW	––––––––-	––––––––-	Novel
**117**		AX-94696020	6B	134.808138	FarmCPU	E2, E3, CE	4.12—4.33	0.17	(−0.94)—(−0.93)	NA	––––––––-	––––––––-	––––––––-	
**118**		AX-94413894	7D	550.173694	BLINK	E1, E2, E3, CE	3.54—3.59	0.29	0.59—0.62	NA	EL	––––––––-	––––––––-	
**119**		AX-94653866	7D	559.403179	BLINK	E1, E2, E3, CE	3.28—3.54	0.24	0.57—0.63	NA	––––––––-	––––––––-	––––––––-	
**120**		AX-94696367	7D	550.850424	FarmCPU	E1, E2, E3, CE	3.74—4.93	0.25	0.57—0.65	NA	SL, GPS	––––––––-	––––––––-	
					BLINK	E1, E2, E3						––––––––-	––––––––-	
**121**	**GPS**	AX-94720138	1A	579.98	FarmCPU	E1, E2, E3, CE	3.03—3.86	0.45	(−1.62)—(−1.29)	NA	SL	––––––––-	––––––––-	Novel
**122**		AX-94394608*	1B	378.89	FarmCPU	E1, E2, CE	7.24—9.41	0.16	(−3.24)—(−2.88)	13.11—13.65	GPS	––––––––-	––––––––-	
					BLINK							––––––––-	––––––––-	
**123**		AX-94858787	3A	131.46	FarmCPU	E1,E3	3.08—3.86	0.06	(−2.51)—(−2.39)	NA	SL, SNS	wsnp_Ex_c1763_3333974	134.6	Liu et al. 2018a, b, c, d
**124**		AX-95241788	3D	358.69	FarmCPU	E1	3.16—3.67	0.23	1.42—1.55	NA	––––––––-	––––––––-	––––––––-	Novel
**125**		AX-94534307	3D	511.30	BLINK	E1, E2, CE	3.49—3.44	0.12	(−2.34)—(−2.21)	NA	SL	––––––––-	––––––––-	
**126**		AX-95094133	4B	416.31	FarmCPU	E1, E2, E3, CE	3.21—3.58	0.48	3.64—5.34	NA	DTM, SL, SNS, GW	––––––––-	––––––––-	
					BLINK							––––––––-	––––––––-	
**127**		AX-94749511	6B	707.24	FarmCPU	E1, E2, CE	3.21—3.47	0.47	1.57—1.78	NA	––––––––-	––––––––-	––––––––-	
**128**		AX-94696367	7D	550.85	FarmCPU	E1, E2	3.17—3.92	0.25	1.29—1.89	NA	SL, SNS	––––––––-	––––––––-	
					BLINK							––––––––-	––––––––-	
**129**	**TGW**	AX-94454534	1B	523.60	BLINK	E1, E2	4.05—4.14	0.17	(−1.38)—(−1.34)	NA	SL	wsnp_Ex_c1600_3051075	524.15	Schierenbeck et al. 2021
**130**		AX-94725733*	1B	152.49	FarmCPU	E1, E2, E3, CE	3.56—10.68	0.06	(−5.07)—(−2.57)	25.03—29.29	YPP	––––––––-	––––––––-	Novel
					BLINK	E1, E2, E3						––––––––-	––––––––-	
**131**		AX-94534016	2D	82.11	FarmCPU	E1, E2, CE	3.64—3.73	0.13	(−0.92)—(−0.89)	NA	––––––––-	––––––––-	––––––––-	
**132**		AX-94703749	3A	540.24	FarmCPU	E1, E2, E3, CE	3.27—3.84	0.27	0.74 −1.00	NA	––––––––-	––––––––-	––––––––-	
**133**		AX-94433908	4A	681.36	BLINK	E1, E2	3.00—3.04	0.07	(−1.72)—(−1.69)	NA	––––––––-	S4A_681180933	681.18	Bhatta et al. 2018
**134**		AX-95094133	4B	416.31	FarmCPU	E1, E2	4.92—5.27	0.48	2.97—3.09	NA	DTM, SL, SNS, GPS	S4B_407743714	407.74	Bhatta et al. 2018
**135**		AX-95165912	4B	599.23	BLINK	E1, E2, E3, CE	3.50—4.99	0.07	(−2.36)—(−1.95)	NA	YPP	––––––––-	––––––––-	Novel
**136**		AX-94566370*	5A	20.82	FarmCPU	E1, E2, E3, CE	4.08—5.98	0.25	(−1.17)—(−0.96)	3.10—3.14	––––––––-	––––––––-	––––––––-	
**137**		AX-94384625	5B	122.42	FarmCPU	E1, E2, CE	4.23—4.74	0.09	1.46—1.56	NA	––––––––-	*qTGW5B.2*	114.40–211.80	Pang et al. 2020
**138**		AX-94432536*	5B	545.80	FarmCPU	E1, E2, E3, CE	4.54—8.25	0.12	(−2.12)—(−1.75)	7.46—7.68	YPP	AX-109312888	545.17	Li et al. 2019a, b
**139**		AX-95077961*	5B	458.05	FarmCPU	E1, E2, CE	8.52—9.22	0.08	2.71—2.73	14.41—14.72	YPP	*RAC875_c9150_2945 (IWB61034)*	459.48	Schierenbeck et al. 2021
**140**		AX-95091214	6B	673.76	FarmCPU	E1, E2	3.12—4.54	0.38	1.06—1.23	NA	––––––––-	*Qtgw.ahau-6B.1*	677.70—679.60	Cao et al. 2020
					BLINK	E1, E2					––––––––-			
**141**		AX-95209949	7A	91.32	FarmCPU	E1, E3	4.04—4.97	0.11	(−1.69)—(−1.60)	NA	YPP	wsnp_Ex_c2268_4251636	93.08	Liu et al. 2018a, b, c, d
**142**		AX-94402434	7D	415.39	FarmCPU	E1, E2	4.00—4.21	0.34	(−1.04)—(−1.01)	NA	––––––––-	––––––––-	––––––––-	Novel
**143**		AX-94678664	7D	108.21	FarmCPU	E1, E2, CE	3.76—4.37	0.49	1.17—1.26	NA	––––––––-	––––––––-	––––––––-	
**144**	**YPP**	AX-94725733*	1B	152.49	FarmCPU	E1, E2, CE	3.47—11.62	0.06	(−3.13)—(−1.32)	28.43—33.36	GW	S1B_164107163	164.11	Jamil et al. 2019
					BLINK									
**145**		AX-95679601	1B	201.44	FarmCPU	E1, E2	3.90—4.29	0.48	2.56—2.68	NA	––––––––-	Tdurum_contig6949_697	208.49	Ahmed et al. 2023
**146**		AX-94702170	3B	9.69	FarmCPU	E2, E3	3.24—3.68	0.19	(−0.76)—(−0.60)	NA	SNS	*Yld.cim-3BS.2*	5.60	Juliana et al. 2021
**147**		AX-95165912	4B	599.23	FarmCPU	E1, E2, E3	3.13—4.97	0.07	(−1.61)—(−1.24)	NA	GW	4B_608802048_608802085	608.88	Chidzanga et al. 2022
					BLINK	E1, E2, E3, CE								
**148**		AX-94432536*	5B	545.80	FarmCPU	E1, E2, CE	4.04—5.61	0.12	(−1.14)—(−1.04)	4.47	GW	*Yld.cim-5BL.2*	550.91	Juliana et al. 2021
**149**		AX-95077961*	5B	458.05	FarmCPU	E1, E2, CE	8.05—8.93	0.08	1.70—1.91	8.90—14.21	GW	––––––––-	––––––––-	Novel
**150**		AX-94659552*	5D	233.41	FarmCPU	E1, E2, CE	4.80—6.26	0.09	1.06—1.33	7.18	––––––––-	––––––––-	––––––––-	
**151**		AX-95217910	6D	450.21	FarmCPU	E1, E3, CE	3.18—3.79	0.48	0.62—0.73	NA	PDL	S6D_471249189	471.25	Jamil et al. 2019
**152**		AX-95209949*	7A	91.32	FarmCPU	E1, E2, CE	3.25—7.35	0.11	(−1.51)—(−0.94)	5.73—6.71	GW	––––––––-	––––––––-	Novel

CS: Cold stress tolerance; EL: Electrolyte leakage; DTF: Days to flowering; DTM: Days to maturity; FLA: Flag leaf area; PH: Plant height; PDL: Peduncle length; SL: Spike length; SNS: Number of spikelets per spikes; TGW: Thousand grain weight; GPS: Grain per spikes; YPP: Yield per plant; Chr: Chromosome; Pos: Position; Env: Environment; MAF: Marker allele frequency; PVE: Phenotypic variance; * represents high-confidence MTAs

From these stable SNPs, four major LD blocks were identified on chromosomes 1B, 1D, and 5A. The LD block on chromosome 1B (69 kb; 69,605,547–69,684,979 bp) comprised two haplotypes: HAP-1 (286 genotypes; EMM 2.67) and HAP-2 (26 genotypes; 1.48). On chromosome 1D, two LD blocks were detected at 0 kb (414,508,531–414,508,759 bp) and 38 kb (408,207,062–408,245,547 bp), forming haplotypes HAP-3 (53 genotypes; 2.99) and HAP-4 (224 genotypes; 2.38), and HAP-5 (72 genotypes; 2.91) and HAP-6 (239 genotypes; 2.45), respectively. On chromosome 5 A, fourth LD block were identified at 0 kb (399,369,677–399,369,678 bp) with two haplotypes: HAP-7 (207 genotypes; 2.40) and HAP-8 (84 genotypes; 2.60).

**Fig. 5:**
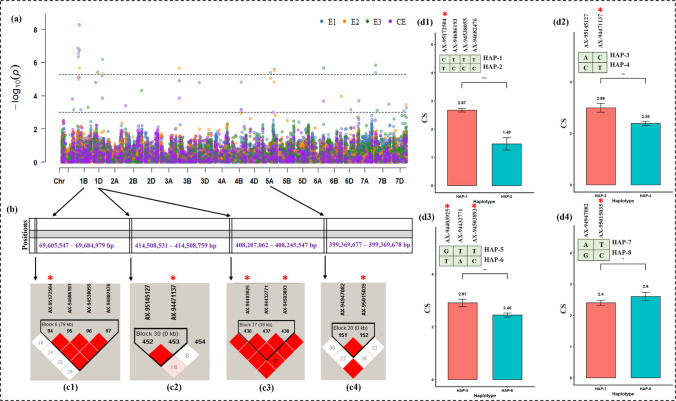
Significant haplotypes associated with cold stress tolerance (CS) on wheat chromosomes 1B, 1D and 5A. (**a**) Manhattan plot highlighting SNP associations for CS across the 21 wheat chromosomes. (**b**) Range of physical positions of SNPs within each identified Linkage disequilibrium (LD) blocks. (c1-c4) Show LD heatmaps among SNPs within haplotype blocks on chromosomes 1B, 1D and 5A. (d1-d4) Illustrate the phenotypic variation in CS among different haplotypes within each LD block

FDR-adjusted comparisons indicated that HAP-2 (first LD block), HAP-4 (second LD block), HAP-6 (third LD block), and HAP-7 (fourth LD block) exhibited significantly lower CS scores than their respective alternative haplotypes (Fig. 5).

#### Electrolyte leakage index (EL)

GWAS using FarmCPU and BLINK identified 11 and 12 MTAs in E1, 13 and 12 in E2, 13 and 9 in E3, and 10 and 28 in the CE dataset, respectively (Table S7). Among these, 12 stable MTAs were distributed across chromosomes 1B (2), 1D (3), 4B (1), 5 A (2), 6 A (1), 6D (1), 7B (1) and 7D (1), with − log₁₀(P) values ranging from 3.12 to 7.72 (Table 4). Seven MTAs passed the Bonferroni threshold.

Two major LD blocks were detected on chromosomes 1D (414,508,531–414,508,759 bp) and 5 A (399,369,677–399,369,678 bp), corresponding to the same regions identified for CS.

#### Days to flowering (DTF)

GWAS using FarmCPU and BLINK identified 11 and 10 MTAs in E1, 12 and 13 in E2, 14 and 3 in E3, and 12 and 15 in the CE dataset, respectively (Table S8). Among these, 18 stable MTAs were identified and mapped across seven chromosomes: 1 A (3), 1B (1), 1D (9), 3 A (1), 5 A (1), 6 A (2), and 7 A (1). Their − log₁₀(P) values ranged from 3.00 to 13.69 (Table 4), of which four were Bonferroni-corrected MTAs.

Five major LD blocks were identified on chromosomes 1B, 1D, and 6A. The first LD block on chromosome 1B spanned 83 kb (681,196,663–681,280,170 bp) and comprised four haplotypes: HAP-1 (81 genotypes; EMM 179.30), HAP-2 (115 genotypes; 175.40), HAP-3 (86 genotypes; 173.60), and HAP-4 (13 genotypes; 178.10). On chromosome 1D, two LD blocks were detected at 44 kb (408,200,786–408,245,547 bp) and 0 kb (414,508,531–414,508,759 bp). The second LD block comprised two haplotypes: HAP-5 (72 genotypes; 177.90) and HAP-6 (237 genotypes; 175.10), whereas the third LD block comprised HAP-7 (53 genotypes; 178.40) and HAP-8 (224 genotypes; 174.60).

On chromosome 6 A, two LD blocks were detected at 19 kb (616,909–636,836 bp) and 138 kb (581,839,269–581,977,577 bp). The fourth LD block comprised three haplotypes: HAP-9 (162 genotypes; 174.90), HAP-10 (92 genotypes; 177.50), and HAP-11 (13 genotypes; 175.40). The fifth LD block comprised four haplotypes: HAP-12 (118 genotypes; 175.70), HAP-13 (110 genotypes; 176.90), HAP-14 (34 genotypes; 172.40), and HAP-15 (32 genotypes; 176.10).

**Fig. 6:**
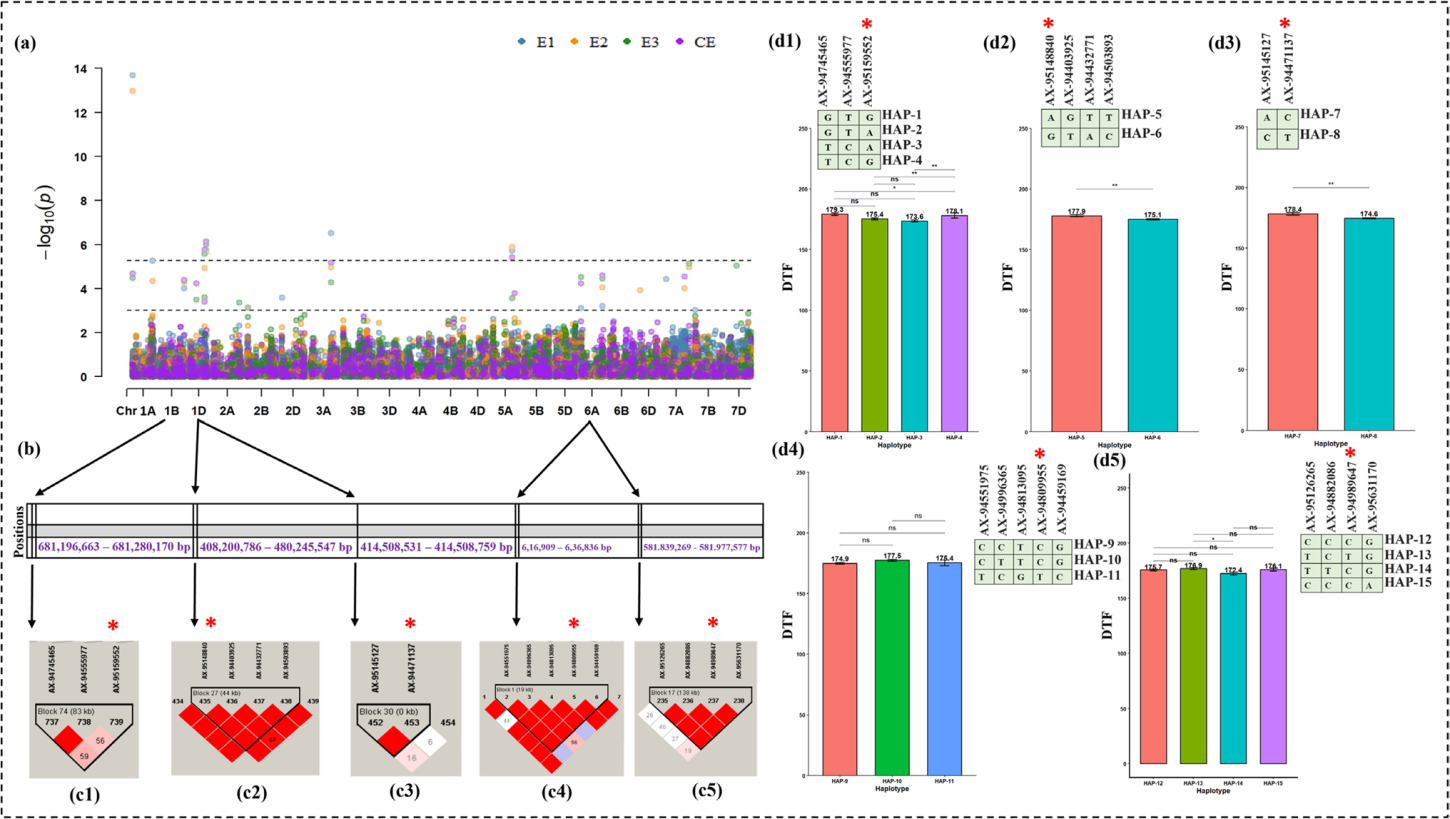
Significant haplotypes associated with Days to flowering (CS) on wheat chromosomes 1B, 1D and 6A. (**a**) Manhattan plot highlighting SNP associations for DTF across the 21 wheat chromosomes. (**b**) Range of physical positions of SNPs within each identified Linkage disequilibrium (LD) block. (c1-c4) Show LD heatmaps among SNPs within haplotype blocks on chromosomes 1B, 1D and 6A. (d1-d4) Illustrate the phenotypic variation in DTF among different haplotypes within each LD block

Comparisons based on FDR-adjusted p-values revealed that HAP-3 (first LD block), HAP-6 (second LD block), HAP-8 (third LD block), and HAP-14 (fifth LD block) exhibited significantly lower DTF than their respective alternative haplotypes, whereas no significant differences were observed among haplotypes within the fourth LD block (Fig. 6).

#### Days to maturity (DTM)

GWAS using FarmCPU and BLINK identified 11 and 3 MTAs in E1, 12 and 2 MTAs in E2, 9 and 5 MTAs in E3, and 12 and 4 MTAs in the CE dataset, respectively (Table S9). Among these, 10 stable MTAs were identified across nine chromosomes: 1 A (1), 1B (2), 1D (1), 3 A (1), 4B (1), 5 A (1), 6 A (1), 6D (1) and 7B (1). Their − log₁₀(P) values ranged from 3.01 to 11.50 (Table 4), including three Bonferroni-corrected MTAs.

Two major LD blocks were identified on chromosomes 1D and 6A. The first LD block on chromosome 1D at 0 kb (414,508,531–414,508,759 bp) comprised two haplotypes: HAP-1 (53 genotypes; EMM 237.50) and HAP-2 (224 genotypes; 235.00). The second LD block on chromosome 6 A at 138 kb (581,839,269–581,977,577 bp) comprised four haplotypes: HAP-3 (118 genotypes; 235.50), HAP-4 (101 genotypes; 236.50), HAP-5 (34 genotypes; 233.60), and HAP-6 (32 genotypes; 236.10).

Comparisons based on FDR-adjusted p-values indicated that HAP-2 (first LD block) exhibited significantly lower DTM than the alternative haplotype, whereas no significant differences were observed among haplotypes within the second LD block (Fig. S3).

#### Flag leaf area (FLA)

GWAS using FarmCPU and BLINK identified 24 and 23 MTAs in E1, 27 and 13 MTAs in E2, 8 and 7 MTAs in E3, and 32 and 4 MTAs in the CE dataset, respectively (Table S10). Among these, 17 stable MTAs were detected across nine chromosomes: 1B (1), 2 A (1), 4 A (1), 4B (1), 5 A (6), 5D (1), 6 A (2), 6B (3), and 6D (1). Their − log₁₀(P) values ranged from 3.01 to 7.07 (Table 4), including three Bonferroni-corrected MTAs.

Two major LD blocks were identified on chromosomes 1B and 5A. The first LD block on chromosome 1B at 71 kb (686,067,963–686,138,982 bp) comprised four haplotypes: HAP-1 (103 genotypes; EMM 44.21), HAP-2 (104 genotypes; 27.21), HAP-3 (57 genotypes; 44.93), and HAP-4 (53 genotypes; 26.94). The second LD block on chromosome 5 A at 64 kb (609,210,472–609,274,480 bp) comprised two haplotypes: HAP-5 (222 genotypes; 34.63) and HAP-6 (85 genotypes; 37.69).

Comparisons based on FDR-adjusted p-values showed that HAP-3 (first LD block) and HAP-6 (second LD block) exhibited significantly higher FLA than their respective alternative haplotypes (Fig. S4).

#### Plant height (PH)

GWAS using FarmCPU and BLINK identified 12 and 8 MTAs in E1, 28 and 4 MTAs in E2, 15 and 15 MTAs in E3, and 12 and 11 MTAs in CE dataset, respectively (Table S11). Among these, 16 stable MTAs were identified across ten chromosomes: 2D (1), 3B (1), 4 A (2), 5 A (1), 6 A (1), 6B (1), 6D (2), 7 A (1), 7B (2) and 7D (4), with − log₁₀(P) values ranged from 3.01 to 7.11 (Table 4). Of these, five MTAs exceeded the Bonferroni-corrected threshold.

Three major LD blocks were identified on chromosomes 3B and 7D. The first LD block on chromosome 3B at 7 kb (789,751,374–789,758,886 bp) comprised two haplotypes: HAP-1 (301 genotypes; EMM 111.00) and HAP-2 (27 genotypes; 106.00). The second LD block on chromosome 7D at 1 kb (71,491,075–71,492,211 bp) comprised HAP-3 (170 genotypes; 102.00) and HAP-4 (167 genotypes; 119.40). The third LD block on chromosome 7D at 10 kb (17,627,873–17,638,792 bp) comprised HAP-5 (146 genotypes; 122.30) and HAP-6 (167 genotypes; 100.00).

Comparisons based on FDR-adjusted p-values indicated that HAP-2 (first LD block) and HAP-3 (second LD block) exhibited significantly lower PH than their respective alternative haplotypes, whereas no significant differences were observed among haplotypes within the third LD block (Fig. S5).

#### Peduncle length (PDL)

GWAS using FarmCPU and BLINK identified 43 and 8 MTAs in E1, 12 and 14 MTAs in E2, 11 and 9 MTAs in E3, and 13 and 10 MTAs in the CE dataset, respectively (Table S12). Among these, nine stable MTAs were detected across seven chromosomes: 1B (1), 2 A (1), 3B (2), 4 A (1), 5 A (1), 6B (2), and 7D (1). Their − log₁₀(P) values ranged from 3.02 to 9.71 (Table 4), including four Bonferroni-corrected MTAs.

Three major LD blocks were identified on chromosomes 1B, 3B, and 5A. The first LD block on chromosome 1B at 71 kb (686,067,963–686,138,982 bp) comprised four haplotypes: HAP-1 (103 genotypes; EMM 35.50), HAP-2 (104 genotypes; 26.58), HAP-3 (57 genotypes; 36.24), and HAP-4 (53 genotypes; 25.93). The second LD block on chromosome 3B at 7 kb (789,751,374–789,758,886 bp) comprised two haplotypes: HAP-5 (301 genotypes; 31.44) and HAP-6 (26 genotypes; 26.20). The third LD block on chromosome 5 A at 4 kb (48,170,260–48,174,477 bp) comprised two haplotypes: HAP-7 (132 genotypes; 32.08) and HAP-8 (85 genotypes; 30.29).

Comparisons based on FDR-adjusted p-values showed that HAP-3 (first LD block) and HAP-5 (second LD block) exhibited significantly higher PDL than their respective alternative haplotypes, whereas no significant differences were detected among haplotypes within the third LD block (Fig. S6).

#### Spike length (SL)

GWAS using FarmCPU and BLINK identified 9 and 11 MTAs in E1, 11 and 10 MTAs in E2, 11 and 7 MTAs in E3, and 9 and 8 MTAs in the CE dataset, respectively (Table S13). Among these, eight stable MTAs were detected across seven chromosomes: 1 A (2), 1B (1), 3 A (1), 3D (1), 4B (1), 5B (1), and 7D (1). Their − log₁₀(P) values ranged from 3.02 to 11.57 (Table 4), including four Bonferroni-corrected MTAs.

Three major LD blocks were identified on chromosomes 1 A, 1B, and 3D. The first LD block on chromosome 1 A at 34 kb (579,980,075–580,014,319 bp) comprised five haplotypes: HAP-1 (69 genotypes; EMM 10.12), HAP-2 (63 genotypes; 10.21), HAP-3 (68 genotypes; 11.25), HAP-4 (28 genotypes; 9.29), and HAP-5 (54 genotypes; 9.85).The second LD block on chromosome 1B at 7 kb (523,591,335–523,598,614 bp) comprised two haplotypes: HAP-6 (263 genotypes; 9.91) and HAP-7 (41 genotypes; 11.59).The third LD block on chromosome 3D at 25 kb (511,302,538–511,327,673 bp) comprised three haplotypes: HAP-8 (162 genotypes; 10.60), HAP-9 (128 genotypes; 9.44), and HAP-10 (29 genotypes; 10.73).

Comparisons based on FDR-adjusted p-values indicated that HAP-3 (first LD block), HAP-7 (second LD block), and HAP-10 (third LD block) exhibited significantly higher SL than their respective alternative haplotypes (Fig. S7).

#### Spikelet number per spike (SNS)

GWAS using FarmCPU and BLINK identified 21 and 27 MTAs in E1, 9 and 13 MTAs in E2, 10 and 7 MTAs in E3, and 7 and 21 MTAs in CE dataset, respectively (Table S14). Among these, 16 stable MTAs were identified and mapped across seven chromosomes: 1B (1), 3 A (5), 3B (3), 3D (2), 4B (1), 6B (1), and 7D (3). Their − log₁₀(P) values ranged from 3.05 to 14.64 (Table 4), including three Bonferroni-corrected MTAs.

Two major LD blocks were identified on chromosomes 1B and 6B. The first LD block on chromosome 1B at 0 kb (378,888,978–378,889,558 bp) comprised two haplotypes: HAP-1 (266 genotypes; EMM 18.34) and HAP-2 (37 genotypes; 20.60). The second LD block on chromosome 6B at 11 kb (489,796,020–491,051,329 bp) comprised three haplotypes: HAP-3 (169 genotypes; 16.88), HAP-4 (81 genotypes; 21.21), and HAP-5 (26 genotypes; 22.84).

Comparisons based on FDR-adjusted p-values revealed that HAP-2 (first LD block) exhibited significantly higher SNS than the alternative haplotype, whereas no significant differences were detected among haplotypes within the second LD block (Fig. S8).

#### Grain per spike (GPS)

GWAS using FarmCPU and BLINK identified 8 and 5 MTAs in E1, 9 and 5 MTAs in E2, 5 and 4 MTAs in E3, and 9 and 7 MTAs in CE dataset, respectively (Table S15). Among these, eight stable MTAs were detected across seven chromosomes: 1 A (1), 1B (1), 3 A (1), 3D (2), 4B (1), 6B (1) and 7D (1). Their − log₁₀(P) values ranged from 3.03 to 9.41 (Table 4), including one Bonferroni-corrected MTAs.

Three major LD blocks were identified on chromosomes 1 A, 1B, and 3D. The first LD block on chromosome 1 A at 34 kb (579,980,075–580,014,319 bp) comprised five haplotypes: HAP-1 (69 genotypes; EMM 41.81), HAP-2 (63 genotypes; 41.61), HAP-3 (68 genotypes; 45.71), HAP-4 (28 genotypes; 40.02), and HAP-5 (54 genotypes; 39.07). The second LD block on chromosome 1B at 0 kb (378,888,978–378,889,558 bp) comprised two haplotypes: HAP-6 (266 genotypes; 40.94) and HAP-7 (37 genotypes; 46.97). The third LD block on chromosome 3D at 25 kb (511,302,538–511,327,673 bp) comprised three haplotypes: HAP-8 (162 genotypes; 41.95), HAP-9 (128 genotypes; 40.40), and HAP-10 (29 genotypes; 45.88).

Comparisons based on FDR-adjusted p-values indicated that HAP-7 (second LD block) and HAP-10 (third LD block) exhibited significantly higher GPS than their respective alternative haplotypes, whereas no significant differences were detected among haplotypes within the first LD block (Fig. S9).

#### Thousand grain weight (TGW)

GWAS using FarmCPU and BLINK identified 13 and 5 MTAs in E1, 11 and 5 MTAs in E2, 10 and 12 MTAs in E3, and 12 and 20 MTAs in CE dataset, respectively (Table S16). Among these, 15 stable MTAs were detected across ten chromosomes: 1B (2), 2D (1), 3 A (1), 4 A (1), 4B (2), 5 A (1), 5B (3), 6B (1), 7 A (1) and 7D (2). Their − log₁₀(P) values ranged from 3.00 to 10.68 (Table 4), including four Bonferroni-corrected MTAs.

Three major LD blocks were identified on chromosomes 1B and 5B. The first LD block on chromosome 1B at 7 kb (523,591,335–523,598,614 bp) comprised two haplotypes: HAP-1 (263 genotypes; EMM 45.15) and HAP-2 (41 genotypes; 47.27). The second LD block on chromosome 5B at 3 kb (545,791,493–545,795,212 bp) comprised four haplotypes: HAP-3 (178 genotypes; 44.71), HAP-4 (98 genotypes; 46.30), HAP-5 (19 genotypes; 48.64), and HAP-6 (14 genotypes; 45.10). The third LD block on chromosome 5B at 0 kb (458,052,808–458,053,019 bp) comprised two haplotypes: HAP-7 (307 genotypes; 45.78) and HAP-8 (24 genotypes; 42.30).

Comparisons based on FDR-adjusted p-values indicated that HAP-2 (first LD block), HAP-5 (second LD block), and HAP-7 (third LD block) exhibited significantly higher TGW than their respective alternative haplotypes (Fig. S10).

#### Yield per plant (YPP)

GWAS using FarmCPU and BLINK identified 13 and 7 MTAs in E1, 14 and 2 MTAs in E2, 9 and 4 MTAs in E3, and 13 and 4 MTAs in CE dataset, respectively (Table S17). Among these, nine stable MTAs were identified and mapped across seven chromosomes: 1B (2), 3B (1), 4B (1), 5B (2), 5D (1), 6D (1) and 7 A (1). Their − log₁₀(P) values ranged from 3.13 to 11.62 (Table 4). Of these, five MTAs exceeded the Bonferroni-corrected threshold.

Two major LD blocks were identified on chromosome 5B at 3 kb (545,791,493–545,795,212 bp) and 0 kb (458,052,808–458,053,019 bp). The first LD block comprised four haplotypes: HAP-1 (178 genotypes; EMM 13.53), HAP-2 (98 genotypes; 14.82), HAP-3 (19 genotypes; 16.19), and HAP-4 (14 genotypes; 13.50). The second LD block comprised two haplotypes: HAP-5 (307 genotypes; 14.41) and HAP-6 (24 genotypes; 10.20). Comparisons based on FDR-adjusted p-values revealed that HAP-3 (first LD block) and HAP-5 (second LD block) exhibited significantly higher YPP than their respective alternative haplotypes (Fig. S11).

Several MTAs showed pleiotropic effects, being associated with more than one trait. Details of such loci are summarized in Table 4.

### Linkage disequilibrium (LD) and candidate gene (CGs) analyses

**Fig. 7:**
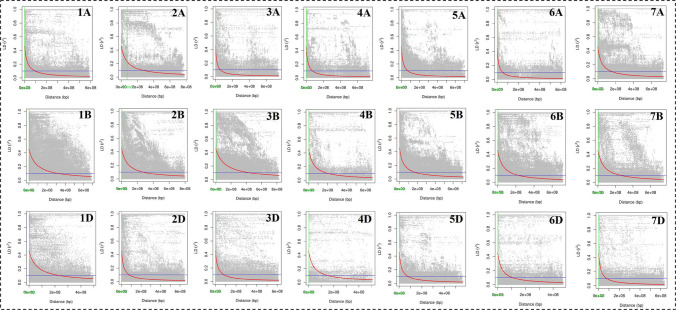
Analysis of linkage disequilibrium (LD) at the chromosome levels. The LD-decay block size (in bp) is depicted in green color

LD analysis at the chromosome level revealed substantial variation in LD block sizes across the genome (Table S18). LD blocks ranged from 0.72 Mb on chromosome 6 A to 7.42 Mb on chromosome 2 A, with an average size of 2.50 Mb per chromosome (Fig. 7). Sub-genome and whole-genome LD patterns for the same panel were reported previously by Kaur et al. (2025), with mean LD decay distances of 5.2 Mb, 5.7 Mb, and 6.9 Mb for the A, B, and D sub-genomes, respectively, while at the whole-genome level, LD decreased gradually with increasing pairwise marker distance.

Using chromosome-specific half-LD decay intervals for each high-confidence MTA, a total of 362 and 1667 putative CGs were identified for CS and yield-related traits, respectively. (Table S19).

Literature mining of putative CGs associated with yield and related traits identified 102 potential genes (Table S21). Subsequent prioritization of the remaining genes using the KnetMiner database revealed an additional 21 genes potentially linked to yield and related traits (Table S20; Fig. S12 – S14). In total, the combined approaches identified 123 high-confidence CGs underlying yield and yield-related traits. Important CGs for DTF and DTM were Glycosyltransferase 2-like, MADS-box TFs, Protein LITTLE ZIPPER, Reticulon, SANT/Myb domain, COBRA etc. Key CGs having potential role in FLA were BTB/POZ domain, Protein LITTLE ZIPPER, Pentatricopeptide repeat etc. Major CGs having potential role in PH and PDL were Protein PELPK1/2, SANT/Myb domain, NAC domain, Cytochrome P450 etc. Potential CGs for SL were Protein kinase domain, Transcription elongation factor 1etc. Key CGs for YPP included Zinc finger C2H2-type, PPC domain, Sulfiredoxin etc. Important CGs for TGW in the vicinity of MTAs included cytochrome P450s, F-box domains, WD40 etc.

**Fig. 8:**
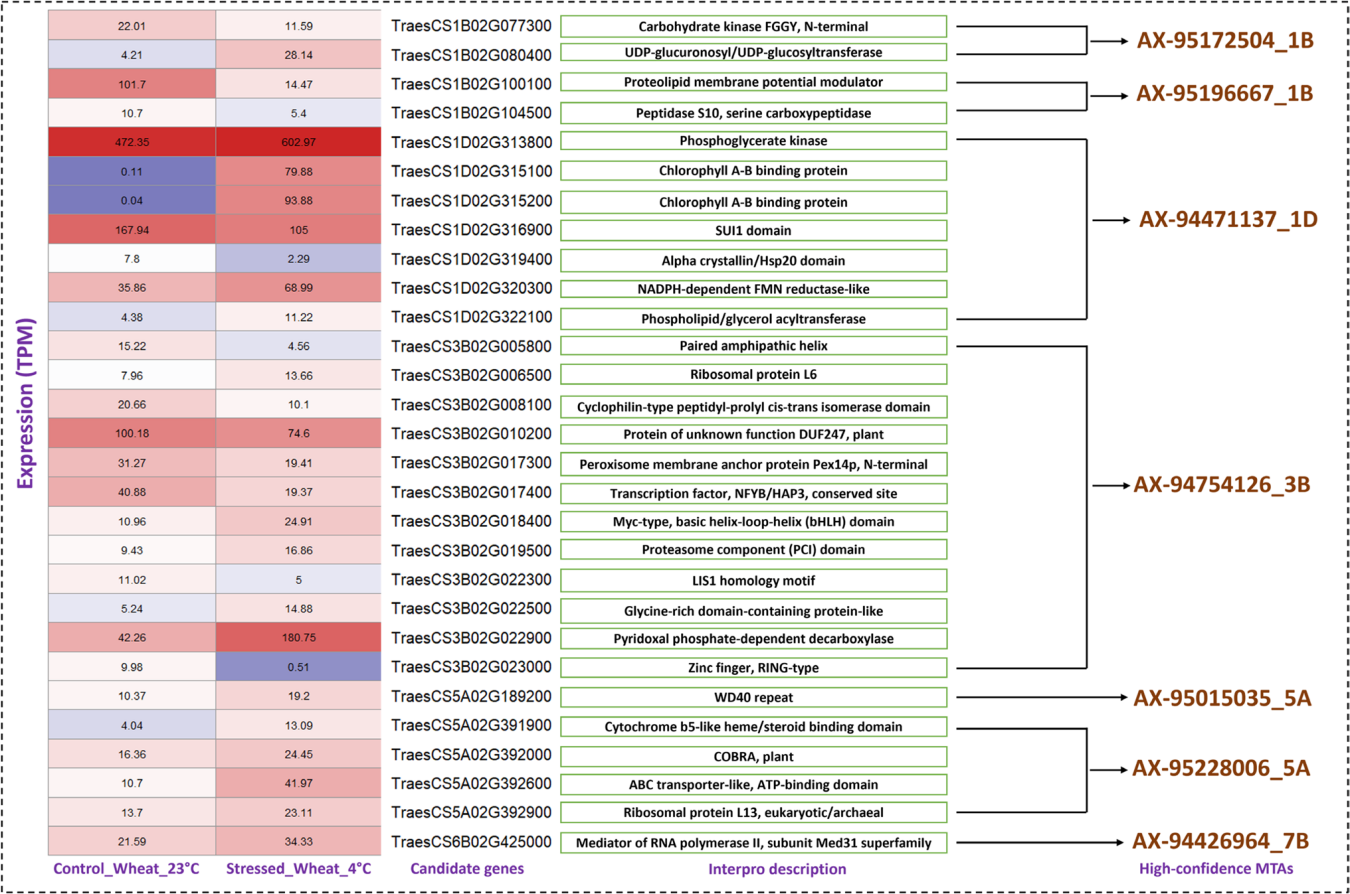
*In sillico* expression profiling and functional annotation of candidate genes associated with high confident MTAs for cold stress tolerance (CS) in wheat. The heatmap shows transcript abundance (TPM) of candidate genes under control (23 °C) and cold stress (4 °C) conditions

For CS, prioritization was done using two complementary approaches. In silico expression analysis identified 110 potential genes, of which 56 CGs exhibited expression levels > 2 TPM (Table S22). From these, a subset of 29 genes showing expression levels ≥ 5 TPM under cold stress conditions were retained as high-confidence candidates (Fig. 8). In parallel, literature mining of the putative CGs identified 16 additional CGs associated with cold stress responses (Table S21). These prioritized CGs are involved in cold stress signalling pathways and encode proteins such as GRAS transcription factors, Universal stress protein A family, NADPH-dependent FMN reductase-like domain, ABC transporters, ATP-binding domain etc.

## Discussion

Wheat, a staple crop cultivated globally in temperate and subtropical regions, serves as a primary source of dietary protein and calories in human diets (De Sousa et al. 2021). Despite global warming, extreme and abnormal cold events still occur frequently in many wheat-growing regions, sharply increasing the risk of cold-induced yield losses (Zhang et al. 2023a). The ideal temperature range for wheat growth is around 22/14℃ (day/night) (Pradhan et al. 2015). In the Kashmir Valley, wheat productivity remains low despite favourable agronomic conditions, largely due to recurrent exposure to sub-zero temperatures during early growth stages. These low-temperature events impair plant development, reduce yield, and significantly prolong the crop cycle, resulting in delays of nearly two months in flowering and maturity. Such delays severely constrain the timely rice–wheat cropping sequence compared with other regions of India (Mir et al. 2025). According to the several previous reports, winter freezing stress can injure seedlings, impair growth and development, damage flag leaves and spikes, reduce grain number per spike, slow grain filling, and ultimately cause partial to complete yield losses (González et al. 2005; Zheng et al. 2018; Su et al. 2022a, b; Pan et al. 2024; Thakur et al. 2010; Hassan et al. 2021). Biologically, these losses are associated with reduced productive tillers, shorter stems, lower leaf area, and diminished photosynthetic capacity (Valluru et al. 2012; Li et al. 2015). Given the magnitude of these effects, elucidating the genetic control of cold stress tolerance and related agronomic traits is critical for expanding wheat cultivation in the Kashmir Valley.

Several efforts have been made to understand the genetic basis of cold stress tolerance and to develop effective strategies for screening and selecting cold-tolerant genotypes (Zhao et al. 2020a; Su et al. 2022a, b; Rojas‐Gutierrez et al. 2022; El Baouchi et al. 2023; Pan et al. 2024; Liu et al. 2025a, b, c). Still, no promising sources have been identified, as the cultivated wheat gene pool lacks sufficient diversity for cold tolerance, highlighting the importance of introducing novel alleles from wild relatives. Aegilops species and durum wheat, progenitors of hexaploid wheat, harbor multiple genes conferring low-temperature resistance (Barashkova 1980; Jia et al. 2013; Limin and Fowler 1985; Masoomi-Aladizgeh et al. 2015; Jin et al. 2018; Leonardis et al. 2007; Bhardwaj & Chaudhry 2025) and have been successfully used to enhance yield under abiotic stress conditions (Kaur et al. 2018; Ma et al. 2023; Taranto et al. 2023; Mokhtari et al. 2024).

To harness this diversity, we utilized a panel of 340 BC₁F₆ introgression lines (S-NILibs) derived from two synthetic hexaploid wheats—syn14170 (PBW114/*Ae. tauschii* acc. pau 14,170) and syn14135 (PDW233/*Ae. tauschii* acc. pau 14,135)—crossed and backcrossed with two advanced breeding lines. This panel has previously been evaluated for drought tolerance (Sharma et al. 2022), yield potential under heat stress (Kaur et al. 2025), and nitrogen use efficiency (Sandhu et al. 2021, 2023; Kumar et al. 2022) and has shown promising results.

Here, field screening under the challenging climatic conditions of Wadura, Kashmir, assessed cold stress traits, including cold stress score (CS) and electrolyte leakage index (EL), as well as ten key yield-contributing traits, DTF, DTM, FLA, PH, PDL, SL, SNS, GPS, YPP, and TGW -all of which influence final yield (Niu et al. 2021; Sattar et al. 2023; Miao et al. 2024; Vicentin et al. 2024). EL, a widely used measure in plant physiology, serves as an indicator of membrane stability under abiotic stresses, including cold stress (Bičárová et al. 2023). All local cultivars exhibited severe injury under the imposed freezing temperatures, confirming the suitability of the experimental conditions to determine the true performance of each genotype, that is, to differentiating tolerant and susceptible genotypes.

ANOVA revealed significant phenotypic variation for all traits, with transgressive segregation and both positive and negative deviations from the recurrent parents, reflecting substantial allelic diversity contributed by parental lines. This highlights the potential of the panel to improve cold tolerance and yield under stress. Transgressive segregation is commonly exploited in crop breeding to enhance yield and stress resilience (Gu et al. 2023). The box-plot distribution observed for all traits across environments indicate quantitative inheritance, likely governed by additive effects of multiple QTLs and genes (Kruse et al. 2017; Jan et al. 2023a ; Bolouri et al. 2023; Zhuang et al. 2025). Several lines displayed both exceptional cold resistance (lower EL and CS) and high yield with early maturity, outperforming their parents. These lines represent promising candidates and can be given due consideration in breeding programs.

Strong positive phenotypic correlations were observed among all yield-related traits under cold stress, consistent with previous reports under several abiotic stresses (Gupta et al. 2007; Rojas‐Gutierrez et al. 2022; Kaur et al. 2025; Saini et al. 2022). In crop improvement efforts, it is beneficial to observe strong positive correlations between yield and related traits, as this allows for the simultaneous enhancement of interconnected traits. Pearson correlation analysis revealed that higher cold tolerance (lower CS and EL) was associated with higher yield and earlier maturity, consistent with prior studies (Rojas‐Gutierrez et al. 2022; Ali et al. 2023; Wang et al. 2024). A strong correlation between CS and EL was also evident, reflecting shared molecular and physiological mechanisms underlying the cold stress response (Masoomi-Aladizgeh et al. 2015; Mir et al. 2019; Jan et al. 2023a).

Due to the complexity of cold tolerance and agronomic traits, it is crucial to identify more influential MTAs. Therefore, GWAS was performed exploiting two multi-locus models (Farm CPU and BLINK). ML-GWAS models are superior to single-locus models for complex traits, as they simultaneously assess all marker effects, reducing false positives and enhancing detection power (Vikas et al. 2022; Mir et al. 2023). Environmentally stable markers (E ≥ 2 and one or both models] ensure consistent trait expression and performance across diverse conditions, represent robust candidates for further validation and potential application in marker-assisted selection (MAS).

As a result, GWAS identified a total of 152 stable MTAs (− log₁₀(P) value range: 3.00–14.64) associated with cold tolerance and yield-related traits, highlighting the significant genetic variation underlying these parameters. To further refine the associations, a second, more stringent significance threshold based on Bonferroni correction for multiple testing was applied. This approach resulted in the identification of 50 high-confidence MTAs (− log₁₀(P) ≥ 5.27) for CS (7), EL (7), DTF (4), DTM (3), FLA (3), PH (5), PDL (4), SL (4), SNS (3), GPS (1), YPP (5), and TGW (4), with R^2^ values ranging from 2.56 to 62.37%, indicating a wide range of explained phenotypic variation (Table 4). However, MTAs that did not surpass the Bonferroni threshold should not be entirely ignored, as several stable MTAs identified before and after correction co-localized with previously reported MTAs/QTLs. Such concordance with earlier studies increases the confidence and robustness of these associations and justifies their inclusion in further discussion.

For CS, 14 stable MTAs were identified. Among these, AX-94670671 and AX-95246613 on chromosome 1 A at 506.68 Mb and 512.25 Mb were located in close proximity to marker wsnp_Ku_c23012_32893918 at 516.37 Mb (Zhang et al. 2023b). On chromosome 1B, AX-95196667 (117.85 Mb) and AX-94708814 (56.17 Mb) were situated near previously reported markers qCT1B.2 (102.1–140.9 Mb) and S1B_56585121 (56.59 Mb), respectively (Pang et al. 2021; Rojas-Gutierrez et al. 2022). Chromosome 1D emerged as a hotspot for cold tolerance in the present study, with four stable MTAs detected between 407.92 and 414.51 Mb and lying-in close proximity to reported markers Kukri_c18413_496 (407.87 Mb) and wsnp_Ex_rep_c66423_64641115 (415.42 Mb) (Zhang et al. 2023b). On chromosome 5 A, AX-95015035 (399.36 Mb) was located near AX-111772046 at 398.04 Mb (Zhang et al. 2023b), while AX-95228006 (590.69 Mb) was close to markers wsnp_Ex_c17763_26513562 at 591.32 Mb (Zhang et al. 2023b), BS00031117_51 at 588.38 Mb (Zhang et al. 2023b) and BS00075959_51 at 588.74 Mb (Zhao et al. 2020a). Previous studies have consistently highlighted the importance of chromosome 5 A in frost tolerance (Tóth et al. 2003; Sofalian et al. 2009; Kruse et al. 2017; Soleimani et al. 2022; Wu et al. 2024). The remaining four MTAs identified in this study appear to be potentially novel, as these genomic regions have not been previously associated with cold or frost stress.

For EL, 12 stable MTAs were identified, of which seven were pleiotropic with CS. The co-localization of these markers validates their strong positive correlation. As suggested by Wang et al. (2012a, b), the observed co-localization may be attributed to pleiotropic effects of individual loci or tight linkage between loci within the same genomic region, which could account for the significant correlations between traits.

For DTF, 18 stable MTAs were detected. AX-95205762-1A at 552.43 Mb was located near markers qHD1A.1 and AX-95113161 at 543.25 Mb and 560.46 Mb, respectively (Pang et al. 2020; Nawade et al. 2025). Chromosome 1D appeared as a hotspot for DTF, with nine stable MTAs identified. Among these, eight MTAs between 408.20 and 414.51 Mb were located near reported markers S1D_416730006 (416.73 Mb) and S1D_417084381 (417.08 Mb) (Jamil et al. 2019). AX-94466657 (564.34 Mb) on chromosome 3 A was located near AX-111630734 at 558.01 Mb (Li et al. 2019a, b), while AX-95251140 (535.73 Mb) on 5 A was close to AX-94935237 at 540.29 Mb (Nawade et al. 2025). On chromosome 6 A, AX-94989647 (581.98 Mb) was located near qHD6A.2 at 572.02 Mb, and AX-94554795 (717.75 Mb) on chromosome 7 A was located near qHD7A.8 at 717.18 Mb (Pang et al. 2020). The remaining five MTAs appear to represent potentially novel loci.

For DTM, ten stable MTAs were identified, of which six were pleiotropic with DTF, confirming their strong positive correlation. Similar co-localization of MTAs for DTF and DTM has also been reported previously in several studies (Yang et al. 2021a, b; Henkrar et al. 2025). AX-94911518 (8.92 Mb) on chromosome 1 A, AX-95251140 (535.73 Mb) on 5 A, and AX-94589700 (469.37 Mb) on 6D were located in the vicinity of previously reported markers AX-95652739 at 7.64 Mb (Gaur et al. 2024), AX-95257620 at 533.29 Mb (Nawade et al. 2025), and AX-111919223 at 466.89—472.91 Mb (Ding et al. 2023), respectively. The remaining MTAs appear to represent novel loci.

For FLA, 17 stable MTAs were identified. Among these, AX-94486441 (702.11 Mb) on chromosome 2 A and AX-95630121 (708.68 Mb) on chromosome 4 A co-localizes with previously reported markers AX_109951135 (716.33 Mb) and AX_94484676 (709.66 Mb), respectively (Chen et al. 2021). Chromosome 5 A emerged as a major region for FLA in the present study, with six stable MTAs detected within the 608.34–613.54 Mb interval. This region overlaps with the meta-QTLs MQTL-5A.6 (601.39–661.14 Mb) and MQTL-5A.7 (613.58–653.85 Mb) reported by Kong et al. (2023). In addition, AX-94987657 (495.03 Mb) on chromosome 5D was located near marker D_GBF1XID01CVZMX_132 (Gao et al. 2021), and AX-94422554 (686.69 Mb) on chromosome 6B overlapped with MQTL-6B.5 (Kong et al. 2023). The remaining seven MTAs appear to represent potentially novel loci.

For PH, 16 stable MTAs were identified. AX-95630121-4A (708.68 Mb) was located near AX-109845420 at 716.97 Mb (Hu et al. 2021) and S4A_710817792 at 710.81 Mb (Jamil et al. 2019). Similarly, AX-94934730 on chromosome 5 A (608.62 Mb) was found in proximity to RAC875_c50143_604 at 617.98 Mb (Gao et al. 2021), BobWhite_c15758_79 at 595.37 Mb (Tian et al. 2023), and S5A_625839432 at 625.83 Mb (Jamil et al. 2019). AX-95630592 on chromosome 6B (686.80 Mb) was close to AX-108888670 at 690.02 Mb (Hu et al. 2021). Likewise, AX-94477352 on chromosome 7 A (628.94 Mb) and AX-95203773 on chromosome 7D (7.28 Mb) were located near S7A_618498297 at 618.50 Mb (Jamil et al. 2019) and RAC875_rep_c112729_702 at 8.34 Mb (Tian et al. 2023), respectively.

For PDL, nine stable MTAs were detected, of which three were pleiotropic with PH, indicating a shared genetic basis between peduncle length and plant height. AX-94486441 on chromosome 2 A (702.11 Mb), AX-94658866 on chromosome 3B (789.75 Mb), AX-95630121 on chromosome 4 A (708.68 Mb), and AX-95207937 on chromosome 7D (6.13 Mb) were located near previously reported markers Excalibur_c7282_285-2A at 710.10 Mb, RAC875_c2340_616-3B at 796.31 Mb, BS00039641_51-4A at 702.07 Mb, and S7D_5101668-7D at 5.10 Mb, respectively (Sallam et al. 2026). The remaining five MTAs represent potentially novel loci for PDL.

For SL, eight stable MTAs were identified. AX-94720138 on chromosome 1 A at 579.98 Mb was located near BobWhite_c6664_644 at 574.94 Mb (Liu et al. 2018a), and AX-94454534 on chromosome 1B at 523.59 Mb was found near AX-95255966 (Hu et al. 2021). AX-95094133 on chromosome 4B at 416.30 Mb was located close to Tdurum_contig52805_183 at 404.51 Mb (Gao et al. 2021), while AX-94696367 on chromosome 7D at 550.85 Mb was positioned near D_contig37514_120 at 568.95 Mb (Tian et al. 2023).

For SNS, 16 stable MTAs were detected. AX-94496249 on chromosome 3 A at 506.43 Mb co-localized with Kukri_c7218_1145 at 504.8 Mb (Liu et al. 2018a). AX-94702170 on chromosome 3B at 9.69 Mb was located near Tdurum_contig28100_112 at 10.75 Mb and BobWhite_c15529_288 at 10.41 Mb (Sun et al. 2017). On chromosome 3D, AX-94599436 (412.09 Mb) and AX-94872610 (416.58 Mb) were positioned close to AX-94648607 at 419.90 Mb (Li et al. 2019a, b). The remaining 12 MTAs on chromosomes 1B, 3 A, 3B, 6B, and 7D appear to be novel, although previous studies have reported associations on these chromosomes (Eltaher et al. 2022; Malik et al. 2021; Xu et al. 2024), highlighting their importance for SNS.

For GPS, eight stable MTAs were identified, many of which showed pleiotropic effects with other spike-related traits. AX-94858787-3A on chromosome 3 A at 131.46 Mb was located near wsnp_Ex_c1763_3333974 at 134.60 Mb (Liu et al. 2018a). The remaining MTAs on chromosomes on 1 A, 1B, 3D, 4B, 6B and 7D represent potentially novel loci, as these genomic regions have not been previously reported for association with GPS. However, prior studies have reported MTAs on these particular chromosomes (Sheoran et al. 2022; Zheng et al. 2022).

For TGW, 15 stable MTAs were identified. AX-94454534 on chromosome 1B at 523.60 Mb was located near wsnp_Ex_c1600_3051075 at 524.15 Mb (Schierenbeck et al. 2021). AX-94433908 on chromosome 4 A at 681.36 Mb was only 20 kb away from S4A_681180933 (Bhatta et al. 2018). AX-95094133 on chromosome 4B at 416.31 Mb was positioned near S4B_407743714 at 407.74 Mb (Bhatta et al. 2018). On chromosome 5B, three stable MTAs, AX-94384625 (122.42 Mb), AX-94432536 (545.80 Mb), and AX-95077961 (458.05 Mb) were located near qTGW5B.2 at 114.40–211.80 Mb (Pang et al. 2020), AX-109312888 at 545.17 Mb (Li et al. 2019a, b), and RAC875_c9150_2945 at 459.48 Mb (Schierenbeck et al. 2021), respectively. Additionally, AX-95091214 on chromosome 6B and AX-95209949 on chromosome 7 A co-localized with Qtgw.ahau-6B.1 (Cao et al. 2020) and wsnp_Ex_c2268_4251636 (Liu et al. 2018a), respectively. The remaining MTAs appear to represent novel loci for TGW.

For YPP, nine stable MTAs were identified. AX-94725733 on chromosome 1B at 152.49 Mb was located near S1B_164107163 at 164.10 Mb (Jamil et al. 2019). AX-95679601 on chromosome 1B at 201.44 Mb was close to Tdurum_contig6949_697 at 208.49 Mb (Ahmed et al. 2023). AX-94702170 on chromosome 3B at 9.69 Mb was positioned near Yld.cim-3BS.2 at 5.60 Mb (Juliana et al. 2021). AX-95165912 on chromosome 4B at 599.22 Mb was approximately 8 Mb from 4B_608802048_608802085 at 608.88 Mb (Chidzanga et al. 2022). AX-94432536 on chromosome 5B at 545.80 Mb was located near Yld.cim-5BL.2 at 550.91 Mb (Juliana et al. 2021). The remaining four MTAs appear to be potentially novel for YPP.

Several stable pleiotropic MTAs were identified for multiple traits, including DTF, DTM, SL, SNS, GPS, TGW, and YPP. Similar pleiotropic loci associated with yield-related traits have been reported in earlier GWAS studies (Liu et al. 2018b, 2018c; Ren et al. 2021; Alemu et al. 2021; Bennani et al. 2022). Such multi-trait loci are particularly valuable for breeding programs, as they may enable the simultaneous improvement of multiple agronomic traits. Furthermore, the use of high-density SNP markers, high − log₁₀(P) values, and the co-localization of identified MTAs with previously reported QTLs/MTAs collectively increase confidence that these associations represent true genomic regions for studied traits and should be considered promising targets for future breeding efforts.

Haplotyping of SNP markers, rather than reliance on single SNPs, improves the predictive power of biallelic markers (Alemu et al. 2023). Identification of haplotype blocks in wheat is therefore essential for genomic studies, as it provides a better understanding of genetic diversity and increases the efficiency of marker-assisted and genomic selection strategies. Consistent with earlier studies reporting superior haplotypes associated with stress tolerance, yield, and quality traits (Wang et al. 2017a, b; Swamy et al. 2017; Luján Basile et al. 2019), the present study identified 32 haplotype blocks that significantly influenced the evaluated traits. Genotypes carrying favourable haplotypes exhibited improved cold tolerance and higher yield potential compared with those carrying non-favourable haplotypes, highlighting the effectiveness of integrating germplasm clustering with haplotype-based analysis for the identification of superior breeding lines.

LD decay in wheat varies across populations, as reported in several studies (Lin et al. 2019; Devate et al. 2022; Rathan et al. 2022; Gudi et al. 2023; Kumar et al. 2025). In the present study, faster LD decay was observed in the A sub-genome (5.2 Mb), while relatively slower decay was detected in the B (5.7 Mb) and D (6.9 Mb) sub-genomes. Similar LD patterns across the wheat sub-genomes have been reported in earlier studies (Devate et al. 2022; Gudi et al. 2023). The differences in LD decay among the A, B, and D sub-genomes suggest that these genomes, along with their diploid progenitors, experienced distinct evolutionary histories and selection pressures during domestication and breeding. Additionally, we identified a large variation in LD decay at the chromosome level, with faster decay in chromosome 6 A (0.73 Mb) and slower decay in chromosome 2 A (7.42 Mb), indicating a differential rate of recombination among the chromosomes.

Furthermore, the identification of putative CGs is a crucial step in elucidating the biological mechanisms underlying the detected MTAs. High-confidence MTAs identified through GWAS are known to serve as potential targets for extracting CGs associated with the traits of interest (Gudi et al. 2024; Rajamanickam et al. 2024; Gudi et al. 2025a, b). In line with this, the present study utilized high-confidence MTAs identified through GWAS to extract putative CGs associated with the studied traits.

In silico expression analysis and a comprehensive literature survey highlighted genes potentially associated with cold tolerance, thereby strengthening our understanding of the underlying regulatory genetic pathways. Important CGs for CS located in the vicinity of stable MTAs, based on chromosome-specific LD decay, are discussed below.

The MTA AX-95172504 on chromosome 1B encodes a UDP-glucosyltransferase gene (TraesCS1B02G08040), whose expression was 6.68-fold higher under cold-stress conditions compared with the control. Similar expression patterns of glycosyltransferase genes have been reported as highly upregulated during cold-stress responses in wheat (Zhao et al. 2020b). Likewise, AX-94471137 on chromosome 1D encodes a chlorophyll a–b binding protein, and its transcript showed strong upregulation under stress conditions. Previous studies have reported that overexpression of chlorophyll a–b binding proteins alleviates cell membrane damage caused by low-temperature stress and enhances cold tolerance (Skinner 2015; Liu et al. 2025a, b, c). On chromosome 3B, AX-94754126 encodes a MYC-type bHLH domain protein (TraesCS3B02G018400), which was upregulated 2.27-fold under stress conditions compared with the control. The regulatory role of bHLH transcription factors in cold tolerance has been demonstrated in several transcriptome-based studies (Tian et al. 2022; Li et al. 2023a, b). On chromosome 5 A, AX-95228006 encodes a COBRA-like protein (TraesCS5A02G392000), with expression levels 1.49-fold higher under stress conditions. Previous expression analyses have highlighted the importance of COBRA genes in cold responses through their role in regulating cell wall biosynthesis (Liu et al. 2023). The same locus also encodes an ABC-transporter-like protein (TraesCS5A02G392600), which exhibited 3.92-fold higher expression under stress conditions. ABC transporters have also been reported to be highly enriched at 0 and − 5 °C in a previous study by Tian et al. 2022. In addition, 16 CGs were identified as potentially involved in cold-stress signaling pathways based on previously reported literature and their functions are discussed in Table S21.

KnetMiner analysis identified 21 gene networks associated with yield and yield-related traits, encompassing multiple genes and interconnected regulatory pathways. Among these, a CG, TraesCS6A02G373000, corresponding to the rice ortholog OsCHR, was identified underlying a FLA-associated MTA (AX-94779816) on chromosome 6A. Guo et al. (2022a, b) reported that OsCHR707 participates in leaf and reproductive development. Another CG, TraesCS5A02G420200, corresponding to TaPIF4, was identified underlying a PH-associated MTA (AX-94934730) on chromosome 5A. Overexpression of TaPIF4 has been reported to increase plant height in wheat (Zhang et al. 2022a, b; Dong et al. 2023), whereas knockout of TaPIF4 results in a semi-dwarf phenotype (Cao et al. 2024). Additionally, three CGs associated with spike length-TraesCS1A02G420600, TraesCS1B02G300500, and TraesCS3D02G395100, located on chromosomes 1 A, 1B, and 3D, respectively, correspond to MYB80. Browne et al. (2018) reported that MYB80 plays a crucial role in tapetal programmed cell death and microspore maturation, processes that are tightly synchronized with spike growth and floral differentiation. Since spike elongation reflects the progression of reproductive developmental stages, the involvement of MYB80 in this network provides functional support for the identified association. The co-expression of MYB80 with genes related to cell proliferation and reproductive tissue differentiation suggests that variation at this locus may influence spike length through its role in regulating anther development and overall floral organ growth.

Additional potential CGs related to yield were also identified through extensive literature mining, which warrants further discussion (Table S21). Glycosyltransferases regulate flowering time through glycosylation-mediated modulation of flowering pathways, including control of the floral repressor FLOWERING LOCUS C (Wang et al. 2012a, b). WD40 repeat proteins act as positive regulators of flowering time by activating heading-date genes in photoperiod-responsive pathways (Zhang et al. 2023c). MADS-box transcription factors are a well-documented group of genes that regulate flowering and maturity by specifying floral organ identity through combinatorial interactions of A, B, C, D, and E class genes, thereby controlling the development of sepals, petals, stamens, carpels, and ovules (Ali et al. 2019; Pang et al. 2020). The ubiquitin/SUMO-activating enzyme regulates maturity by controlling phytohormone-mediated protein turnover during developmental transitions from flowering to final maturation (Yue et al. 2025). The LITTLE ZIPPER protein regulates flag leaf area by modulating HD-ZIPIII activity, forming heterodimers with REV to control leaf polarity and vascular differentiation (Wenkel et al. 2007). Leucine-rich repeat domains regulate leaf development by mediating signaling pathways that control leaf growth, morphology, and photosynthetic efficiency (Ding et al. 2024a, b). bHLH domain proteins regulate plant height by controlling gene networks involved in cell elongation, growth, and developmental responses (Song et al. 2025). F-box domain proteins negatively regulate plant height, where overexpression reduces plant height and RNAi-mediated suppression increases it (Sun et al. 2024). Protein kinase domain-containing genes are significantly associated with plant height, peduncle length, and penultimate node length (Miao et al. 2017). Homeodomain ZF-HD class proteins regulate spike development, flowering time, and frost tolerance, thereby influencing spike morphology and seed traits under stress conditions (Kovalchuk et al. 2016).

Lateral organ boundaries domain proteins influence spike length and plant architecture, likely through the auxin signaling pathway (Wang et al. 2022a, b). DnaJ domain genes are significantly associated with spike development and related traits in wheat (Malik et al. 2021). Glycosyltransferase 61 influences thousand-grain weight in wheat based on orthologous rice genes (Miao et al. 2022). Cytochrome P450 genes regulate grain weight by controlling grain size (Guo et al. 2021). Zinc-finger C2H2-type proteins maintain grain yield while regulating heading date in wheat (Li et al. 2023a, b). The PPC domain increases thousand-grain weight and yield by regulating spike and grain development in wheat (Guo et al. 2025).

Collectively, these promising CGs represent valuable targets for future wheat breeding programs. The development of breeder-friendly KASP markers targeting these loci would facilitate their efficient deployment in marker-assisted selection and genomic breeding strategies.

## Conclusion

This study investigated the genetic basis of cold tolerance and yield-related traits in synthetic-derived introgression lines evaluated under temperate conditions of the north-western Himalayas. A total of 152 stable MTAs were identified for cold tolerance indices and key agronomic traits, several of which also surpassed the Bonferroni-adjusted significance threshold and were classified as high-confidence loci. A number of stable MTAs also co-localized with previously reported QTLs/MTAs, whereas others represent potentially novel loci, thereby enriching the current understanding of the genetic architecture of cold adaptation.

Candidate genes located within chromosome-specific LD intervals of high-confidence MTAs included transcription factors, protein kinases, and stress-responsive regulators, highlighting their roles in coordinating cold tolerance and yield stability. Literature mining, in silico expression profiling, and network analyses further supported the functional relevance of these genes in cold stress response and productivity-related processes.

Importantly, elite lines carrying favourable haplotypes for early maturity, enhanced cold tolerance, and higher yield potential were identified, offering valuable genetic resources for breeding programs in cold-prone regions such as Kashmir, and supporting the sustainability of rice–wheat cropping systems. Future efforts should focus on validating these MTAs and haplotypes through fine-mapping, functional genomics, and genome-editing to accelerate their use in marker-assisted and genomic selection pipelines. Integrating haplotype-based breeding with high-throughput phenotyping and predictive models will further enable pyramiding of favourable alleles for cold resilience and yield stability. Such integrative approaches hold promise for developing climate-resilient wheat cultivars, ensuring sustainable productivity in vulnerable agroecosystems.

## Supplementary Information

Below is the link to the electronic supplementary material.

Supplementary file1 (DOCX 66860 KB)

Supplementary file2 (XLSX 11.5 MB)

## Data Availability

All phenotypic, genotypic and other data supporting the findings of this study are included within the manuscript and its supplementary materials.
