# Supplementary material for: Genetic dissection of cold stress tolerance and yield potential under cold stress in nested synthetic wheat (Triticum aestivum L.) introgression libraries using multi-locus genome-wide association and haplotype analysis
Source: Funct Integr Genomics. 2026 Jun 23;26(1):158. doi: 10.1007/s10142-026-01925-w (PMC13290807; doi:10.1007/s10142-026-01925-w)
Supplement: Supplementary file 1 — Supplementary file1 (DOCX 66860 KB) [file 10142_2026_1925_MOESM1_ESM.docx]

**Genetic Dissection of Cold Stress Tolerance and Yield Potential under Cold Stress in Nested Synthetic Wheat (Triticum aestivum L.) Introgression Libraries Using** **Multi-Locus Genome-Wide Association and Haplotype Analysis**

**Mukesh Rathore^1^, Nikita Aggarwal^1^, Abhishek Pandey^2^, Satinder Kaur^2*^, Mohd. Ashraf Bhat^1^, Sundeep Kumar^3^, Mohd Anwar Khan^1^, Parvaze Ahmad Sofi^1^, Reyazul Rouf Mir^1&4*#^**

^1^Division of Genetics & Plant Breeding, Faculty of Agriculture (FoA), SKUAST-Kashmir, Wadura, Campus, Sopore-193201, Kashmir, J&K, India.

^2^School of Agricultural Biotechnology, Punjab Agricultural University, Ludhiana, 141004, India

^3^ICAR-National Bureau of Plant Genetic Resources (NBPGR), Pusa Campus, New Delhi, India

^4^Centre for Crop and Food Innovation, WA State Agricultural Biotechnology Centre, Murdoch University, Murdoch WA 6150, Australia

Mukesh Rathore: <https://orcid.org/0009-0002-7083-9576>

*****Satinder Kaur: https://orcid.org/0000-0003-3704-3074

Sundeep Kumar: <https://orcid.org/0000-0001-5917-8290>

*****Reyazul Rouf Mir: <https://orcid.org/0000-0002-3196-211X>

*** = Corresponding author email: satinder.biotech@pau.edu**

**Dr. Satinder Kaur**

School of Agricultural Biotechnology, Punjab Agricultural University, Ludhiana, 141004, India

*** = Corresponding author email:** [imrouf2006@gmail.com](mailto:imrouf2006@gmail.com)

**Dr. Reyazul Rouf Mir**

Division of Genetics & Plant Breeding, Faculty of Agriculture (FoA), SKUAST-Kashmir, Wadura Campus, Sopore-193201, Kashmir, J&K, India.

**^#^Present address:** Centre for Crop and Food Innovation, WA State Agricultural Biotechnology Centre, Murdoch University, Murdoch WA 6150, Australia

**Fig. S1: Monthly trends in temperature and relative humidity during the wheat growing period across environments.** The graphs display maximum temperature (green line), minimum temperature (purple line), maximum humidity (red line), and minimum humidity (blue line) across months.


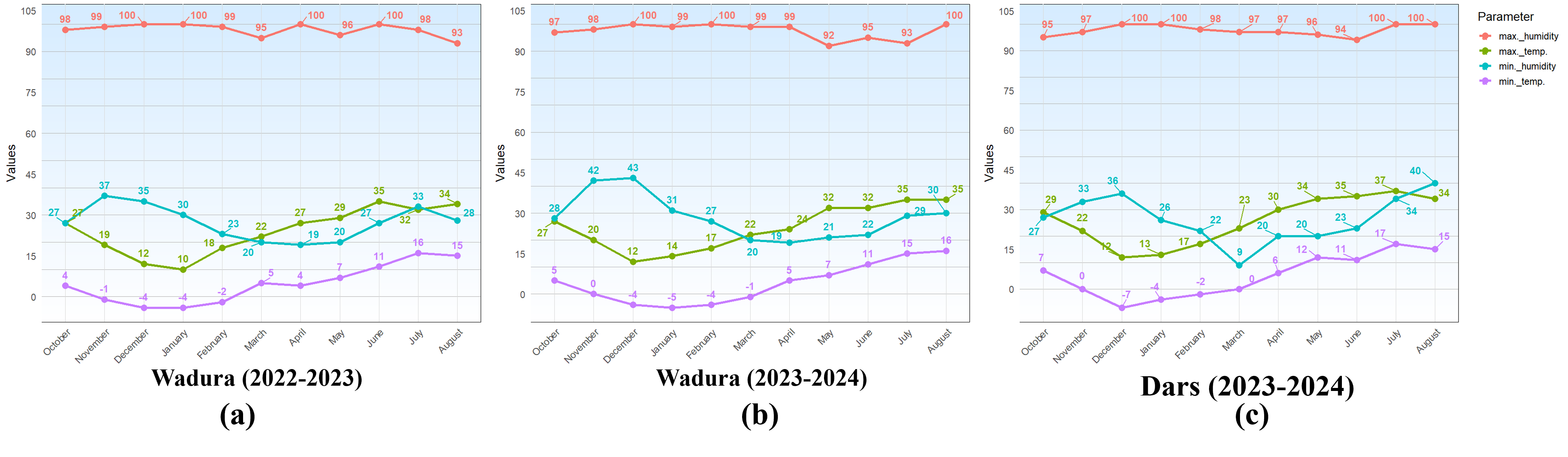


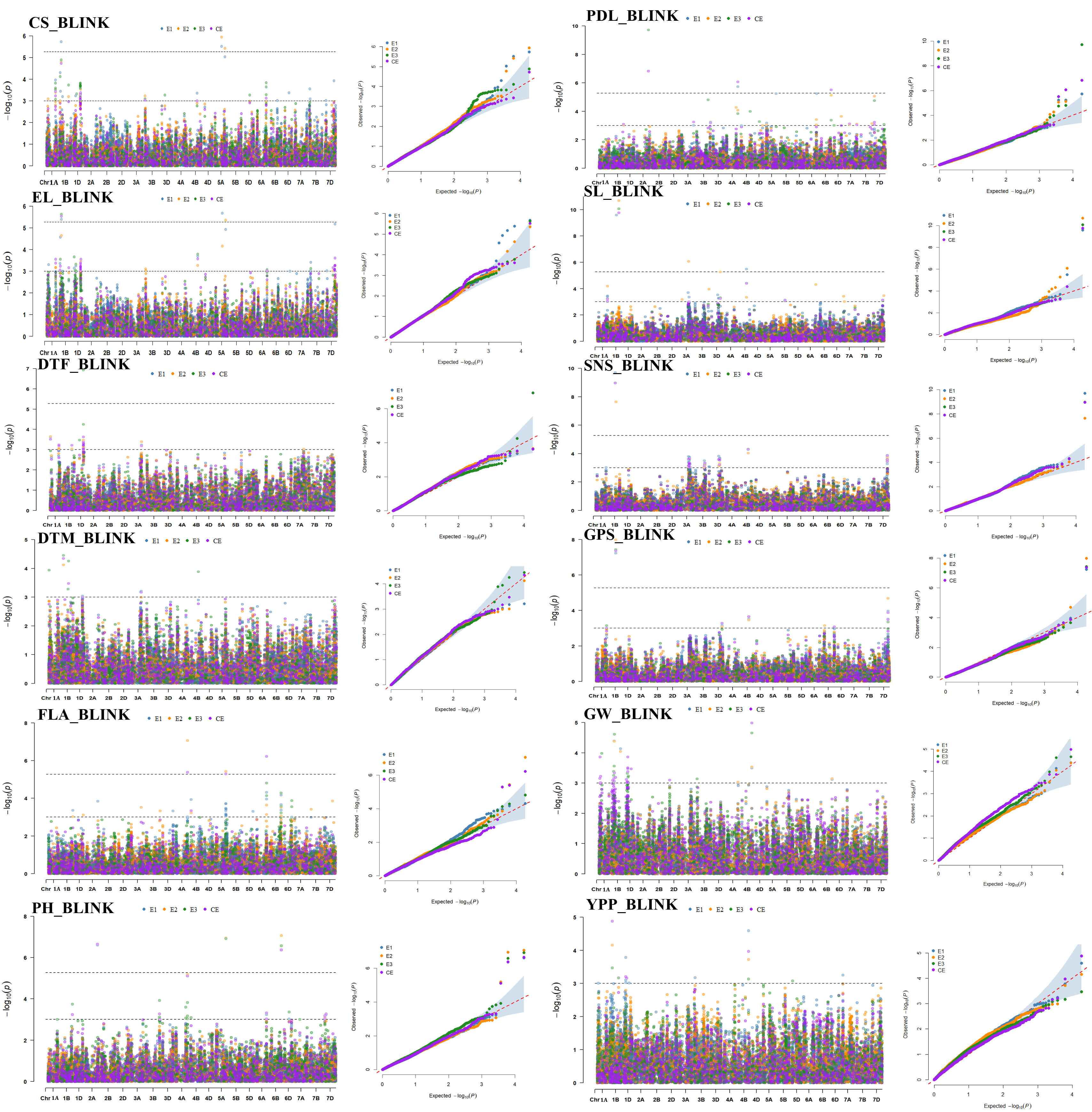
 **Fig. S2.** Manhattan and Quantile–quantile (Q–Q) plots showing GWAS results for cold stress tolerance (CS), electrolyte leakage (EL) and days to flowering (DTF) days to maturity (DTM) and flag leaf Area (FLA), plant height (PH) and peduncle length (PDL), spike length (SL) and number of spikelets per spikes (SNS), grain per spike (GPS) and thousand grain weight (TGW) and yield per plant (YPP) across environments (E1, E2, E3 and CE), using the BLINK model. The horizontal lines indicate the significance thresholds corresponding to −log₁₀P ≥ 3.00 and the Bonferroni-corrected genome-wide threshold of −log₁₀(P) ≥ 5.27.


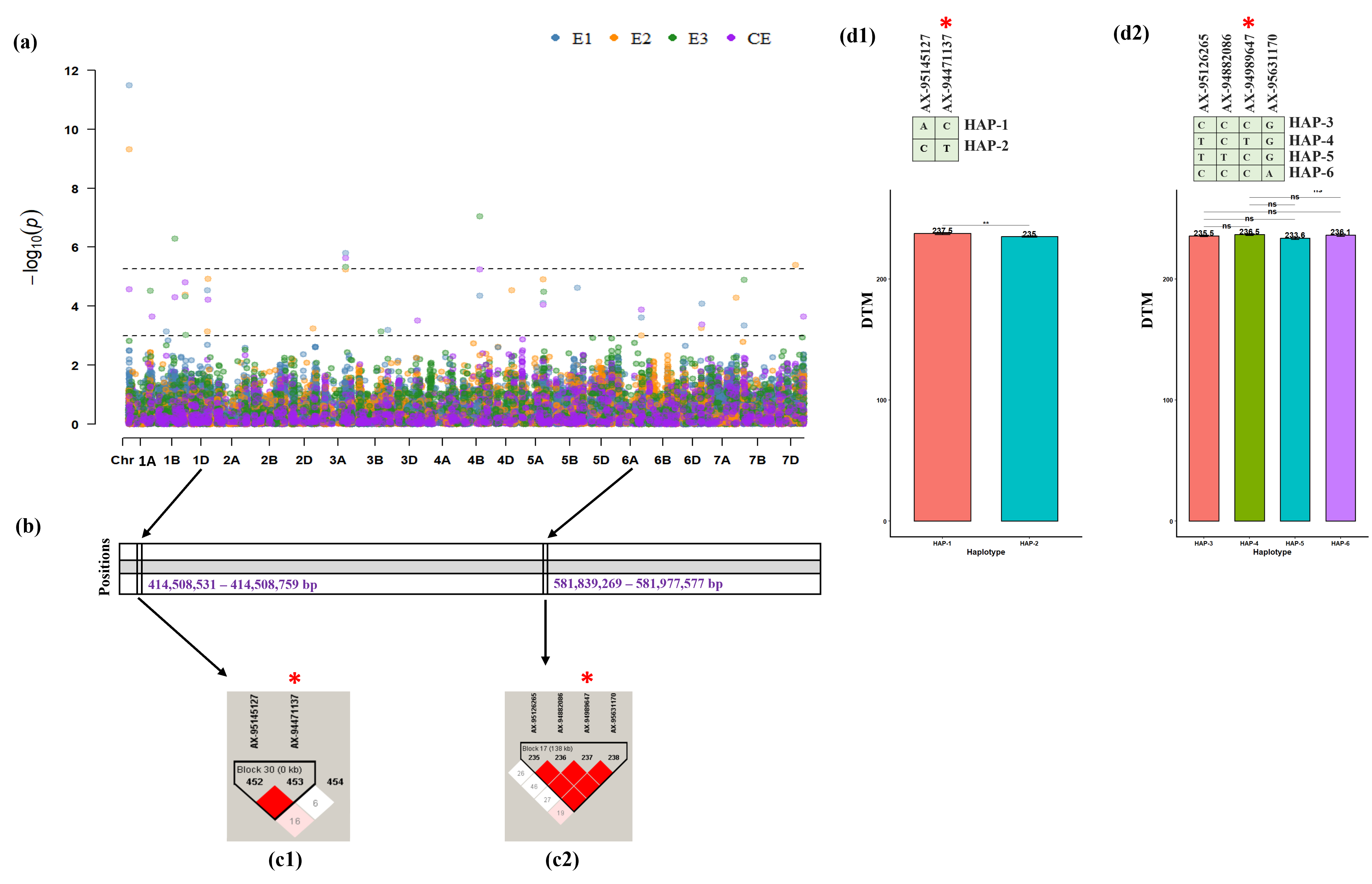
 **Fig. S3.** Significant haplotypes associated with days to maturity (DTM) on wheat chromosomes 1D and 6A. (a) Manhattan plot highlighting SNP associations for DTM across the 21 wheat chromosomes. (b) Range of physical positions of SNPs within each identified Linkage disequilibrium (LD) block. (c1 and c2) Show LD heatmaps among SNPs within haplotype blocks on chromosomes 1D and 6A. (d1 and d2) Illustrate the phenotypic variation in DTM among different haplotypes within each LD block.


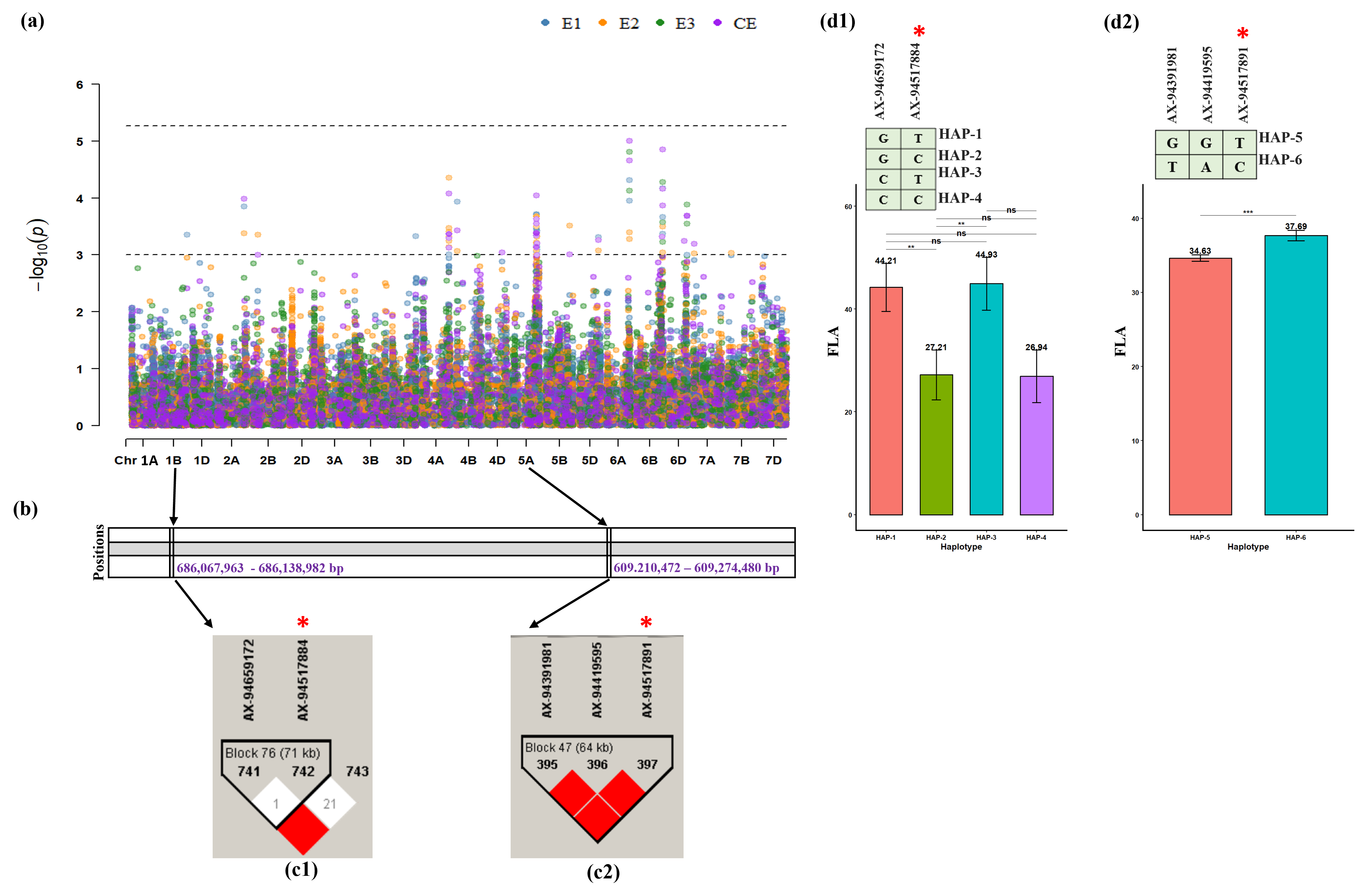
**Fig. S4.** Significant haplotypes associated with flag leaf area (FLA) on wheat chromosomes 1B and 5A. (a) Manhattan plot highlighting SNP associations for FLA across the 21 wheat chromosomes. (b) Range of physical positions of SNPs within each identified Linkage disequilibrium (LD) block. (c1 and c2) Show LD heatmaps among SNPs within haplotype blocks on chromosomes 1B and 5A. (d1 and d2) Illustrate the phenotypic variation in FLA among different haplotypes within each LD block.


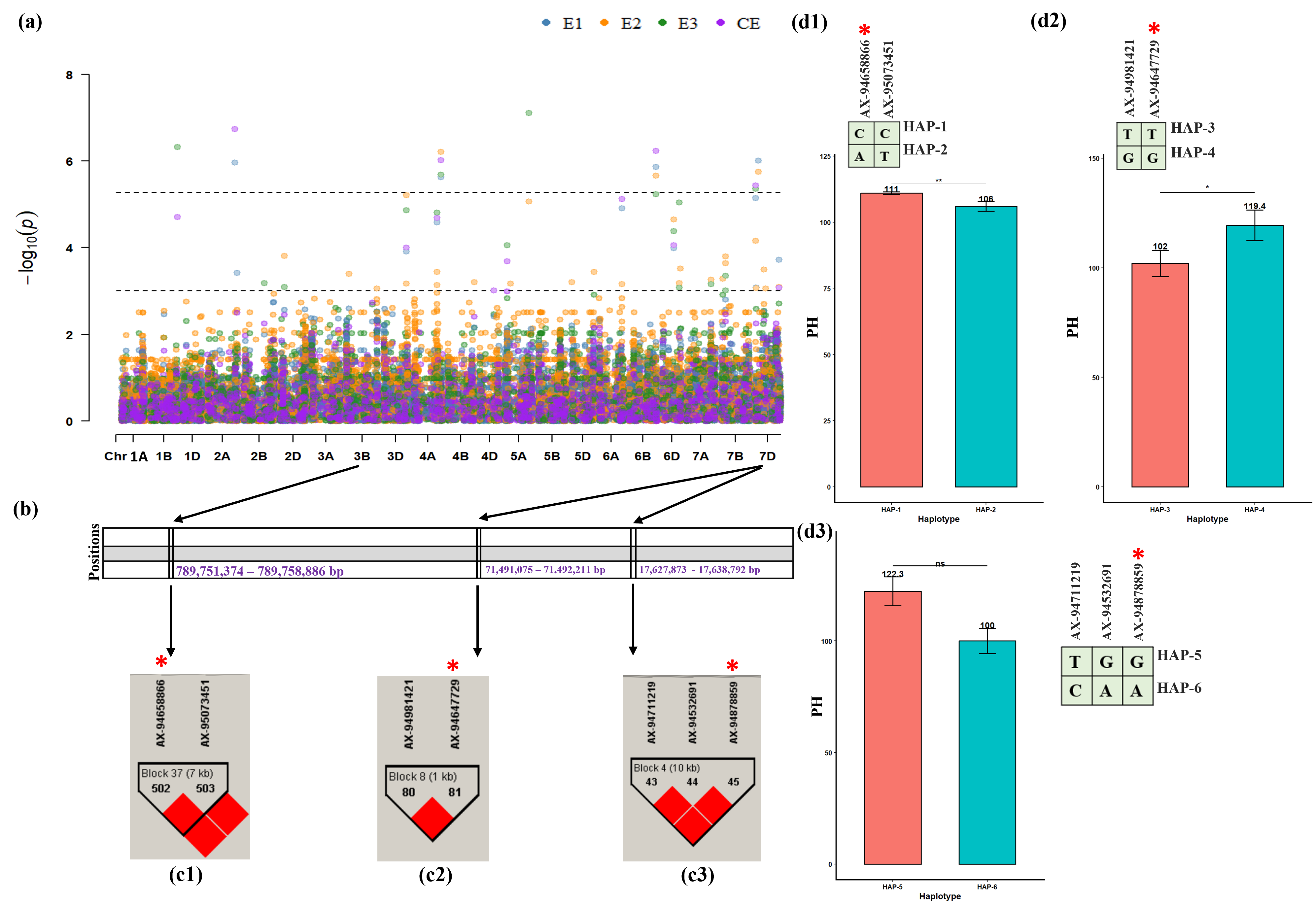
**Fig. S5.** Significant haplotypes associated with plant height (PH) on wheat chromosomes 3B and 7D. (a) Manhattan plot highlighting SNP associations for PH across the 21 wheat chromosomes. (b) Range of physical positions of SNPs within each identified Linkage disequilibrium (LD) block. (c1-c3) Show LD heatmaps among SNPs within haplotype blocks on chromosomes 3B and 7D. (d1-d3) Illustrate the phenotypic variation in PH among different haplotypes within each LD block.


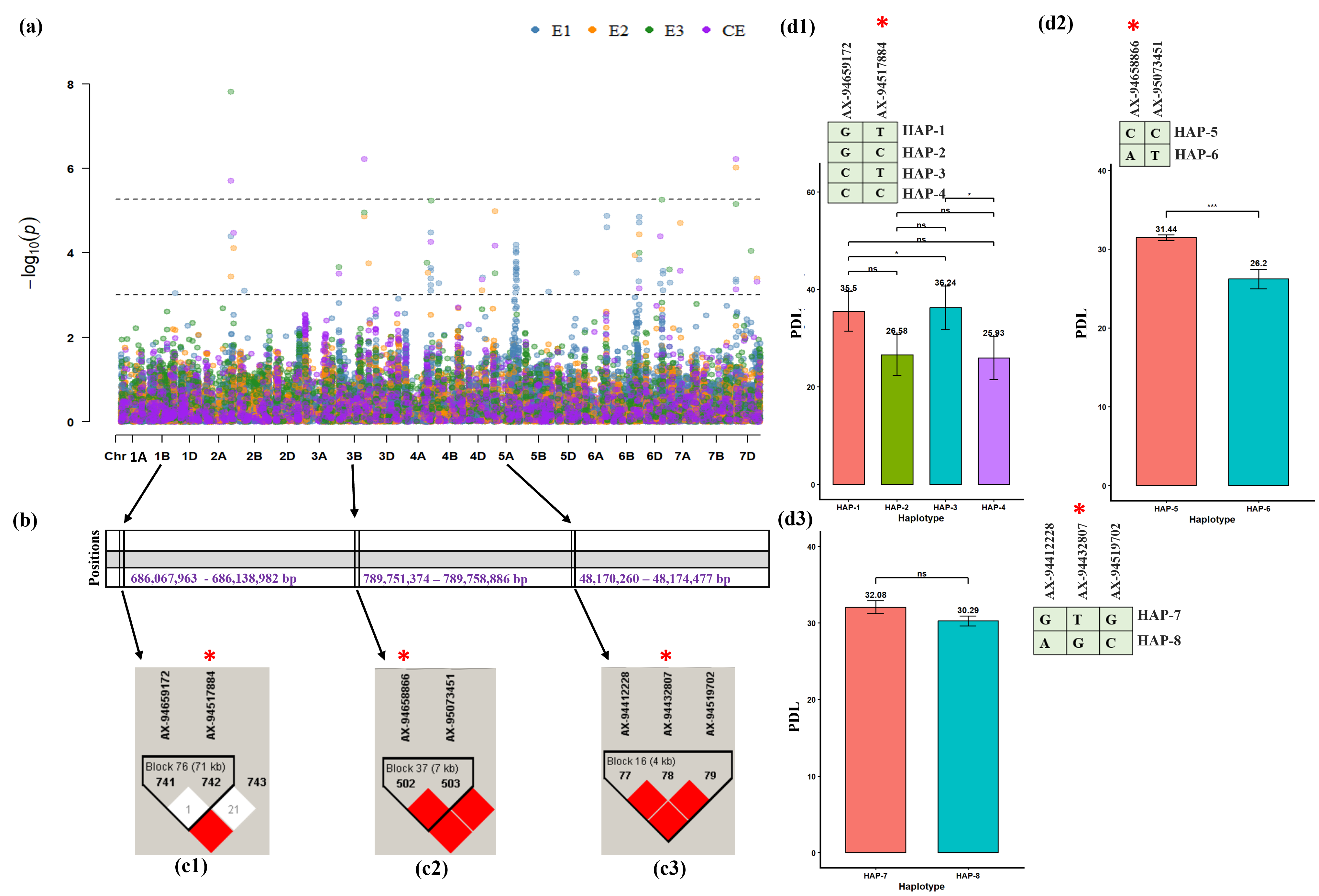
**Fig. S6.** Significant haplotypes associated with peduncle length (PDL) on wheat chromosomes 1B, 3B and 5A. (a) Manhattan plot highlighting SNP associations for PDL across the 21 wheat chromosomes. (b) Range of physical positions of SNPs within each identified Linkage disequilibrium (LD) block. (c1-c3) Show LD heatmaps among SNPs within haplotype blocks on chromosomes 1B, 3B and 5A. (d1-d3) Illustrate the phenotypic variation in PDL among different haplotypes within each LD block.


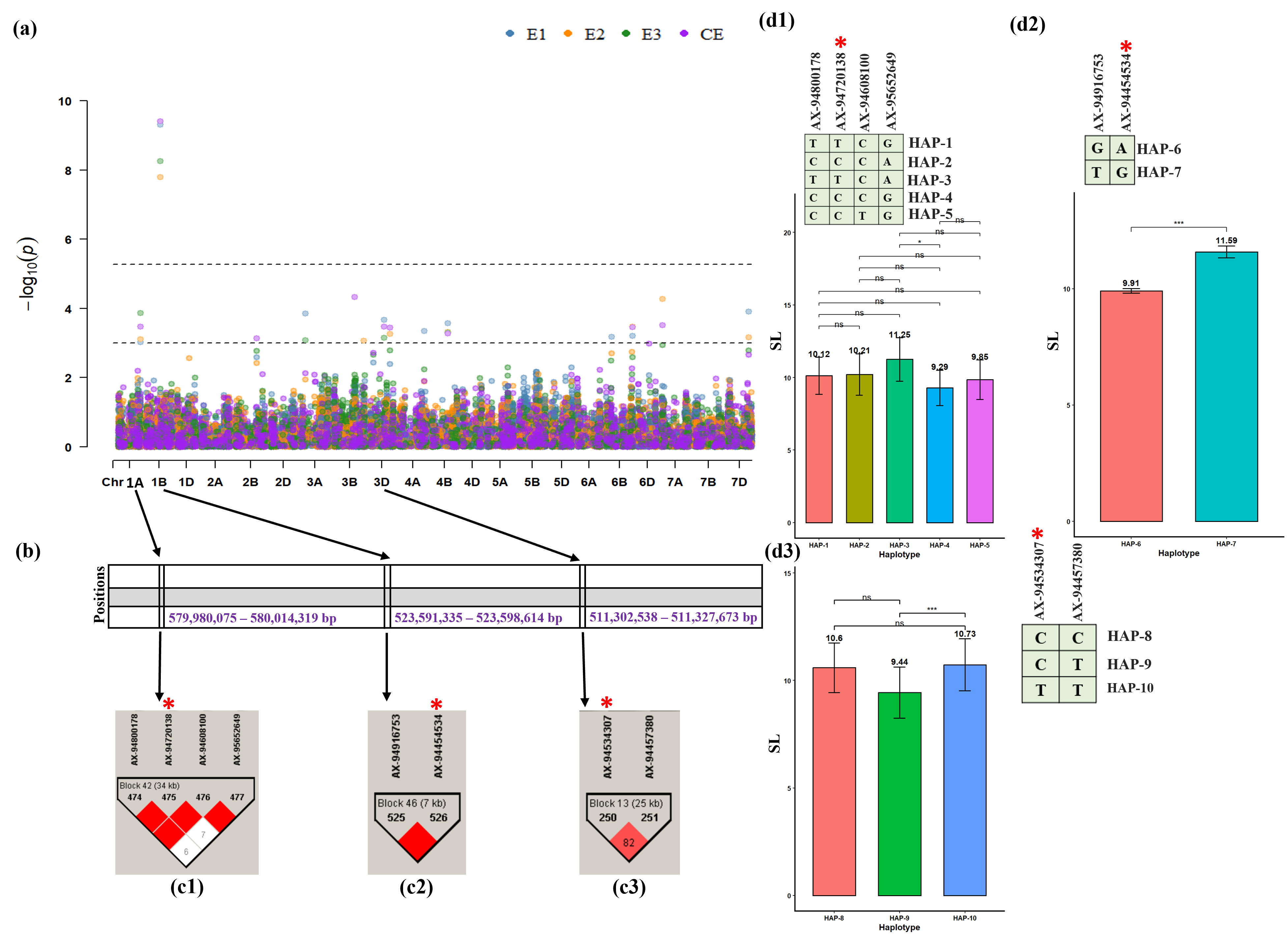
**Fig. S7.** Significant haplotypes associated with spike length (SL) on wheat chromosomes 1A,1B and 3D. (a) Manhattan plot highlighting SNP associations for SL across the 21 wheat chromosomes. (b) Range of physical positions of SNPs within each identified Linkage disequilibrium (LD) block. (c1-c3) Show LD heatmaps among SNPs within haplotype blocks on chromosomes 1A, 1B and 3D. (d1-d3) Illustrate the phenotypic variation in SL among different haplotypes within each LD block.


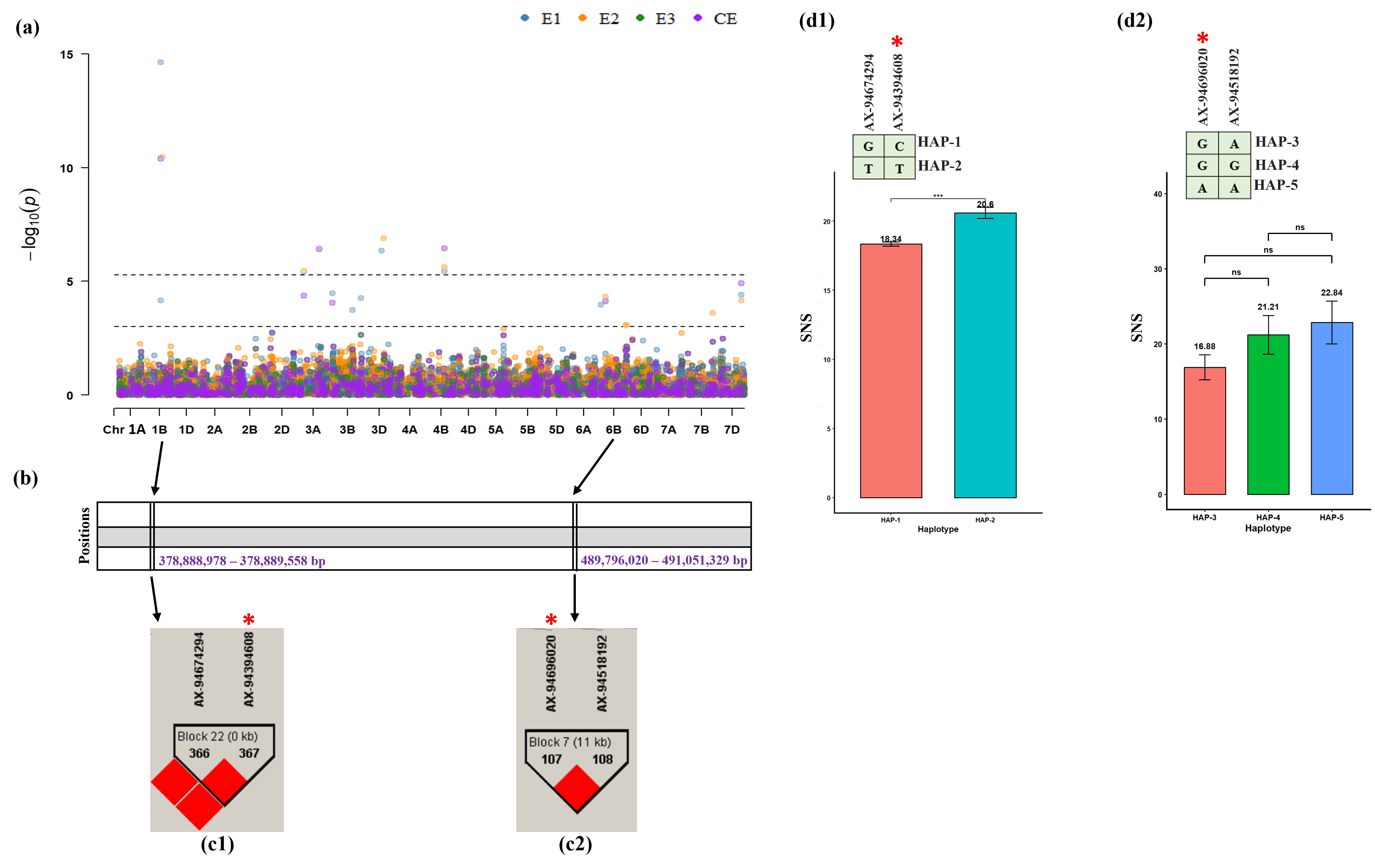
**Fig. S8.** Significant haplotypes associated with spikelets number per spikes (SNS) on wheat chromosomes 1B and 6B. (a) Manhattan plot highlighting SNP associations for SNS across the 21 wheat chromosomes. (b) Range of physical positions of SNPs within each identified Linkage disequilibrium (LD) block. (c1 and c2) Show LD heatmaps among SNPs within haplotype blocks on chromosomes 1B and 6B. (d1 and d2) Illustrate the phenotypic variation in SNS among different haplotypes within each LD block.


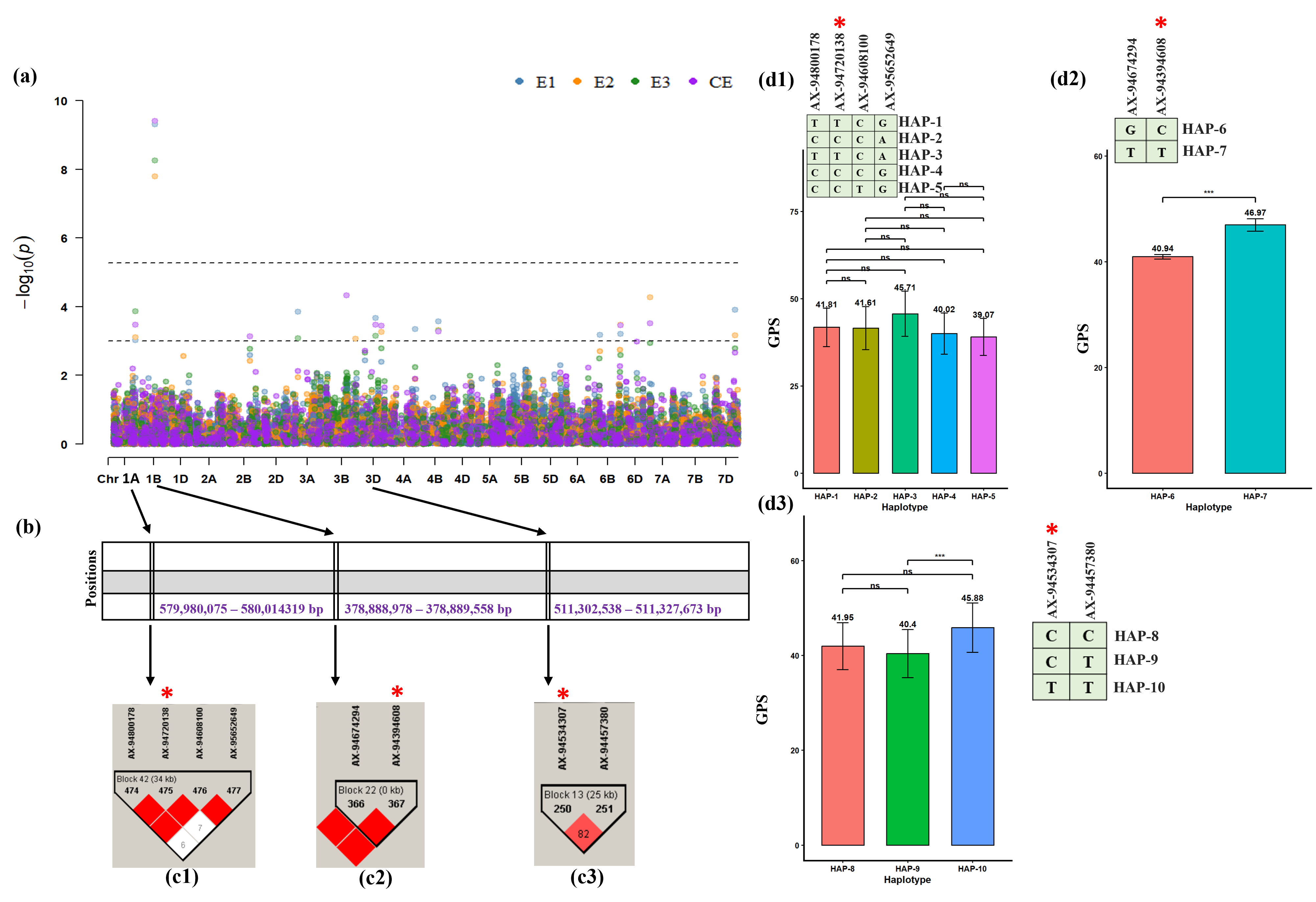
**Fig. S9.** Significant haplotypes associated with grain per spikes (GPS) on wheat chromosomes 1A, 1B and 3D. (a) Manhattan plot highlighting SNP associations for GPS across the 21 wheat chromosomes. (b) Range of physical positions of SNPs within each identified Linkage disequilibrium (LD) block. (c1-c3) Show LD heatmaps among SNPs within haplotype blocks on chromosomes 1A, 1B and 3D. (d1-d3) Illustrate the phenotypic variation in GPS among different haplotypes within each LD block.

**Fig. S10.** Significant haplotypes associated with thousand grain weight (TGW) on wheat chromosomes 1B and 5B. (a) Manhattan plot highlighting SNP associations for TGW across the 21 wheat chromosomes. (b) Range of physical positions of SNPs within each identified Linkage disequilibrium (LD) block. (c1-c3) Show LD heatmaps among SNPs within haplotype blocks on chromosomes 1B and 5B. (d1-d3) Illustrate the phenotypic variation in TGW among different haplotypes within each LD block.


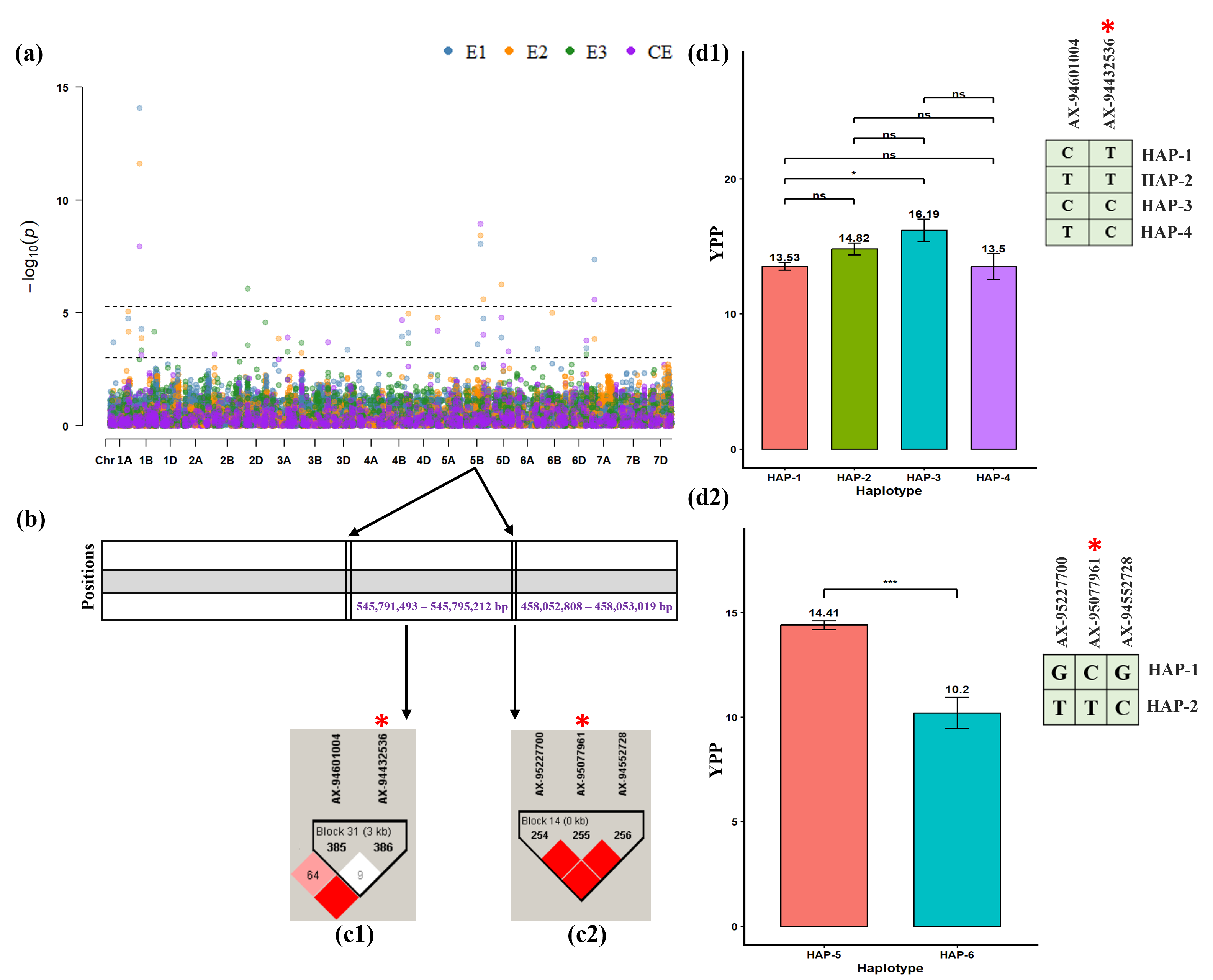


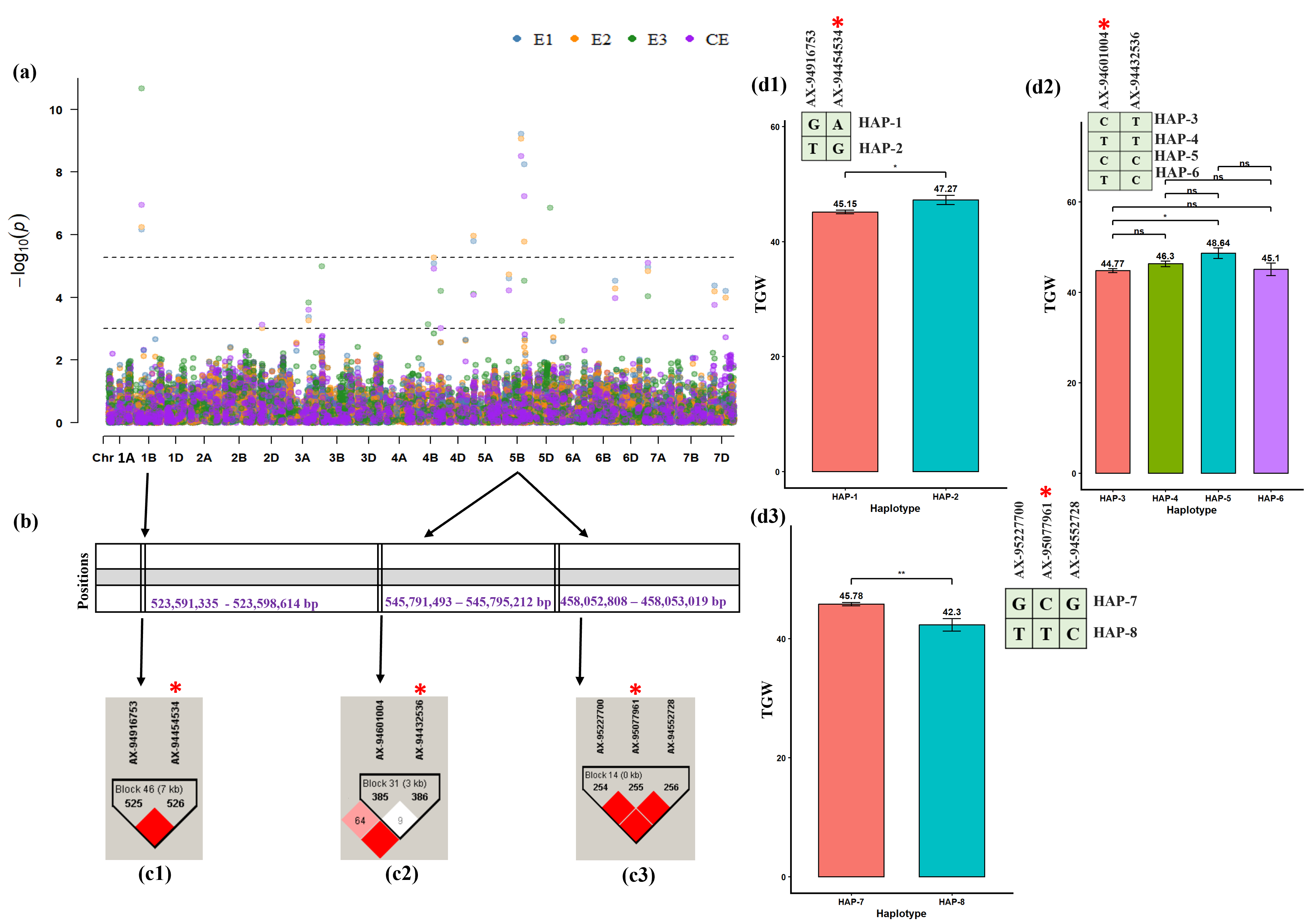
 **Fig. S11.** Significant haplotypes associated with yield per plant (YPP) on wheat chromosome 5B. (a) Manhattan plots highlighting SNP associations for YPP across the 21 wheat chromosomes. (b) Range of physical positions of SNPs within each identified Linkage disequilibrium (LD) block. (c1 and c2) Show LD heatmaps among SNPs within haplotype blocks on chromosome 5A. (d1 and d2) Illustrate the phenotypic variation in YPP among different haplotypes within each LD block.


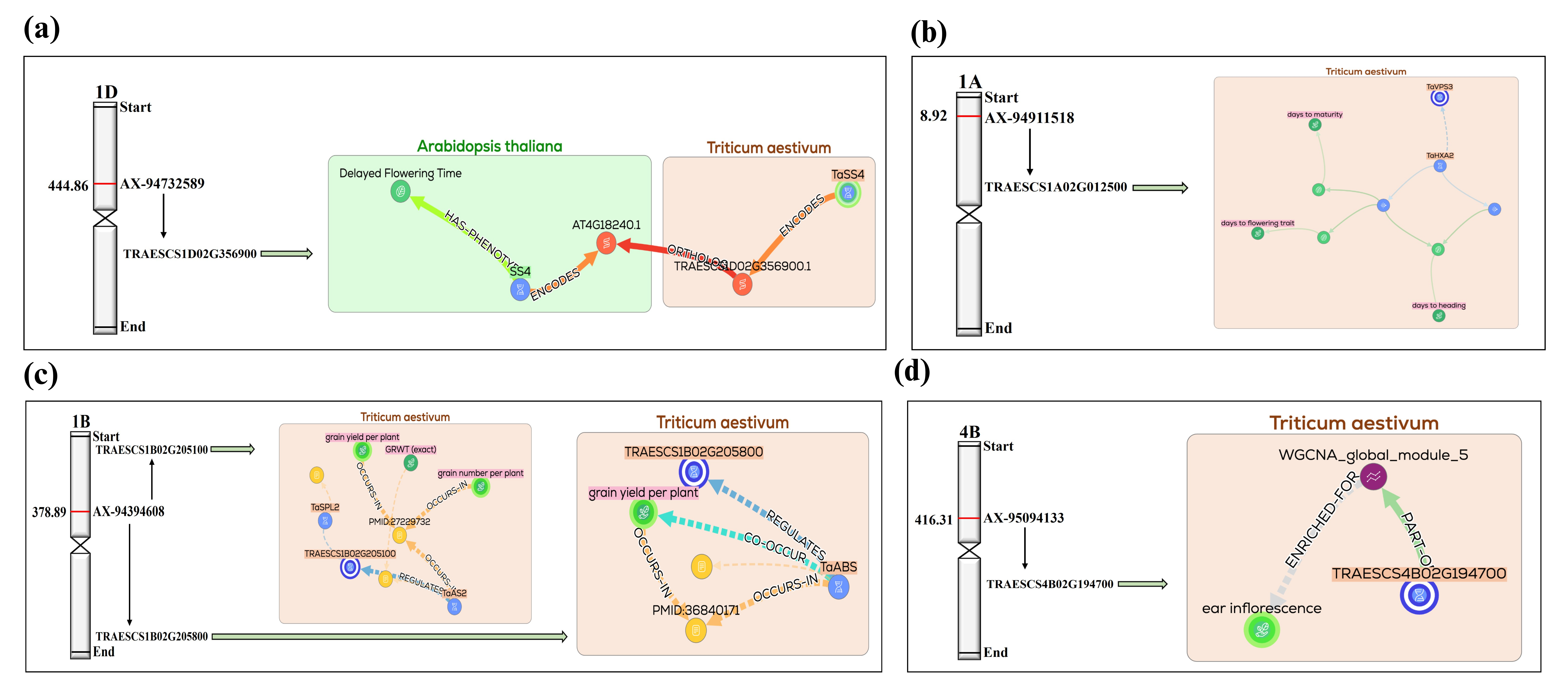
**Fig. S12.** Knowledge network generated using KnetMiner for days to flowering, days to maturity, number of spikelets per spike, and grains per spike, based on five candidate genes identified from four high-confidence MTAs.

**Fig. S13.** Knowledge network generated using KnetMiner for flag leaf area, plant height and peduncle length based on eight candidate genes identified from four high-confidence MTAs.






**Fig. S14.** Knowledge network generated using KnetMiner for spike length, thousand grain weight and yield per plant, based on eight candidate genes identified from five high-confidence MTAs.
